# Fast, Highly Stable, and Low-Bandgap 2D Halide Perovskite Photodetectors Based on Short-Chained Fluorinated Piperidinium as a Spacer

**DOI:** 10.1021/acsami.4c18202

**Published:** 2024-12-16

**Authors:** Norman Lu, Gurumallappa Gurumallappa, Jitendra Singh, Ka Long Chan, Eskedar Tessema, Pin-Yu Liu, Hema Mylnahalli Krishnegowda, Karthik Chimatahalli Shanthakumar, Jinn-Hsuan Ho, Yu-Chiang Chao, Lam-Gia-Hao Dao, Sumedh Shirsat, Meng-Lin Tsai

**Affiliations:** † Institute of Organic and Polymeric Materials, National Taipei University of Technology, Taipei 106, Taiwan; ‡ Graduate Institute of Energy and Optoelectronic Materials National Taipei University of Technology, Taipei 106, Taiwan (ROC); § Department of Physics, Udit Narayan Post Graduate College Padrauna Kushinagar, affiliated by Deen Dayal Upadhyaya Gorakhpur University, Kushinagar 274304, Uttar Pradesh, India; ∥ Department of Chemistry, SJCE, JSS Science and Technology University, Mysuru 570 006, Karnataka, India; ⊥ Department of Chemical Engineering, National Taiwan University of Science and Technology, Taipei 106335, Taiwan; # Department of Physics, National Taiwan Normal University, Taipei 106, Taiwan; ¶ Department of Materials Science and Engineering, National Taiwan University of Science and Technology, Taipei 106335, Taiwan

**Keywords:** (2D halide perovskite, noncovalent interactions (NCIs), fluorinated photodetector, tunable energy bandgap, fluorinated piperidinium organic spacer)

## Abstract

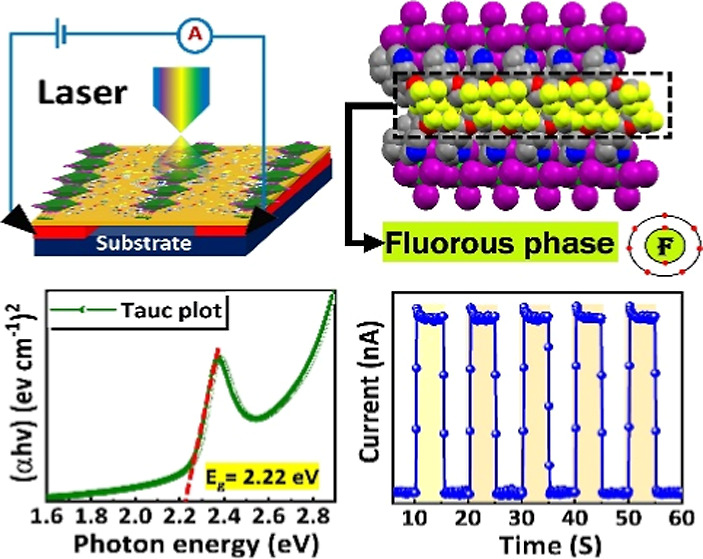

Ruddlesden–Popper (RP) two-dimensional (2D) halide perovskite (HP), with attractive structural and optoelectronic properties, has shown great potential in optoelectrical devices. However, the relatively wide bandgap (*E*_g_) and stability, which cause inferior efficiency, prevent its feasibility from further applications. To tackle these issues, for the first time, a novel fluorine-containing piperidinium spacer, (3-HCF_2_CF_2_CH_2_OCH_2_-PPH^+^), abbreviated as (4FH-PPH^+^), has been designed for the stable and efficient *n* = 1 2D HPs. Its fluorophobicity can significantly enhance the noncovalent interactions between cations and [PbI_6_]^4–^ octahedra. The observed *E*_g_ of the fluorinated (4FH-PPH)_2_PbI_4_ perovskite is found to be 2.22 eV, which is the lowest value among all fluorinated perovskites reported so far. Interestingly, this fluorinated film-based 2D perovskite photodetector (PD) exhibits the outstanding responsivity of 502 mA W^–1^, photodetectivity of 5.73 × 10^10^ cm Hz^1/2^ W^–1^, and impressive response/recovery time of 42/46 ms under 450 nm at 20 V. To the best of our knowledge, it is found for the first time that the 2D HP, with the fluorinated short-chained segment included into the organic spacer, shows remarkable stability for up to 49 days. These results strongly demonstrate the potential of the 2D fluorinated short-chained (4FH-PPH)_2_PbI_4_ HP with a low *E*_g_ as a promising candidate for next-generation optoelectronic devices.

## 1 Introduction

Recently, halide perovskites (HPs) have emerged as promising semiconducting materials and have been labeled as high-performance photovoltaic materials comparable to conventional silicon solar cells.^1−3^ They have attracted significant research attention among researchers due to their excellent structural flexibility, large absorption coefficients, narrow emission bandwidth, high carrier mobility, and low trap density.^4−6^ To date, three-dimensional (3D) organic–inorganic HPs have shown outstanding performance in various optoelectronic devices such as solar cells,^7^ detectors,^8^ light-emitting diodes,^9^ field effect transistors,^10^ and lasers.^11^ However, 3D organic–inorganic HPs usually show the poor resistance to moisture, light, and heat, which greatly limits their potential in practical applications.^12,13^ One of the viable strategies to tackle these issues is to incorporate the bulky hydrophobic organic monovalent or divalent cations into the 3D structure, followed by breaking down these structures into 2D forms like Ruddlesden–Popper (RP) phase^14^ and Dion-Jacobson (DJ) phase^15^ perovskites. The most common RP layered perovskites share the chemical formula of L_2_A_*n*–1_*M*_*n*_X_3*n*+1_ (*n* = 1, 2, ...), where L is the bulky organic cation.^16−18^ A is a small monovalent organic [CH_3_NH_3_^+^ and (NH_2_)_2_CH^+^] or inorganic (Cs^+^) cation, M is a divalent metal cation such as Pb(II) or Sn(II), X is a halide (Cl^–^, Br^–^, I^–^, or their mixtures), and n is the number of anionic octahedral layers.^19^

Researchers in the photodetector (PD) field have been increasingly focused on the popular lead-based HP materials owing to their outstanding carrier mobility, tunable bandgap (*E*_g_), low cost, long exciton diffusion length, simple fabrication process, and high light absorption coefficient.^20−22^ However, spectral selectivity, sensitivity, stability, and response speed are the crucial properties of the desired PD in the real-world applications.^23−25^ For their actual implementation, it is particularly challenging to maintain the stability of the PD device in a humid environment. During the past few years, there has been significant research interest in 2D RP HPs as highly promising materials for the fabrication of exceptional, long-term stabile, and high-performance PDs/solar cells. This is mainly due to the hydrophobic organic spacers and the well-aligned layered structures that can easily block the degradation of inorganic sheets from the influence of moisture and air.^26,27^ On the other hand, substituting the bulky organic cation in 2D HPs facilitates quantum/dielectric confinement effects that enhance photon emission in light-emitting devices. However, this confinement does not guarantee improved optoelectronic properties. In fact, the confinement can lead to a trade-off between photocurrent generation and stability, as carriers may easily recombine, limiting the efficiency in light-absorbing applications.^28,29^ So far, significant progress has been made in the area of optoelectronics, leading to remarkable accomplishments. However, the bandgaps of 2D RP HPs are usually larger than those of their 3D counterparts, limiting their absorption range and efficiency for broadband light harvesting/detection in the visible light region.

It is still necessary to search for new organic spacers, which can further enhance the efficiency and stability of optoelectronic devices in 2D RP HPs. One promising option is by using the fluorinated organic spacer cations, which can improve the moisture stability, crystal quality, charge separation ability, and carrier lifetime of these devices through interacting with the inorganic layers.^30−34^ In 2020, Lu’s group reported the fluorine-containing highly efficient PD using a single-crystal linear-chained polyfluorinated dibromo-platinum(II)diimine complex, which is an organometallic complex known to be air stable and to have good thermal stability. It shows a fast mobility up to 0.45 cm^2^ V^–1^ s^–1^, an excellent photoresponsivity of 1000 A W^–1^, and fast response and recovery times of 80 and 90 μs, respectively. More importantly, this device consistently performs even under the harsh environment, including more than 90 days in air and 24 h in water. The excellent performances and extraordinary stability are mainly due to the incorporation of fluorinated side-chains.^30^ Previously reported 2D DJ HPs with diammonium cations (+2) exhibit better stability compared with 2D RP HPs that contain the monovalent cations. Thus, the diammonium cations (+2) in 2D DJ HPs are able to be strongly bound by both electrostatic interactions and hydrogen bonds with inorganic sheets on both sides.^35^

Up to now, only 2D DJ HPs with piperidinium cations have been used in the development of PD devices. To our knowledge, there is only one report available regarding the synthesis of 2D RP HPs using monovalent piperidinium cations for this particular purpose.^36^ This may be due to difficulties in their preparation of piperidinium-based cations. In this context, there is an urgent need to design a novel monovalent piperidinium-based 2D RP perovskite PD that exhibits the high crystalline quality, the long-lasting stability, and the tunable *E*_g_ for their PD applications. Additionally, it has been found by Lu’s group from the noncovalent interaction studies that the terminal H–CF_2_ group is able to function as a hydrophobic hydrogen bonding donor without even carrying the ionic charge.^37^ It is known that this terminal H–CF_2_ group can form the C–H···X-M (metal-bound halide)^38^ blue-shifting hydrogen bonding (HB)^39^ and the common C–H···F blue-shifting HB while there is the fluorine atom(s) present in the organic spacer.

Herein, for the first time, we report an efficient and highly stable poly-fluorinated piperidinium-based 2D RP perovskite PD with the structure of (3-**HCF**_**2**_CF_2_CH_2_OCH_2_-**PPH**)_2_PbI_4_ being abbreviated as (4FH-PPH)_2_PbI_4_. It is believed that 4FH-PPH^+^, with both ends (highlighted in bold) of this organic spacer being the good HB donors, can serve as an unusual bifunctional linker to form the HBs with the inorganic moiety in the 2D HP with RP-type (ratio of L/MX_2_ = 2:1) while it is still monovalent. Of course, the HCF_2_ end-group forms the hydrophobic blue-shifting HB, which is expected to be very different from the normal hydrophilic HB. Thus, the incorporation of the HCF_2_ group not only is able to enrich the unique material properties but also helps the new RP-type of 2D HP to be moisture-tolerant.

Interestingly, the incorporation of this novel fluorine-containing organic cation (4FH-PPH^+^) into the 2D RP HP material whose *E*_g_ is small and unlike those of all of the fluorinated 2D RP perovskite materials. In other words, the *E*_g_ = 2.22 eV of this fluorinated (4FH-PPH)_2_PbI_4_ is much smaller than those of other (*n* = 1) kinds of fluorinated 2D HPs. Additionally, the reported 2D HP PD with an excellent photoresponsivity of 502 mA W^–1^ under 450 nm (and 463 mA W^–1^ under 520 nm) laser illumination at a bias voltage of 20 V demonstrates a remarkable stability of up to 49 days without encapsulation. Additionally, the 2D HP PD shows impressive response/recovery times of 42/46 and 43/49 ms and outstanding photodetectivities of 1.57 × 10^13^ and 1.45 × 10^13^ Jones under 450 and 520 nm, respectively. When compared with most of the previously reported 2D HP PDs, this fluorinated device shows outstanding 2D HP PD properties in terms of photoresponsivity, response and recovery time, and stability. These results strongly demonstrate the potential of the fluorinated short-chained (4FH-PPH)_2_PbI_4_ HP as a promising candidate for the next-generation fast, high-performance, cost-effective, highly stable, optical, and/or optoelectronic devices.

## 2 Experimental Section

### 2.1 Chemistry

**Figure 1:**
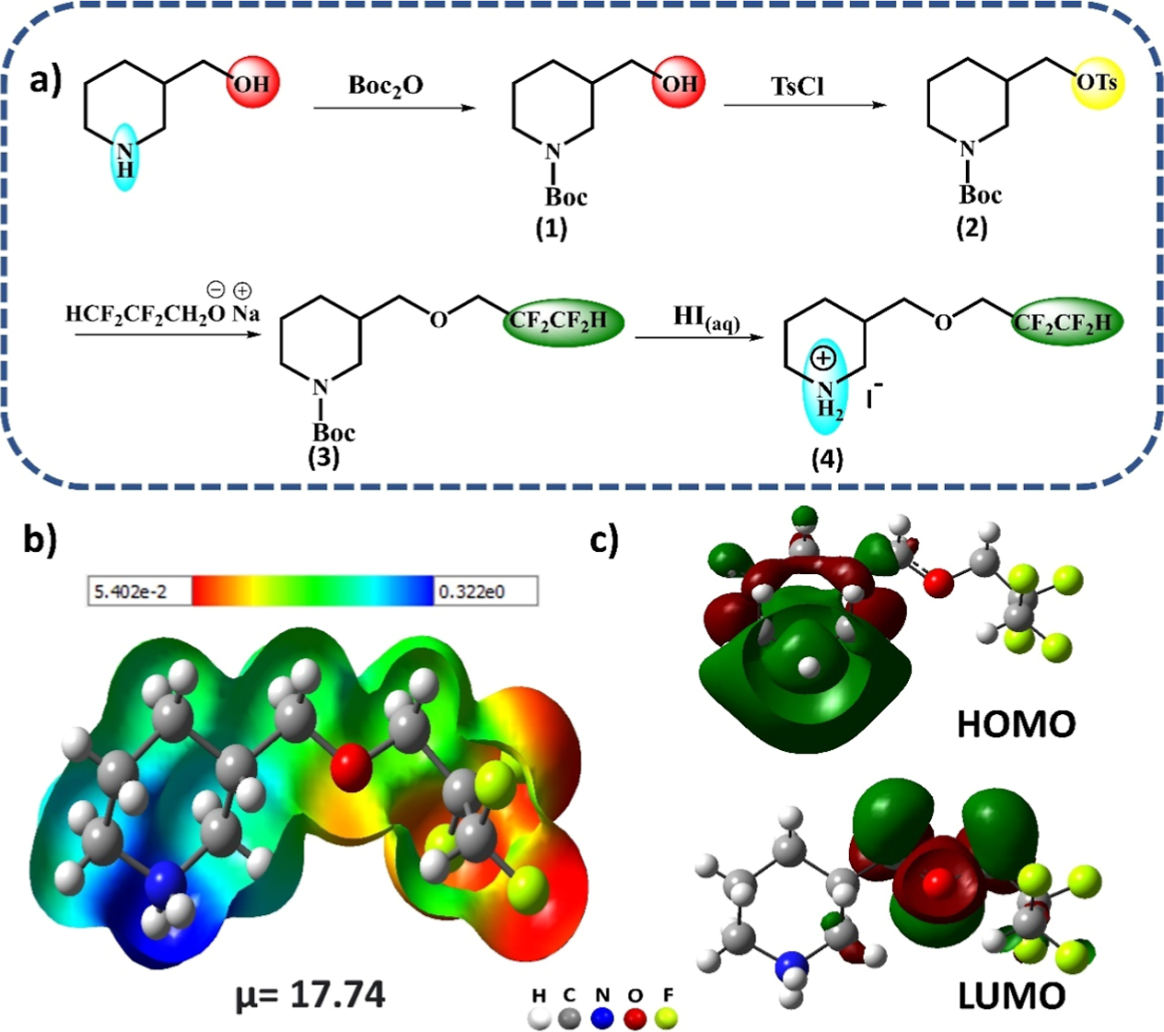
(a) Synthetic scheme of the fluorinated 4FH-PPH^+^ organic cation preparation. (b) ESP plot with a positive charge on the N atom. (c) Electron density in the highest occupied molecular orbital (HOMO) and lowest unoccupied molecular orbital (LUMO) diagrams of 4FH-PPH^+^.

The starting materials, reagents, and solvents employed were commercially available and were purchased from either Aldrich (Taipei, Taiwan) or SynQuest (FL, USA) and used as received. The purity of all the intermediates and products (compounds 1–4 shown in Figure 1a) was monitored by using gas chromatography/mass by utilizing an Agilent 6890 series gas chromatograph and series 5973 mass selective detector. Ligand synthesis was monitored with an HP 6890 GC using a 30 m HP–1 capillary column with a 0.25 μm stationary phase film thickness. The flow rate was 1 mL/min, and splitless injection was used. TLC was done using Merck Kieselgel 60 F254 aluminum plates. Infrared spectra were obtained by using a PerkinElmer RX I FT–IR spectrophotometer. NMR spectra were recorded on Bruker AM 600, 400, and 300 spectrometers using 5 mm o.d. sample tubes. D_2_O, CDCl_3_, and DMSO-*d*_6_ were the references for both ^1^H and ^13^C NMR spectra, while refrigerant–11 (CFCl_3_) was the reference for ^19^F NMR spectra. The coupling constants *J* are given in hertz (Hz).

### 2.2 Synthesis of Fluorinated (4FH-PPHI) and Nonfluorinated (5H-PPHI) Iodide Salts

The fluorinated organic salt (spacer), 3-((2,2,3,3-tetrafluoropropoxy)methyl)piperidinium iodide (3-**HCF**_**2**_CF_2_CH_2_OCH_2_-PP-**NH**_**2**_^+^), abbreviated as (4FH-PPHI), and the nonfluorinated organic salt, 3-(propoxymethyl)piperidinium iodide (3-**HCH**_**2**_CH_2_CH_2_OCH_2_-PP-**NH**_**2**_^+^), abbreviated as (5H-PPHI), have been successfully prepared and fully characterized. The detailed synthetic procedures and analytical data of all the related compounds have been shown in the Supporting Information.

### 2.3 Preparation of (4FH-PPH)_2_PbI_4_ 2D Perovskite Precursor Solution

We prepared the (4FH-PPH)_2_PbI_4_ precursor solution. We have used a 2:1 stoichiometric ratio of PbI_2_ (92.2 mg, 0.2 mmol) and 4FH-PPHI (142.8 mg, 0.4 mmol), which have been dissolved in an *N*,*N*-dimethylformamide (DMF) solvent under ambient condition. The obtained solution has been stirred at 60 °C for 2 h and allowed to cool down to room temperature before the usage.

### 2.4 Characterization of (4FH-PPH)_2_PbI_4_ Perovskite

The crystallographic structure information on (4FH-PPH)_2_PbI_4_ was characterized using a Rigaku XtaLAB Synergy DW single crystal diffractometer equipped with a HyPix-Arc 150° curved Hybrid Photon Counting X-ray detector and Micro Max-007 HF microfocus rotating anode with dual wavelength (Cu and Mo). The phases of the HP film were measured using a PAN analytical Empyrean powder X-ray diffractometer with Cu Kα source radiation and equipped with a PIXcel^1D^ detector. The diffraction patterns were collected in the 2θ range of 10 to 80° with a step size of 0.01°. The X-ray photoelectron spectroscopy (XPS) measurements were recorded using a Thermo Scientific Multi-Lab 2000 instrument. The high-resolution field emission scanning electron microscopy (FESEM) images were taken by a Hitachi-SU8100 instrument. The atomic force microscopy (AFM) image was taken by an FSM, Nano view 1000 instrument. The high-resolution transmission electron microscopy (HR-TEM) images were taken using a Hitachi-FEI Tecnai G2 instrument, operated at 200 kV to record the lattice fringe images. The absorption spectrum of the (4FH-PPH)_2_PbI_4_ film was recorded with a JASCO V670 spectrophotometer. The photoluminescence (PL) spectrum (excitation at 400 nm) was measured with a JASCO FP-8500 spectrofluorometer. Time-resolved photoluminescence (TRPL) was obtained from an PicoQuant PicoHarp 300 instrument with Picoquant LDH series picosecond pulse laser diode (405 nm, 19.1 μW). The water contact angle measurement of the (4FH-PPH)_2_PbI_4_ film was recorded using a theta optical tensiometer. Additionally, the solid (ATR) FT-IR data of the perovskite material are also taken. FT-IR υ (cm^–1^): 3098, 3049, 3046 (υ_pp-N-H_), 2951, 2934, 2893 (υ_pp-CH_2__), 1549 (υ_pp-N-H-bend_), 1138, 1096 (υ_CF_2__). [Note: the stability of the fluorinated perovskite material can usually be double-checked by monitoring its FT-IR spectrum (Figure S5).]

### 2.5 Device Fabrication and Measurements

The SiO_2_/Si substrate was cleaned in ethanol, acetone, and deionized water in series by using an ultrasonic bath. A photolithography procedure was performed to define the interdigitated electrode patterns for the photodetector (PD) device. The Ti/Au (5/55 nm) metal electrodes were deposited with electron-beam evaporation. The interdigital electrode measures 500 μm (single pad) in both length and width. For the interdigital electrode, the channel dimensions are 6 μm in length and 153 μm in width. Moreover, the active area of the device is found to be 1.335 × 10^–5^ mm^2^. Then, 50 μL of the prepared (4FH-PPH)_2_PbI_4_ HP precursor solution was deposited onto the surface of the SiO_2_/Si substrate by a one-step spin coating technique with a spin rate of 2000 rpm for 30 s. During the process, the film turned from pale yellow to orange in a few seconds as the solvent evaporated. After spin coating, the prepared film was quickly removed from the spin coater and the film had annealed on the top of a hot plate at 60 °C for 15 min. Finally, the electrical and optoelectronic properties of the (4FH-PPH)_2_PbI_4_ fluorinated short-chained HP PD were characterized using a semiconductor analyzer (Keithley 2612) under dark and laser light illumination in ambient conditions. Power-tunable lasers with wavelengths of 450 and 520 nm were used to characterize the photoresponses and other optoelectronic properties of the devices. The light powers were measured by a power meter (LP10, SANWA, Japan).

## 3 Results and Discussion

### 3.1 Special Features and Computational Studies of the Fluorinated Organic Cation

Fluorination has emerged as a promising molecular design strategy to enhance the moisture stability, crystal quality, and overall performance of organic solar cells, light-emitting diodes (OLEDs), and photodetectors (PDs). For example, replacing hydrogen with fluorine on the benzene ring of phenethylammonium (PEA) cations leads to significant improvements in the stability and performance of various optoelectronic devices.^40^ Based on these findings, we have successfully synthesized the fluorine-containing piperidinium iodide salt, 3-((2,2,3,3-tetrafluoropropoxy)methyl)piperidinium iodide (abbreviated as 4FH-PPHI). After the purification, the protecting groups were cleaved, and the resulting amine was protonated with hydroiodic acid to yield compound (4) shown in Figure 1a. The detailed synthetic procedures and their corresponding analytical data are presented in the Supporting Information and briefly discussed in this Experimental Section. Furthermore, 4FH-PPH^+^ was investigated using density functional theory to calculate various properties, such as electric dipole moments, electrostatic potential surface (ESP), the HOMO, and the LUMO. The ESP plot and molecular orbitals have been calculated by using Gaussian16 with the B3LYP/6-311G(d,p) level of theory with an iso-surface value of 0.01 au. As shown in Figure S1, the fluorinated 4FH-PPH^+^ organic spacer has a larger dipole moment of 17.74 D (Debye) than that (being 9.54 D) of the nonfluorinated 5H-PPH^+^ molecule. This increased dipole moment of the fluorinated HP can certainly help in separating charge, regulating the surrounding dielectric environment, and reducing the dielectric confinement effect in 2D HPs such that the charge transport of the reported PD device can be improved.^31,41^ The ESP calculations revealed the accumulation of a positive charge (dark blue) on the nitrogen atom in piperidinium (PPH^+^) shown in Figure 1b. Moreover, as shown in Figure 1c, the HOMO in organic PPH^+^ was found to be distributed on the nitrogen atom, whose positive charge is intensified due to the strong electron-withdrawing ability of the fluorinated side-chain.

The high electronegativity of the fluorine atom could withdraw electrons and induce a more positive charge on NH_3_^+^, resulting in a stronger Coulomb force between 4FH-PPH^+^ and the [PbI_6_]^4–^ octahedra. This stronger interaction could enhance the stability of the perovskite crystal structure. To analyze the interaction between the fluorinated cationic spacer and [PbI_6_]^4–^ octahedra, as well as to verify the element distribution and chemical states on the surface of the 2D HP film (see Figure S2), we have performed XPS measurements. The XPS spectra have revealed that the chemical states of all of the elements present in the fluorinated 2D HP are consistent with its ideal chemical formula of the fluorinated (4FH-PPH)_2_PbI_4_ HP. The complete discussions of Figure S2 are provided in the Supporting Information. As shown in Figure S2c, the fluorinated HP shows two characteristic peaks appearing at 142.5 and 137.6 eV for Pb 4f (of the Pb 4f core levels). As shown in Figure S2c, these two peaks are then assigned to Pb 4f_5/2_ and Pb 4f_7/2_, respectively. Similarly, as shown in Figure S2d, two prominent peaks for the I 3d core levels are located at 629.7 and 618.3 eV, which correspond to I 3d_3/2_ and I 3d_5/2_, respectively. The observed binding energies of Pb 4f and I 3d are slightly shifted to lower energies.^42^ The slight shifts of the Pb 4f and I 3d peaks toward the lower binding energies can be attributed to the electrostatic interactions between the fluorinated organic cationic spacer and the [PbI_6_]^4–^ octahedra.^31,41^

### 3.2 Crystal Growth and X-ray Structural Analysis of the Fluorinated Perovskite Compound

**Figure 2:**
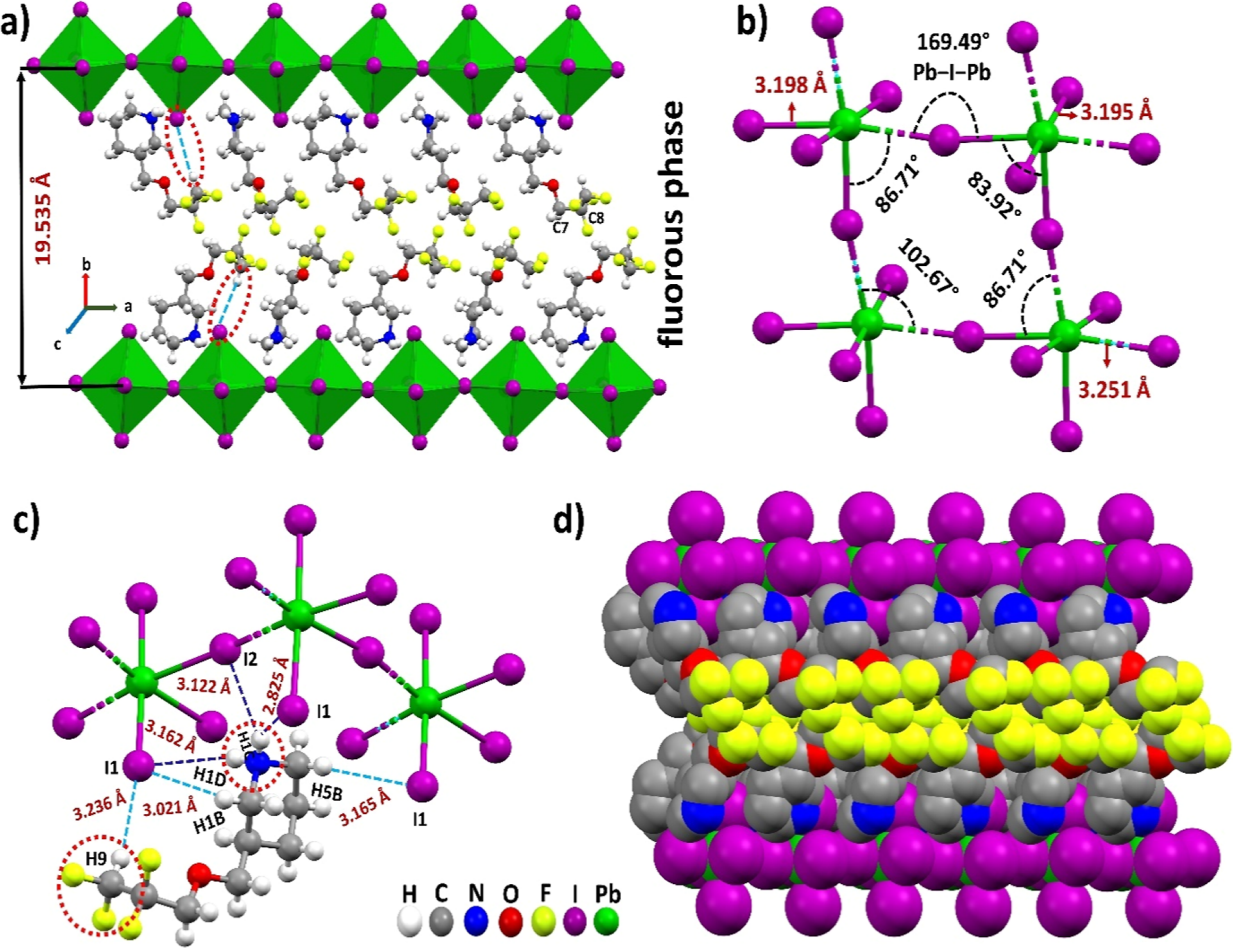
Single-crystal structure of (4FH-PPH)_2_PbI_4_ showing (a) 2D layered packing with the unique fluorous phase [also see (d)], (b) the core unit of Pb(II) with the bond lengths and angles, (c) N–H···I hydrogen bonding and C–H···I improper hydrogen bonding interaction between 4FH-PPH^+^ cation and inorganic sheets, and (d) packing mode with the space-filling view of the inorganic layer and both the organic and fluorous layers from the organic cations.

The (4FH-PPH)_2_PbI_4_ HP crystals were synthesized according to the previously reported antisolvent diffusion method.^43^ A vial containing the 2:1 stoichiometric ratio of the 0.1 mol L^–1^ solution of 4FHPPHI and PbI_2_ in γ-butyrolactone (GBL) was put in a larger sealed vial with the antisolvent inside as shown in Figure S3a. The fluorinated (4FH-PPH)_2_PbI_4_ HP was left for slow crystallization when the antisolvent (dichloromethane) was slowly diffused into the precursor solution. After a week, the millimeter-sized, transparent, colorless, and well-shaped 2D (4FH-PPH)_2_PbI_4_ HP crystals started to grow in the small vial. Figure S3b shows an optically microscopic image of the (4FH-PPH)_2_PbI_4_ crystal. The structure of the layered perovskite crystal of (4FH-PPH)_2_PbI_4_ HP was investigated by using single-crystal X-ray diffraction. The (4FH-PPH)_2_PbI_4_ HP single crystal shows the layered perovskite structure consisting of the infinite corner sharing the [PbI_6_]^4–^ octahedra and layers of 4FH-PPH^+^ molecules occupying the space between sheets to form the 2D HP structure as shown in Table 1 (also see the Supporting Information) and Figure 2a. The detailed crystallographic data and structural refinements are listed in Table 1 (also see Tables S1–S3 in the Supporting Information). The compound of (4FH-PPH)_2_PbI_4_ HP crystallizes in the orthorhombic *Aba*2 space group with the asymmetric unit containing one lead(II) atom, two iodine atoms, and one 4FH-PPH^+^ cation. The arrangement of the lead atoms alternates from layer to layer, resulting in a staggered arrangement of the two immediately adjacent layers with an interlayer distance of 19.535 Å. This kind of arrangement is likely due to the 5-atom long poly fluorinated tail. Due to the layer-by-layer segregation of the fluorinated organic and the inorganic components in the hybrid perovskites, the fluorinated organic cation, which has four F atoms in each side-chain forming the well-ordered fluorous layer, plays an important role in incorporating the stability into the polyfluorinated hybrid perovskites. The degree of stabilization depends on the incorporation of the fluorous layer and the compactness of its packing as well as its chemical stability.

**Table 1:** X-ray Crystallographic Data and Structure Refinement for (4FH-PPH)_2_PbI_4_ HP

X-ray diffraction of (4FH-PPH)_2_PbI_4_	
CCDC number	2262737
Crystal Data	
chemical formula	C_36_H_64_F_16_N_4_O_4_Pb_2_I_8_
*M*_r_	2350.51
crystal system, space group	orthorhombic, *Aba*2
temperature (K)	100
*a*, *b*, *c* (Å) α, β, γ (deg)	9.3378 (3), 37.9387 (12), 8.8182 (2) 90, 90, 90
*V* (Å^3^)	3123.97 (16)
*Z*	2
radiation type	Mo Kα
m (mm^–1^)	9.42
crystal size (mm)	0.2 × 0.12 × 0.02
Data Collection	
diffractometer	XtaLAB Synergy R, DW system, HyPix-Arc 150
absorption correction	Multiscan^a^
*T*_min_, *T*_max_	0.268, 0.828
no. of measured, independent, and observed [*I* > 2 s(*I*)] reflections	13,597, 3577, 3411
*R*_int_	0.027
(sin *q*/*l*)_max_ (Å^–1^)	0.650
Refinement	
*R*[*F*^2^ > 2 s(*F*^2^)], *wR*(*F*^2^), *S*	0.022, 0.053, 1.030
no. of reflections	3577
no. of parameters	183
no. of restraints	25
H atom treatment	H atom parameters constrained
*D*ρ_max_, *D*ρ_min_(e Å^–3^)	0.75, −0.50

a *CrysAlis PRO* 1.171.42.51a (Rigaku Oxford Diffraction, 2022).

**Figure 3:**
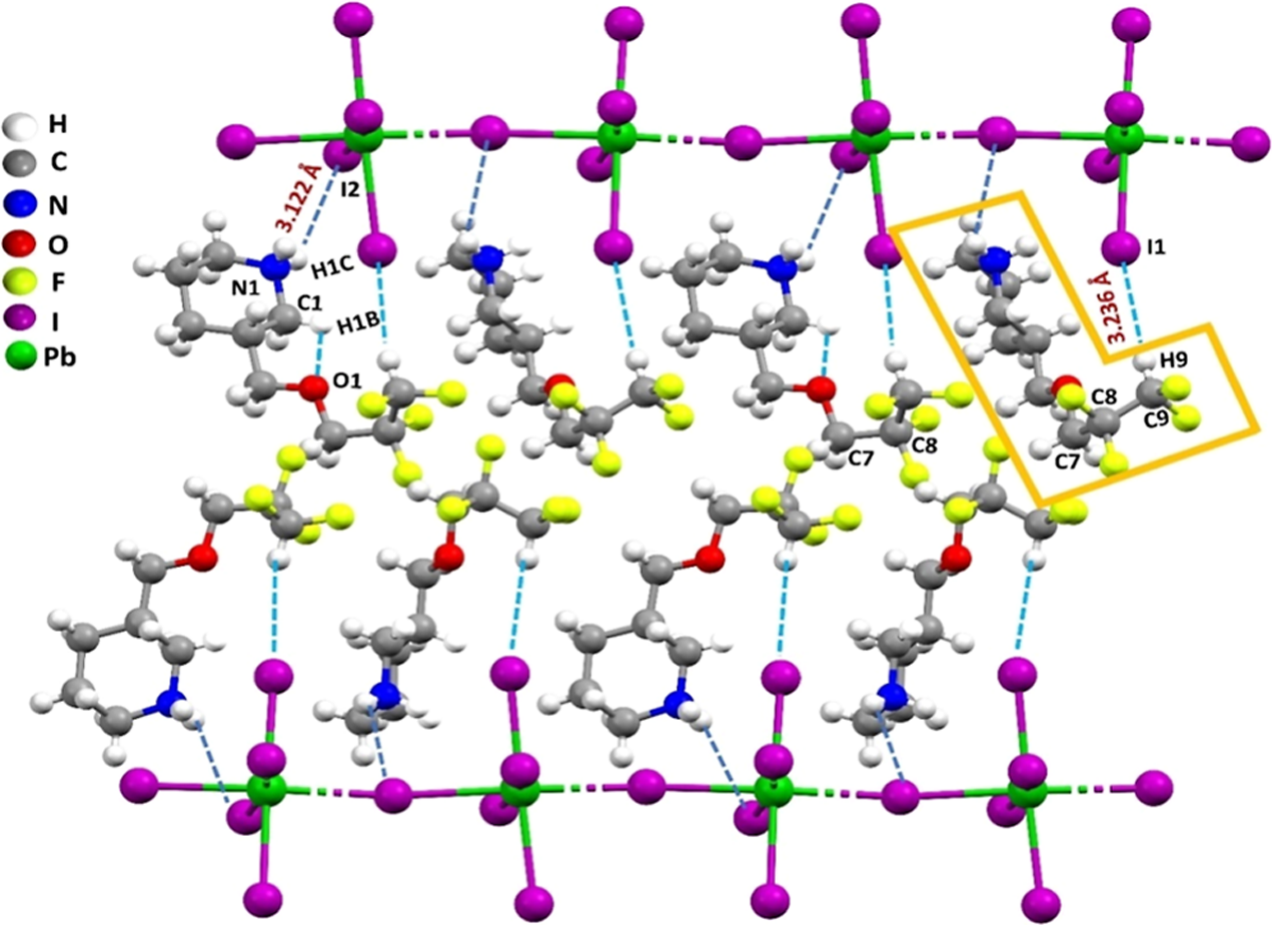
Single-crystal structure of (4FH-PPH)_2_PbI_4_ showing N–H···I hydrogen bonding (dashed dark blue lines) and the terminal HCF_2_ end group forms the hydrophobic C–H···I (dashed light blue lines) blue-shifting HB that leads to forming the L-shaped (a hook shape) arrangements (in highlighted orange loop in the upper right corner).

Interestingly, the presence of fluorine atoms in the fluorinated organic moiety has been found to result in the formation of a unique fluorous phase in the perovskite crystal structure. This fluorous phase can greatly enhance the fluorophobicity of the fluorinated 2D short-chained (4FH-PPH)_2_PbI_4_ HP with moisture tolerance, making it more stable in air at room temperature. Figure 2b displays the packing structure of the fluorinated short-chained (4FH-PPH)_2_PbI_4_ HP that reveals the presence of the distorted [PbI_6_]^4–^ octahedra within the inorganic layers. Each Pb ion coordinates with the six I^–^ ions, generating an octahedral geometry. In this geometry, the Pb–I distances range from 3.195 to 3.250 Å and the Pb–Pb distance is 6.422 Å. Additionally, the I–Pb–I bond angles vary from 83.92 to 177.97° and the Pb–I_eq_–Pb angle is 169.49° (being 10.5° smaller due to the organic spacer; eq means equatorial), which implies a slight distortion from the ideal 180° angle. To the best of our knowledge, this is the smallest distortion observed in fluorinated 2D RP HP so far. This distortion of the [PbI_6_]^4–^ octahedra can be attributed to the fluorophobicity character of the flexible fluorous phase, which appears to orderly compress the organic phase into the inorganic pocket and eliminate the possibility of a greater structural distortion in the inorganic [PbI_6_]^4–^ planes. This quite small angle change (∼10°), which is very different from the significantly deviated angle of all other fluorinated 2D RP HP materials, is likely due to a special difunctional linker of the organic spacer, [^+^H_2_N–C_5_H_9_–CH_2_OCH_2_CF_2_(CF_2_H)] (see compound 4 in the Supporting Information), and its C–O–C ethereal linkage, so both ends (of ^+^H_2_N and CF_2_–H) of a spacer can adapt the **L** shape (a hook shape) in a way that the fluorous segment can turn almost 90° via a flexible C–O–C linkage and then the C(7)–C(8) single bond is almost parallel with the inorganic layer (shown in Figures 2a and 3). Both ends of (^+^H_2_N and CF_2_–H) HB donors then form the two specific HBs with two different iodides from the orthogonal I–Pb–I segment in the inorganic [PbI_6_]^4–^ octahedra from the 2D RP HP materials (shown in Figure 3). In other words, the fluorous layer is then horizontally oriented (see Figure 2d) such that the inorganic layer, organic layer, and fluorous layer are almost parallel with one another. It is shown for the first time that the bifunctional organic spacer linker can use its two terminal ends to form both the normal HB and hydrophobic blue-shifting HB with the same inorganic layer. It is also the first example that the H–CF_2_ terminal group of an organic spacer (as a difunctional linker) can be used to form the hydrophobic (blue-shifting) HB to weakly bond to the inorganic segment.

Interestingly, this smaller distortion of inorganic [PbI_6_]^4–^ octahedra resulting from the special fluorinated 4FH-PPH^+^ organic spacer can allow us to tune the energy bandgap of the (4FH-PPH)_2_PbI_4_ perovskite in a way that the *E*_g_ of fluorinated (4FH-PPH)_2_PbI_4_ is 2.22 eV, which is much smaller than those of other (*n* = 1) kinds of fluorinated 2D HPs (also see entries 13, 14 in Table S4). The selected crystallographic data (such as Pb–I bond lengths, I–Pb–I bond angles, and Pb–I–Pb bond angles) are listed in Tables S1 and S2. As shown in Figure 2c, the secondary amine group (NH) in a piperidinium forms the strong N1–H1C···I1 (2.825 Å), weak N1–H1C···I2 (3.122 Å), N1–H1D···I1 (3.162 Å) hydrogen bond (HB) interaction, and weak C–H···I improper HB interaction via C1–H1B···I1 (3.021 Å), C9–H9···I1 (3.236 Å), and C5–H5B···I1 (3.165 Å) with the [PbI_6_]^4–^ octahedra, respectively (see Table S3). Additionally, the 4FH-PPH^+^ organic cation (shown in Figure S4) is interconnected via C6–H6A···F3 (2.482 Å), C6–H6B···F2 (2.513 Å), respectively. The combinations of the noncovalent interactions (NCIs), which include two improper HBs of C1–H1B···O1 (2439 Å) and C6–H6A···H7B–C7 (2413 Å)^39^ and the C8–F2···F4–C9 (2.809 Å) halogen bond^37^ interaction,^38^ can not only strengthen the cohesive forces inside the organic molecules, but also effectively cover the voids present in the crystal packing shown in Figure 2d. As a result, a strong water-repellent, compact fluorous layer was formed. Additionally, the unusual stability of this fluorinated 2D RP-type HP material can also be affirmed by the good water contact angle and the presence of blue-shifting HB after being kept at the room temperature for 49 days as shown in Figure S5 (in the Supporting Information).

### 3.3 Material Characterization and Optical Properties

**Figure 4:**
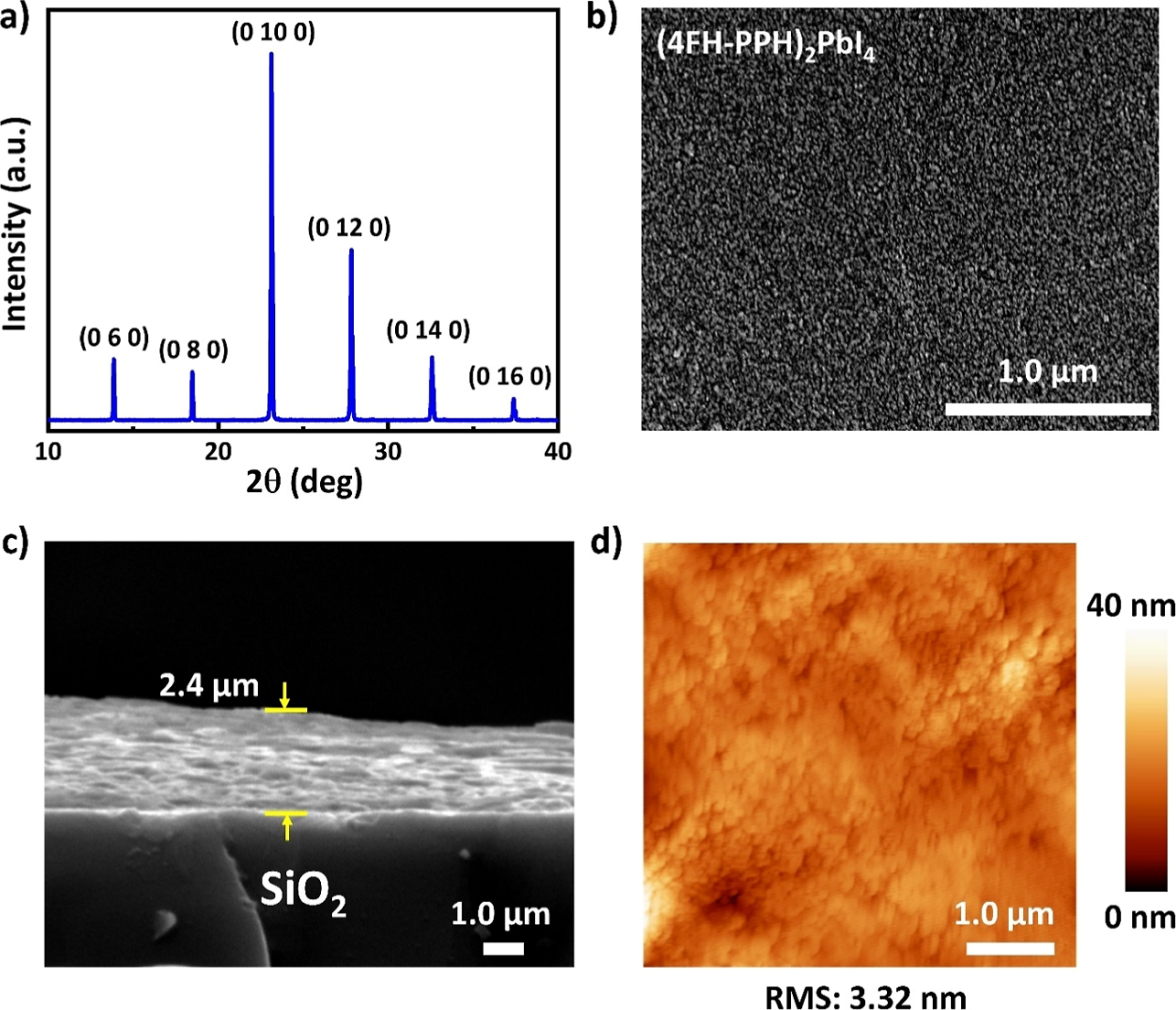
Structural and morphological characterization of the fluorinated short-chained (4FH-PPH)_2_PbI_4_ HP film showing a (a) PXRD pattern with very sharp peaks, (b) top-view high-resolution FESEM image with a scale bar of 0.5 μm, (c) cross-sectional FESEM image and, (d) its AFM image with a roughness of 3.32 nm.

The fluorinated short-chained (4FH-PPH)_2_PbI_4_ HP film was prepared by spin-coating the precursor solution of (4FH-PPH)_2_PbI_4_ in DMF onto a glass (or SiO_2_/Si) substrate at 2000 rpm for 30 s followed by annealing on the top of a hot plate at 60 °C for 15 min in the air. The uniform orange fluorinated short-chain (4FH-PPH)_2_PbI_4_ HP can be readily formed. It is worth noting that the color of the prepared perovskite film undergoes no color change even when the film has been exposed to air for 49 days (see insets in Figure S5a), indicating the outstanding air stability of the fluorinated (4FH-PPH)_2_PbI_4_ thin film. This has been further confirmed by periodically analyzing the structure of the film by powder X-ray diffraction (PXRD). As shown in Figure 4a, the sharp, intense, and well-defined peaks that repeat periodically correspond to the (0 *k* 0) (*k* = 2*n*) series of reflections planes in the fluorinated (4FH-PPH)_2_PbI_4_ layered structure. As expected, the PXRD peaks remain unchanged, and no impurity peaks have been observed even after 4 months in air at ambient temperature (as shown in Figure S5a). Furthermore, we have affirmed the stability of the fluorinated perovskite material by monitoring the FT-IR spectrum. It has been found that the blue-shifting HB frequencies (as fingerprints) for terminal sp^3^ C–H stretching after 4 months are still present (as shown in the Figure S5b inset). To demonstrate the impact of the fluorinated cations on crystallinity, we have synthesized a novel nonfluorinated piperidinium (PP) cation to construct an RP-type *n* = 1 2D HP. We have investigated the crystallinity of both fluorinated [(4FH-PPH)_2_PbI_4_] and nonfluorinated [(5H-PPH)_2_PbI_4_] HP films with a short-chain using PXRD. [Note: 4FH stands for a fluorinated side-chain of HCF_2_CF_2_, while the 5H stands for a nonfluorinated chain of HCH_2_CH_2_ (or CH_3_CH_2_)]. In contrast, the (5H-PPH)_2_PbI_4_ HP film, prepared by using nonfluorinated piperidinium, hardly shows the crystalline form in the PXRD pattern and has found to be decomposed readily in air as shown in Figure S6b. Thus, these results are able to affirm that the fluorinated short-chained HP can contribute to enhance the crystallinity of the 2D HPs when compared with its nonfluorinated short-chained analogue.

It is also known that the humidity tolerance of the 2D perovskite can be further enhanced by the incorporation of a fluorinated short-chain. To demonstrate this effect on the hydrophobicity of the surface, water contact angle measurement was carried out on the fluorinated short-chained (4FH-PPH)_2_PbI_4_ HP film. As shown in Figure S7, the average contact angle of the fluorinated (4FH-PPH)_2_PbI_4_ HP film was found to be 86.5°, indicating that the fluorinated 2D short-chained perovskite film was less affected by humidity. Therefore, the moisture-resistant property of this material can be mainly attributed to the fluorophobicity of the fluorous phase in the crystal structure. The greatly enhanced order and packing of organic layers, hydrophobicity, fluorophobicity, high orientation, and densely packed nature of the fluorinated (4FH-PPH)_2_PbI_4_ film can effectively prevent the direct contact of adventitious water from entering the perovskite component, leading to significantly enhanced stability.^44,45^

In addition, the morphological properties of the fluorinated short-chained (4FH-PPH)_2_PbI_4_ HP film have been characterized by high-resolution FESEM and AFM as shown in Figure 4. Figure 4b shows the top-view high-resolution FESEM image of the fluorinated (4FH-PPH)_2_PbI_4_ HP film. This fluorinated HP film certainly shows the homogeneous, smooth, and pinhole-free surface morphology with excellent surface coverage, suggesting a uniform distribution leading to the reduction in charge carrier recombination, which can be responsible for the high device performance. Thus, we observed sharp, intense, and well-defined diffraction peaks in Figure 4a. Additionally, Figure 4c shows that the thickness of the fluorinated (4FH-PPH)_2_PbI_4_ HP film was measured using a cross-sectional FESEM image. The average thickness of the film has been measured to be ∼2.4 μm. The smooth and thin surface help to have the strong interface contact between the two layers, resulting in the fluorinated HP photodetector with good performance. Furthermore, the AFM image shown in Figure 4d demonstrates that the fluorinated (4FH-PPH)_2_PbI_4_ HP film was smooth with a roughness of 3.32 nm by a root-mean-square (RMS). The AFM morphology of 2D (4FH-PPH)_2_PbI_4_ HP is consistent with the surface morphology presented in the top-view FESEM image (see Figure 4b). This is mainly attributed to the presence of the fluorinated short-chain in the 2D RP perovskite system.^46^ In addition, we have also obtained a FESEM image of the fluorinated (4FH-PPH)_2_PbI_4_ HP powder shown in Figure S8 in the Supporting Information.

**Figure 5:**
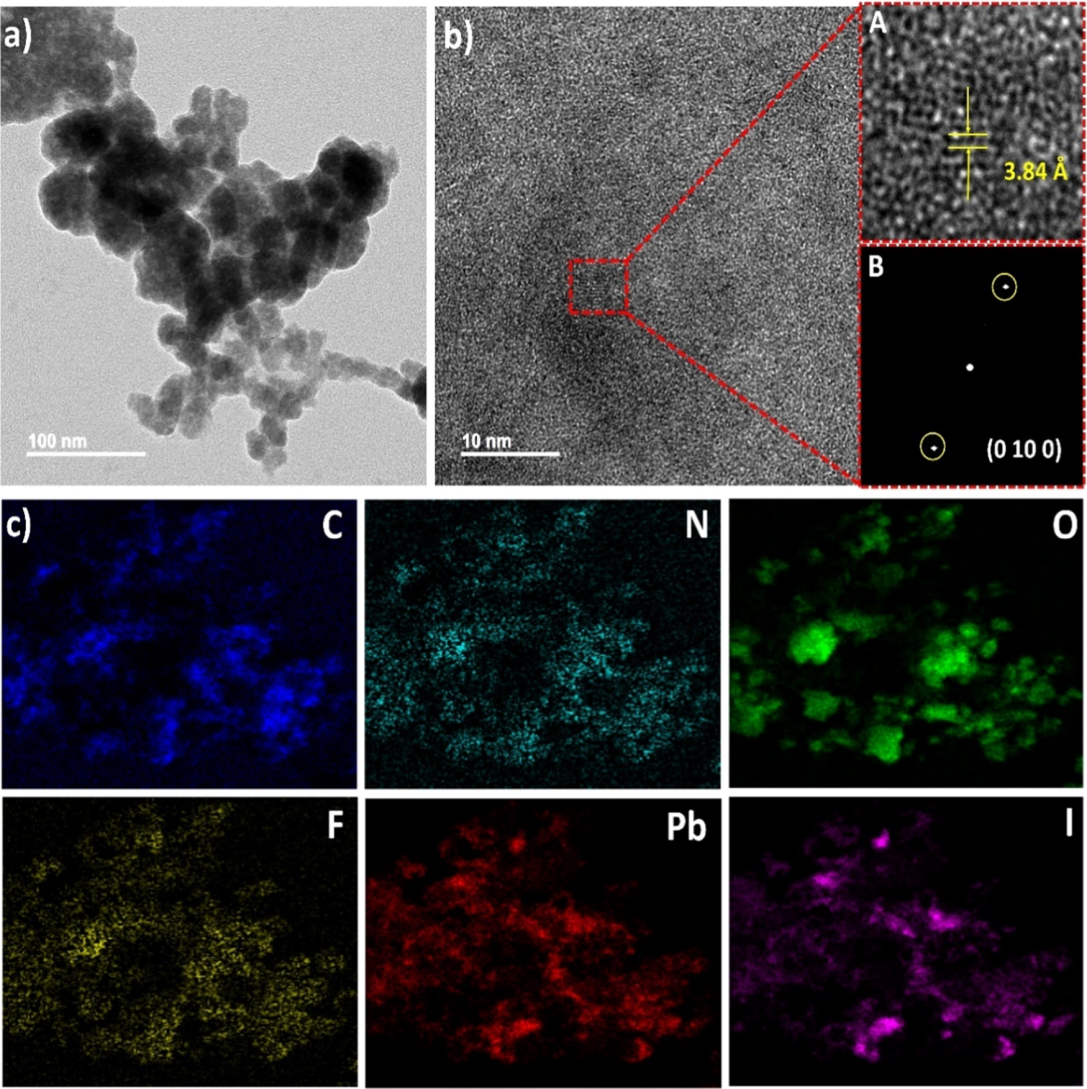
(a,b) HR-TEM images and FFT patterns of fluorinated short-chained (4FH-PPH)_2_PbI_4_ HP and (c) EDS-mapping for the fluorinated short-chained (4FH-PPH)_2_PbI_4_ HP. Note: insets A and B of (b) show an enlarged HR-TEM image and a corresponding FFT pattern of the fluorinated side-chained (4FH-PPH)_2_PbI_4_ HP, respectively.

The HR-TEM, fast Fourier transform (FFT), and energy-dispersive X-ray spectroscopy (EDX or EDS) study of the both fluorinated [(4FH-PPH)_2_PbI_4_] and nonfluorinated [(5H-PPH)_2_PbI_4_] short-chained HP films were further performed and are shown in Figure 5 (also see Figures S9–S11, respectively). Figure 5a shows that the morphology of the fluorinated short-chained (4FH-PPH)_2_PbI_4_ HP is homogeneous. Figure 5b shows the HR-TEM image of fluorinated (4FH-PPH)_2_PbI_4_ HP, which has allowed us to calculate the *d*-spacing value. The primary method to determine the *d*-spacing was to perform an FFT on a crystalline region of this deposited material (inset B). As shown in Figure 5b (inset A), for the fluorinated (4FH-PPH)_2_PbI_4_ HP, an interplanar spacing of 3.84 Å in the region marked with a red rectangular box (see Figure 5b) is well matched with the (0 10 0) diffraction data of the *n* = 1 phase. Additionally, Figure 5b (inset B) shows that the interplanar spacing (=3.84 Å) calculated by FFT also matches with the corresponding XRD results. These results have confirmed that the fluorinated (4FH-PPH)_2_PbI_4_ HP indeed exhibits the good crystalline nature. Furthermore, Figure S9 shows the HR-TEM image (with a 2 μm scale bar) to highlight the selected area for EDS mapping of the fluorinated (4FH-PPH)_2_PbI_4_ HP. As shown in Figure 5c, the EDS-mapping of the fluorinated (4FH-PPH)_2_PbI_4_ HP reveals a uniform distribution of all the elements (C, N, O, F, Pb, and I). The EDS spectrum of the fluorinated (4FH-PPH)_2_PbI_4_ HP film affirms the presence of all the elements shown in Figure S10 (see the Supporting Information). The atomic ratios of the elements C, N, O, F, Pb, and I have been found to be 63.3, 5.2, 5.0, 17.6, 2.1, and 7.0%, respectively. These ratio values are close to those of its ideal molecular formula of the fluorinated short-chained (4FH-PPH)_2_PbI_4_ HP to affirm a good distribution of all the elements. These results have again affirmed that the fluorinated short-chained (4FH-PPH)_2_PbI_4_ HP is able to exhibit a uniform distribution without the phase segregation.

In contrast, the (5H-PPH)_2_PbI_4_ perovskite film, prepared using a normal (nonfluorinated) piperidinium, does not show the crystalline form in the PXRD pattern and has readily decomposed in air, as shown in Figure S11. As a result, during HR-TEM investigation, the nonfluorinated HP, (5H-PPH)_2_PbI_4_, has decomposed (shown in Figure S11a). However, as shown in Figure S11b, we have noticed several crystalline areas in its HR-TEM image, which is presumably due to the presence of the decomposed side-product, PbI_2_. The HR-TEM discussions of the nonfluorinated (5H-PPH)_2_PbI_4_ HP have also been provided in the Supporting Information. Thus, these results are able to affirm that the fluorinated side-chained HP can contribute to enhance the crystallinity of the 2D perovskites when compared with its nonfluorinated short-chained analogue.

**Figure 6:**
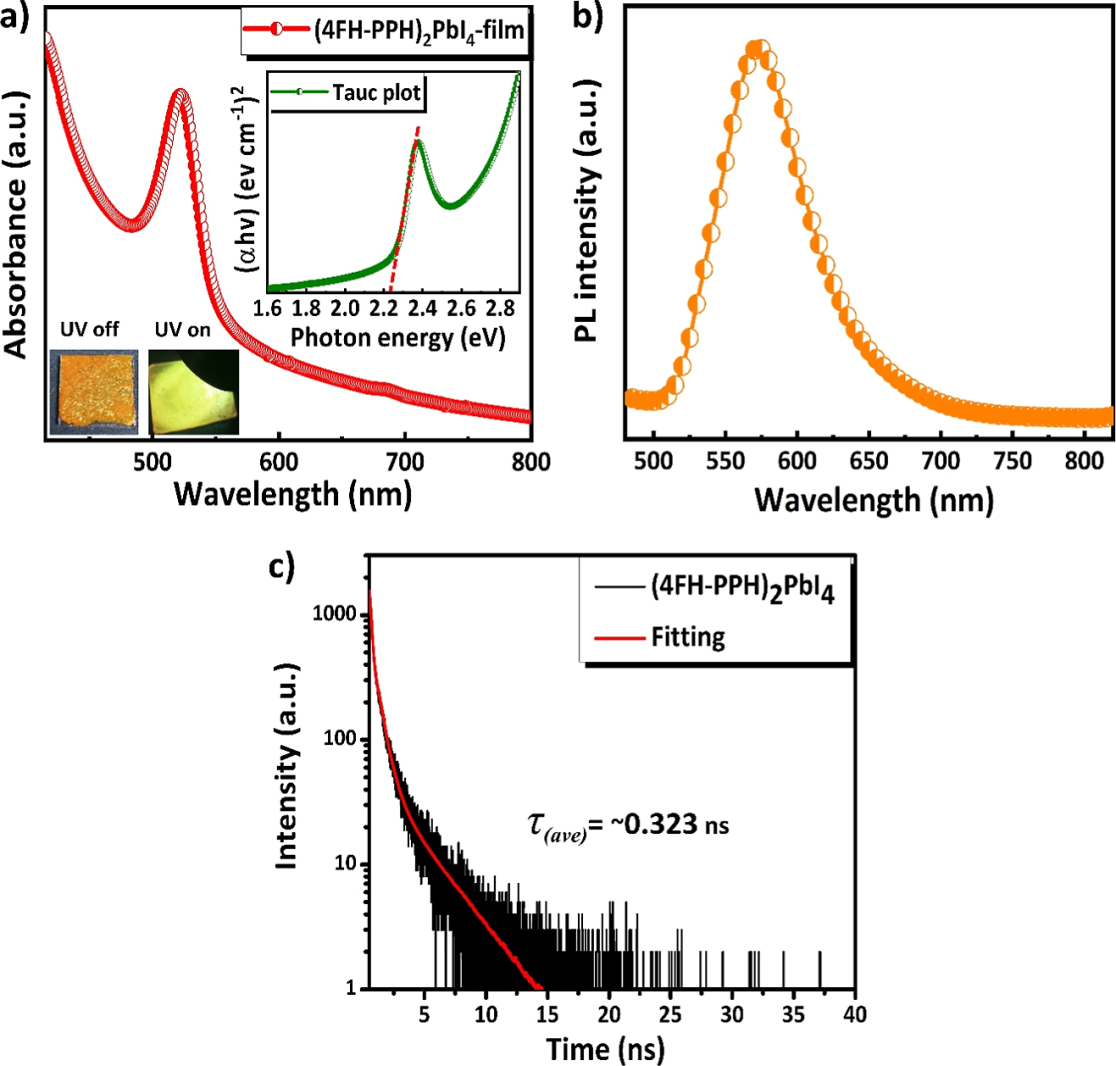
Optical characterization of the fluorinated short-chained (4FH-PPH)_2_PbI_4_ HP film showing the (a) UV–vis absorption spectrum, (b) PL spectrum, and (c) TRPL spectrum.

In order to explore the impact of the fluorous phase on optoelectronic properties of this material, ultraviolet–visible (UV–vis) absorption and PL spectra of the film have been recorded and shown in Figure 6a,b, respectively. It has been found that the fluorinated (4FH-PPH)_2_PbI_4_ HP film exhibits a remarkably sharp absorption edge at ∼540 nm. The optical bandgap (*E*_g_) of 2.22 eV has also been determined according to the Tauc plot shown in Figure 6a. To the best of our knowledge, the observed *E*_g_ = 2.22 eV of the fluorous short-chained (4FH-PPH)_2_PbI_4_ RP HP is the lowest value among all other fluorinated 2D perovskites reported so far (see Table S4). The low *E*_g_ of 2.22 eV in the fluorous (4FH-PPH)_2_PbI_4_ RP HP can be attributed to the formation of a unique fluorous phase in the HP structure and a hooked type of weak interaction such that its Pb–I–Pb bond angle in the inorganic layer is only slightly distorted (∼10°) away from the ideal 180° angle. As a result, the observed Pb–I–Pb bond angle of fluorinated (4FH-PPH)_2_PbI_4_ is ∼170°. To our knowledge, this is the smallest distortion observed in the fluorous short-chained (4FH-PPH)_2_PbI_4_ RP HP so far (see Table S4 in the Supporting Information).

Besides *E*_g_ determined by the Tauc plot, cyclic voltammetry (CV) measurement of the fluorous (4FH-PPH)_2_PbI_4_ RP HP has also been carried out. Its CV curve shows that this fluorinated 2D HP has the quite strong oxidation process. Thus, its ground-state oxidation potential, which corresponds to its HOMO level, can be calculated to be −5.40 eV by using eq S1 (in the Supporting Information). Thus, its energy bandgap diagram is also obtained (shown in Figure S12). Furthermore, the absorption properties of other (kinds of) fluorinated perovskites are usually shifted (from visible) to the UV region, making the capability to effectively detect visible light more challenging. Interestingly, the *E*_g_ (=2.22 eV) of this fluorinated short-chained (4FH-PPH)_2_PbI_4_ HP, which has the fluorous phase formation and the special L-type orientation, has not significantly increased like other kinds of fluorinated perovskites. Thus, it is believed that the reported fluorinated HP PD with capability to detect the wider range of light wavelengths (e.g., from some part of visible light to UV region) is desirable for the preparation of a potential broadband PD. In other words, all other fluorinated 2D HP photodetectors have their *E*_g_ values so high that they can be limited to use as the blue-light LED only. As shown in the inset of Figure 6a, the prepared fluorinated (4FH-PPH)_2_PbI_4_ HP film displays an orange color under visible light. When exposed to UV light, the color turned to greenish yellow. Furthermore, the film displays an emission peak near the absorption edge between 560 and 570 nm, as shown in Figure 6b. Its emission peak is consistent with the absorption spectrum and photo image of its film under UV illumination, as shown in the Figure 6a inset.

To investigate the fast charge transfer and charge carrier dynamics in a newly fabricated fluorinated short-chained (4FH-PPH)_2_PbI_4_ HP film, we have conducted TRPL under a 405 nm laser to study the carrier dynamic information shown in Figure 6c and Table S5. We fit the TRPL curves using a biexponential reconvolution model (see eqs S3 and S4 in the Supporting Information). As shown in Figure 6c, the average carrier lifetime (τ_ave_) of the fluorinated short-chained (4FH-PPH)_2_PbI_4_ HP film is calculated to be ∼0.323 ns (∼0.761 ns by using the triexponential reconvolution model), with a carrier lifetime of ∼15 ns. In contrast, the nonfluorinated HP film, (5H-PPH)_2_PbI_4_, which has almost no crystalline structure, does not show the PL and also leads to rapid decomposition in air (see Figures S6 and S11, respectively). The detailed fitting parameters are summarized in Table S5. Additionally, the average carrier lifetime (∼0.323 ns) of the fluorinated short-chained (4FH-PPH)_2_PbI_4_ HP film is notably faster than those of other reported halide perovskites, such as those using butylammonium (BA) and fluorinated butylammonium (FBA).^47^ The detailed comparisons of fluorous short-chained (4FH-PPH)_2_PbI_4_ HP with other structurally similar HPs (BA, FBA, PEA and FPEA) from the literature are listed in Table S5. As shown in items 1 and 2 (Table S5), the average carrier lifetime (τ_ave_) of BA is 236.9 ns, while the average carrier lifetime (τ_ave_) of a fluorinated BA (FBA) shows a faster lifetime of 137.3 ns. Similarly, FPEA shows a faster lifetime of 129.4 ns than that (=313.8 ns) of its nonfluorinated analogue, PEA. These results altogether affirm that incorporating the fluorine atom(s) into the organic spacer can significantly eliminate the nonradiative recombination ratio induced by the trap centers, resulting in a fast charge transfer.^47^ In fact, the reported fluorous short-chained (4FH-PPH)_2_PbI_4_ HP device shows about two orders faster in the carrier lifetime (of ∼0.323 ns) than those of FBA and FPEA-based HPs (see Table S5).

### 3.4 Photodetector Performance Analysis

**Figure 7:**
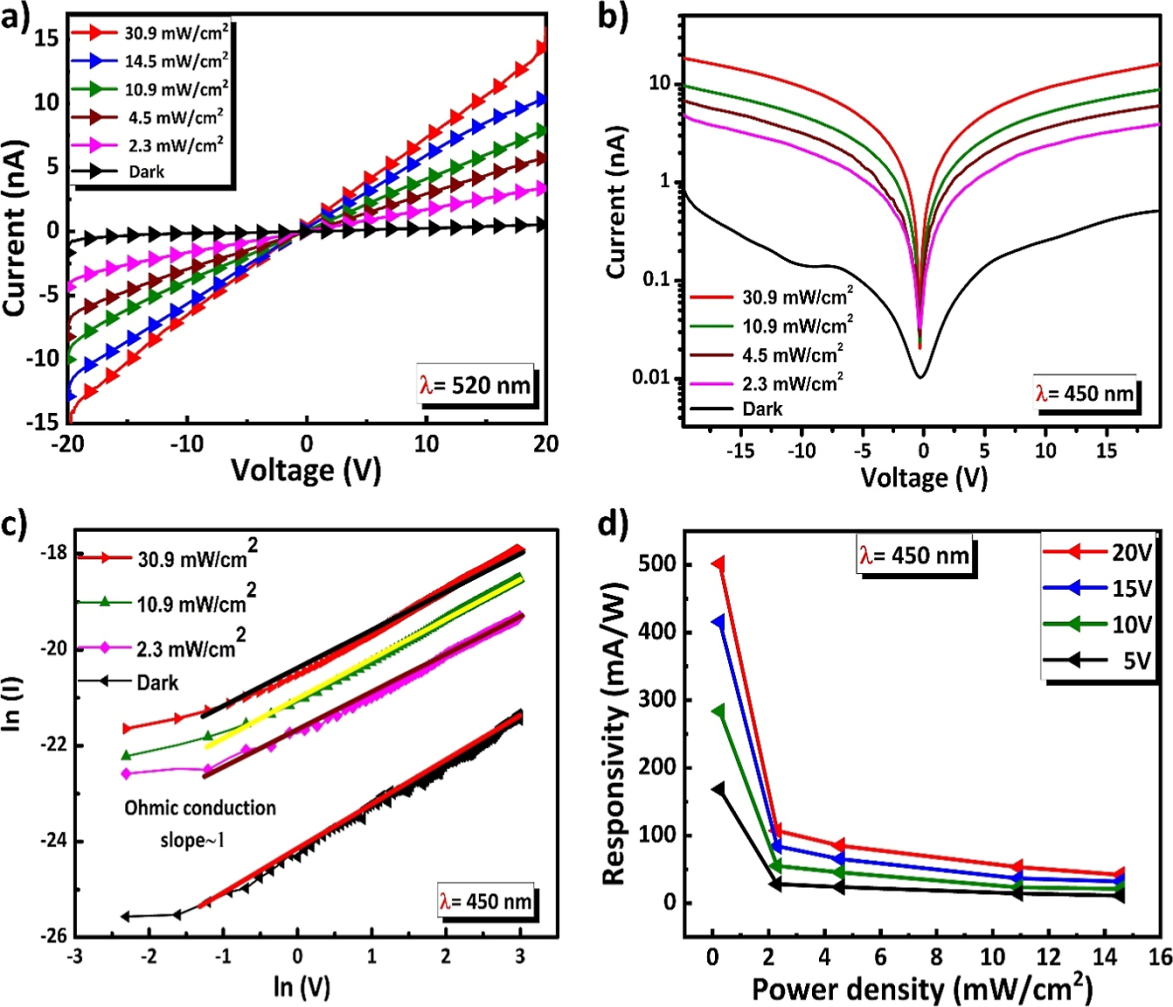
Photodetection performance of fluorinated (4FH-PPH)_2_PbI_4_ HP PD device under 450 nm showing (a) *I*–*V* characteristics under the dark state, (b) semilogarithmic *I*–*V* characteristics under the dark state, (c) double logarithmic *I*–*V* characteristics, and (d) illumination as a function of power density at an applied bias of 20 V.

To evaluate the optoelectronic properties of the fluorinated (4FH-PPH)_2_PbI_4_ 2D RP HP film, a lateral two-electrode structure photodetector (PD) has been fabricated by coating the film on the SiO_2_/Si substrate. The facile fabrication process can be used to construct a model PD device to further evaluate the photoelectric properties of the perovskite materials. The details of the fabrication processes are given in the device fabrication part. The schematic structure of the PD device is shown in Figure S13 (in the Supporting Information). The photoresponse have been then investigated by measuring the current–voltage (*I*–*V*) characteristics under two different laser sources (450 and 520 nm) with the tunable intensity. Figure 7a shows the *I*–*V* curves of the fluorinated (4FH-PPH)_2_PbI_4_ HP PD under dark and light irradiation with intensities ranging from 2.3 to 30.9 mW/cm^2^. All of the *I–V* curves from Figure 7a are able to exhibit excellent linearity, which confirm an Ohmic contact behavior between the 2D perovskite and electrodes.^48^ The increase in light current with increasing light intensity indicates the excellent photoresponse of the fluorinated 2D PD. The photocurrent of fluorinated (4FH-PPH)_2_PbI_4_ PD reached 15.1 nA under an illumination of 30.9 mW/cm^2^ at a bias of 20 V. The measured dark current (*I*_dark_) value of the PD device is ∼0.52 nA at the applied bias voltage of 20 V. The phototo-dark current ratio of fluorinated (4FH-PPH)_2_PbI_4_ reached 29 (=15.1/0.52) at the light illumination intensity of 30.9 mW/cm^2^, indicating the considerable signal-to-noise ratio behavior of this film-based PD device shown in Figure 6a (also see Figure 7a). The noise level of the signal-to-noise ratio has also been studied and is shown in Figure S14 (in the Supporting Information). Additionally, the semilog *I–V* characteristics plot of the fluorinated 2D PD device is also plotted and shown in Figure 7b. Besides 450 nm illumination, the results of the fluorinated 2D PD are also characterized by the 520 nm laser and are shown in Figures S15 and S16. Figure 7c is used to help in understanding the transport mechanism of the device.^49^ The typical double logarithmic plots of *I*–*V* characteristics are analyzed and plotted using power law, *I* α *V*^m^ (where m is slope of straight-line) under dark and upon 30.9, 10.9, and 2.3 mW/cm^2^ light illumination as shown in Figure 7c. These results show the straight-line characteristics in all the cases, and their corresponding slope values are found to be around 1.0. These results confirm that, the straight line *I*–*V* characteristics shown in Figure 6c follows the Ohmic conduction behavior.^49^Figure 7d shows responsivity (*R*) as a function of power density. To further characterize the performance of the fluorinated short-chained HP PD, the responsivity (*R*) under 450 nm laser light illumination has been plotted as a function of power density at different bias voltages shown in Figure 7d. The responsivity is calculated by eq 1(50)
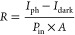
1where *I*_ph_ and *I*_dark_ are photocurrent and dark current, respectively. *P*_in_ is the incident laser power per unit area of the PD. A is the effective area of the device (=1.335 × 10^–5^ mm^2^). We have investigated the responsivity of the 2D HP PD at different bias voltages such as 5, 10, 15, and 20 V to assess its responses to the varying bias levels (Figure 7d). It can be noticed that this PD exhibits the highest responsivity of ∼502 mA/W under a 450 nm laser at an applied voltage of 20 V. These results demonstrate the robust performance of the fluorinated short-chain (4FH-PPH)_2_PbI_4_ HP PD under a 20 V bias. Therefore, our results have demonstrated a good correlation between the voltage change and the performance of the device, so the device performance is increased with increasing voltages. The responsivity values of fluorinated short-chained HP PD outperform those of the previously reported *n* = 1 hydrocarbon-based 2D perovskite PDs (see footnote 5 in Table 2). The detailed discussions of the comparisons are discussed below (also see Table 2). The improved responsivity of the fluorinated short-chained (4FH-PPH)_2_PbI_4_ HP device can be attributed to following reasons: first, the high dipole moment (μ = 17.74 D) of the fluorinated 4FH-PPH^+^ cation has the potential to enhance the separation of photogenerated carriers, promote the charge transport, and improve the overall performance of the fluorinated HP device.^31,41^ Second, the presence of fluorine atoms in the fluorinated organic moiety promotes the formation of the fluorous phase and rare hydrophobic noncovalent interactions, such as C–H···F, C–H···O and C–H···H–C improper HBs, and C–F···F–C halogen bond interactions.^37,39^ Third, the homogeneous and pinhole-free nature of this prepared fluorinated RP-type perovskite film allowed for the good contacts between the electrodes and the perovskite film, contributing to the enhanced air stability at ambient temperature and to the improved performance of the PD device.^51^ Thus, with these overall features, the reported fluorinated short-chained (4FH-PPH)_2_PbI_4_ HP PD shows much higher responsivity than those of other 2D HP PD (also see Table 2).

**Table 2:** Comparison of the Present *n* = 1 Film-Based Fluorinated Short-Chained (4FH-PPH)_2_PbI_4_ PD Performances with Other 2D HP Photodetectors (PDs) Reported Previously^a^

item	material	*E*_g_ (eV)	responsivity (mA/W)	detectivity (cm Hz^1/2^ W^–1^)	response/recovery time	wavelength (nm)	bias voltage (V)	stability (in air)	ref.
1	(BA)_2_PbI_4_	2.35	NA	NA	0.5/0.2 s	Xe lamp	5	7 days	(53)
2a	(BA)_2_PbI_4_	2.33	3.00	NA	28.4/27 ms	white light	30	NA	(54)
2b	(BA)_2_(MA)Pb_2_I_7_	2.11	7.31	NA	8.4/7.5 ms	white light	30	NA	
2c	(BA)_2_(MA)_2_Pb_3_I_10_	2.00	12.78	NA	10/7.5 ms	white light	30	NA	
3a	(iBA)_2_PbI_4_	2.33	4.78	NA	49/61 ms	532	1.5	NA	(55)
3b	(iBA)_2_(MA)_3_Pb_4_I_13_	1.71	117.09	NA	16/15 ms				
3c	(iBA)_2_(MA)_3_Pb_4_I_13_	NA	72	3.61 × 10^10^	72/31 ms	532	1.5	NA	(56)
	(iBA)_2_(MA_0.4_FA_0.6_)_0.9_(Cs_0.1_)_3_Pb_4_I_13_	NA	400	1.68 × 10^12^	43/22 ms	532	1.5	9 days	
4	(HA)_2_PbI_4_	2.36	NA	NA	0.2/0.1 s	Xe lamp	5	7 days	(53)
5a	(OA)_2_PbI_4_	2.38	NA	NA	0.3/0.1 s	Xe lamp	5	7 days	(53)
		2.42	2.89		0.2/0.5 s	470		NA	(57)
			7.44		0.4/0.2 s	530			
5b	(OA)_2_(MA)Pb_2_I_7_	2.19	0.12	NA	0.17/0.47 s	470	5	NA	(57)
			0.24		0.38/0.35 s	530			
6	(3AMPY)EAPb_2_Br_7_*	2.64	0.01	4.6 × 10^8^	NA	800	10	NA	(58)
7	(4AMP)Cs_2_Pb_3_Br_10_*	2.36	12	6.5 × 10^10^	193/436 μs	405	10	NA	(59)
8	(*R*-BPEA)_2_PbI_4_*	2.34	2.1	3 × 10^11^	242/460 μs	520	10	NA	(60)
9a	(PEA)_2_PbI_4_	2.36	5.4	1.07 × 10^13^	NA	solar light	5	NA	(61)
		2.33	1070	2.96 × 10^11^	43/45 ms	460	3	NA	(62)
9b	(PEA)_2_MA_3_Pb_4_I_13_	NA	440	30.38× 10^12^	20.8/20.6 μs	570	0	8 days	(63)
10	(*o*F-PEA)_2_PbI_4_*	2.36	used for structure and bandgap study			NA	NA	NA	(64,65)
11	(*m*F-PEA)_2_PbI_4_*	2.35	used for solar cell study			AM 1.5 G		30 days	
12	(*p*F-PEA)_2_PbI_4_*	2.37	used for solar cell study			AM 1.5 G	NA	30 days	(65,66)
13	(*p*F-PMA)_2_PbI_4_*	2.36	used for structure and bandgap study			NA		NA	(67)
14	(CF_3_-PMA)_2_PbI_4_*	2.52	used for structure and bandgap study			NA	NA	NA	(68)
15	(5FBzA)_2_PbI_4_*	2.52	used for solar cell study			AM 1.5 G	NA	45 days	(69)
16	(L_f_)_2_PbI_4_*	2.60	used for structure and bandgap study			NA	NA	NA	(70)
**17**	**(4FH-PPH)**_**2**_**PbI**_**4**_	**2.22**	168.2	1.92 × 10^10^	43/46 ms	450	5	49 days	**this work** (also see footnote note 3)
			142.5	1.63 × 10^10^	45/48 ms	520			
			501.9	5.73 × 10^10^	42/46 ms	450	20		
			463.1	5.29 × 10^10^	43/49 ms	520			

a Note 1. MA—methylammonium, FA—formamidinium, EA—ethylammonium, BA—butylammonium, iBA—iso-butylammonium, HA—hexylammonium, OA—octylammonium, 3AMPY—3-(aminomethyl)pyridinium, 4-AMP—4-(aminomethyl)piperidinium, BPEA—bromo phenylethylammonium and PEA—phenylethylammonium, *x*PEA—phenylethylammonium [*x* = *o* (ortho), *m* (meta) and *p* (para)], PMA—phenylmethylammonium, 5FBzA—pentafluorobenzyl ammonium and L_f_—C_9_H_6_F_13_NH_3_^+^. Note 2. (*) = single crystal-based. Note 3. The 2D perovskites corresponding to items of 1, 2a, 3a, 4, 5a, and 8–17 share the common chemical formula of L_2_A_*n*–1_*M*_*n*_X_3*n*+1_ where *n* = 1 shown in page 3.

**Figure 8:**
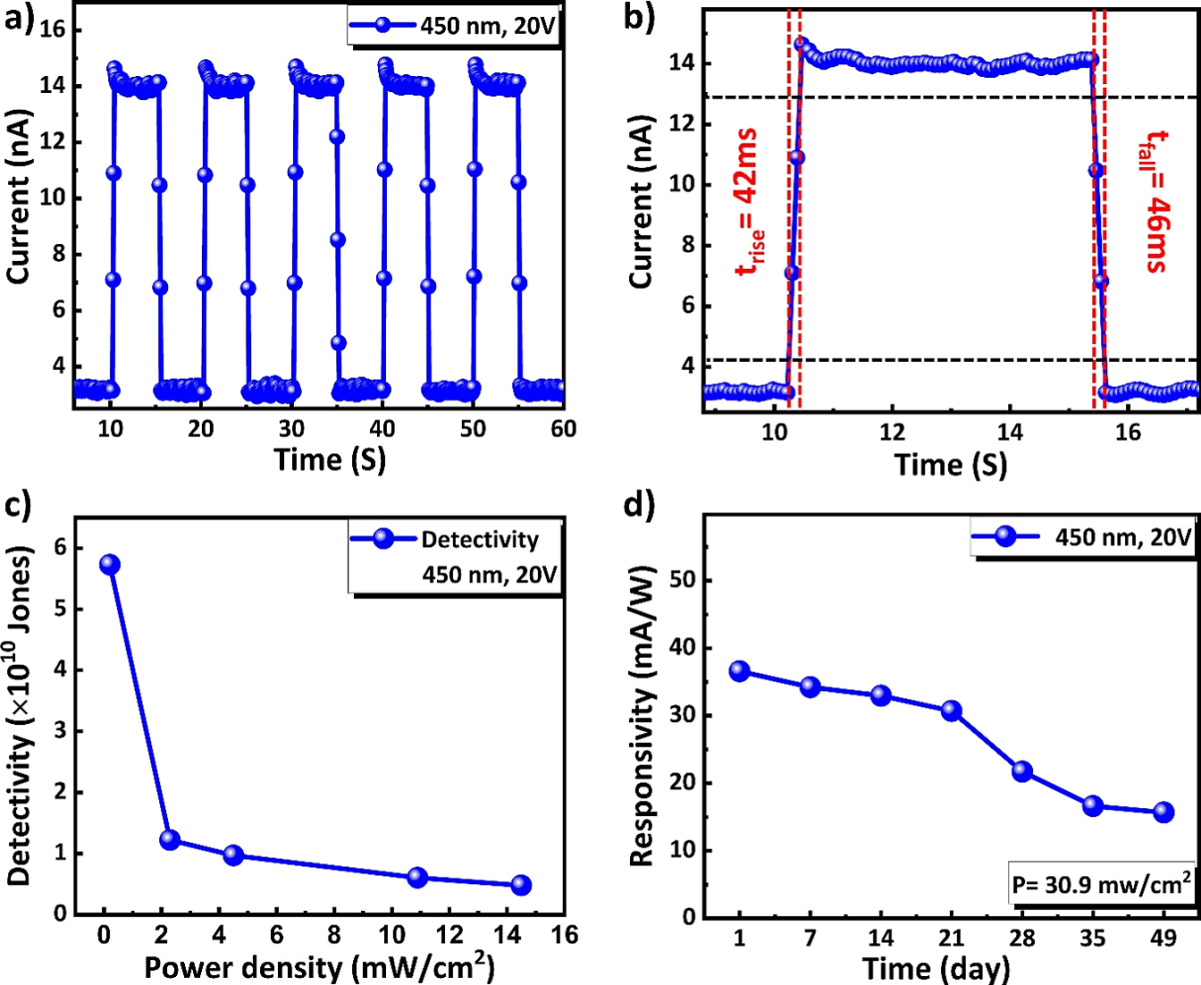
Photodetection performance of a fluorinated short-chained (4FH-PPH)_2_PbI_4_ HP PD device under 450 nm showing (a) time-dependent photocurrent response under illumination of 30.9 mW/cm^2^, (b) *I*–*t* curve in a cycle at 20 V, (c) detectivity (D*) as a function of the power density and, (d) stability of the PD device stored in air without any encapsulation for up to 49 days.

Furthermore, Figure 8a shows the time response of the fluorinated 2D HP PD. The fast responses to light illumination are known to be crucial for high-performance PD for commercial applications. The time response of the fluorinated 2D HP PD has been measured (under 450 nm at the power density of 30.9 mW/cm^2^) by performing the current–time (*I*–*t*) measurements at an applied bias voltage of 20 V in ambient condition and shown in Figure 8a. As shown in Figure 8b, the fluorinated 2D device exhibits high repeatability over many ON/OFF switchings of light illumination at a 30.9 mW/cm^2^ intensity. The ON/OFF ratio is found to be >15. Additionally, we have analyzed the response and recovery by taking one ON and one OFF cycle of the *I*–*t* curve. The response/recovery times have been found to be 42/46 ms, respectively. The corresponding time of the device is defined as the rise time of 10% to 90% of the device and the fall time of 90% to 10%. Figure 8c shows the detectivity (D*) as a function of power density. In addition, based on eq 2, we calculated the detectivity (D*) of fluorinated 2D HP PD to evaluate the photodetection properties.^52^
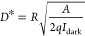
2where *q* is the unit electron charge, *A* is the effective area (see above) of the device, and *I*_dark_ is the dark current. The D* of the PD is also determined under 450 nm at the power density of 30.9 mW/cm^2^ and the bias voltage of 20 V. As shown in Figure 8c, D* of fluorinated short-chained (4FH-PPH)_2_PbI_4_ HP PD increases with decreasing power density, and the maximum value of D* is found to be 5.73 × 10^10^ Jones (cm Hz^1/2^ W^–1^). Both parameters *R* and D* of fluorinated 2D HP PD show good photodetection performances. Moreover, the stability of fluorinated 2D HP PD has also been studied (under 450 nm at the power density of 30.9 mW/cm^2^ and the high bias voltage of 20 V) in air for 49 days without encapsulation by measuring the variation of responsivity shown in Figure 8d. This stability test is also affirmed by water contact angle studies shown in Figure S7. Is believed that the repeated uses of this high voltage of 20 V during the monitoring process might likely considerably affect the device performance and decrease in responsivity over the days. Nevertheless, this is the first time that a 2D HP with the fluorinated short-chained segments in the organic spacer has shown this level of stability (see Table 2). This stability is higher than previously reported *n* = 1 hydrocarbon, aromatic, and cyclic hydrocarbon-based PDs reported by other groups. The detailed comparisons of the performance of this fluorous short-chained (4FH-PPH)_2_PbI_4_ HP PD with other *n* = 1 perovskite PDs are provided in Table 2.

Although it is well-known that 3D HP-based PDs offer high responsivity, their stability issues prevent them from being commercialized. To address this, researchers have developed short-chained 2D HP-based PDs, which trade off light absorption and responsivity.^53−56^

A similar strategy has improved the stability of 3D perovskite-based solar cells since 2014. In addition, the 2D photoconductive responses have been investigated in perovskite-based PDs recently in this journal.^53−56^ For example, as shown in items 2a–c (in Table 2), which are also been reported in ref (62), the responsivity values of one-layered perovskite, two-layered perovskite, and three-layered perovskite whose *n* = 1, 2, 3, respectively, from RP-type 2D perovskite-based PDs with formula of L_2_A_*n*–1_*M*_*n*_X_3*n*+1_ are found to be 3.00, 7.31, and 12.78 mA/W, respectively^54^ (which is only about the ∼0.59, 1.46 and 2.55% of the reported value in this work, respectively). Thus, so far, almost all the reported photoresponsivity values,^53−70^ from the normal short-chained hydrocarbon-based 2D PD devices, are much smaller than the 502 mA/W value of this fluorinated 2D HP PD reported here.

Additionally, the reported fluorinated (4FH-PPH)_2_PbI_4_ HP PD device reveals good stability over a 49 day period in ambient temperature, which has been described and mentioned above and can be summarized as below. (1) Fluorous phase formation: the incorporation of fluorine atoms creates a unique fluorous phase within the perovskite structure, enhancing its fluorophobicity and moisture tolerance. (2) Compact crystal packing: the edge-to-face arrangement of 4FH-PPH^+^ spacers, influenced by hydrogen bond interactions, leads to dense crystal packing. This arrangement fosters rare hydrophobic interactions (like C–H···F) and contributes to a strong water-repellent layer, preventing moisture infiltration. (3) Film morphology: the use of fluorinated short-chained cationic spacers promotes a uniform, pinhole-free film, which enhances device performance and stability at ambient temperatures. And (4) hydrophobic surface: the water contact angle of 86.5° indicates a hydrophobic nature, further aiding in moisture prevention. Therefore, we believe that the combination of the unique structural orientation, hydrophobicity due to blue-shifting HBs, fluorophobicity from the fluorous phase, and a uniform film morphology all contribute to the enhanced stability of the fluorinated short-chained (4FH-PPH)_2_PbI_4_ HP photodetector.

Furthermore, the performance of the fluorinated 2D HP-based PD has also been evaluated under 520 nm laser light illumination, as shown in Figure S15. Under 520 nm light illumination, the fluorinated short-chained (4FH-PPH)_2_PbI_4_ PD shows a response similar to that of 450 nm laser light illumination. However, the responsivity and response/recovery times are found to be slightly less comparable to those under the 450 nm light illumination, which are attributed to the less optical absorption ability upon the 520 nm laser light illumination. Again, the overall photodetection performance of the reported devices is much better when compared with those of the reported works from other hydrocarbon-based 2D HP PDs. In summary, in the case of the fluorinated short-chained 2D film-based PD device, the introduction of the fluorinated short-chained containing organic spacer cation (4FH-PPH^+^) into the 2D perovskite-based PD can not only widen the optical absorption range but also enhance the device stability and performance. These results show that photoelectric devices with excellent performance can be constructed by engineering the interlayered fluorinated cation. These findings demonstrate that 2D interlayered engineering can be used to fabricate the good stability and excellent performance of the fluorinated short-chained 2D HP PDs that can well operate while its energy bandgap is also tunable.

In addition, as shown in item 3c (in Table 2), Dong et al. have successfully incorporates different concentrations of FA and Cs cations (i.e., mixed cations) into the quasi-2D perovskites PD of (iBA)_2_(MA)_3_Pb_4_I_13_ to modulate their thin-film qualities and the device performances.^56^ The responsivity of (iBA)_2_(MA)_3_Pb_4_I_13_ and mixed cations (iBA)_2_(MA_0.4_FA_0.6_)_0.9_(Cs_0.1_)_3_Pb_4_I_13_ have been found to be 72 and 400 mA/W, respectively, under a bias voltage of 1.5 V at 532 nm illumination (which is about ∼14.3 and ∼79.7% of the reported value in this work, respectively). However, these types of PD devices have only showed stability for a few days (i.e., up to 8 days), starting to degrade by the ninth day of exposure. Therefore, the normal short-chained hydrocarbon-based 2D HPs PD devices are lower in the responsivity values than that (=502 mA/W) of this fluorous short-chained (4FH-PPH)_2_PbI_4_ HP PD. Moreover, this fluorinated PD device has shown good stability for 49 days in air at room temperature, which is significantly better than that of the previously reported normal short-chained hydrocarbon-based PDs.

Currently, Lu’s lab also wants to investigate the impact that incorporating a fluorinated chain into the PEA and PMA spacers can have on the PD device performance. Our past experience has shown that this fluorinated short-chain incorporation can likely result in the significantly improved performance of the device, which has actually been demonstrated as an outstanding PD in the previous study reported in the top materials journal.^30^ In this study, it has been also found that the device based on the fluorinated (4FH-PPH)_2_PbI_4_ HP PD has displayed good PD performances in photoresponsivity, detectivity, time response, and stability when compared with other normal hydrocarbon-based short-chained 2D RP HP PDs depicted in Table 2. To our knowledge, the data reported herein represent the first instance of a fluorinated 2D HPs based PD exhibiting good stability for over 7 weeks. All these results suggest that the formation of the fluorous phase in the (4FH-PPH)_2_PbI_4_ HP can give rise to the good performances and moisture-tolerant stability.

## 4 Conclusions

It has been found for the first time that a new type of fluorinated piperidinium film-based 2D RP HP, abbreviated as (4FH-PPH)_2_PbI_4_, which exhibits the relatively small energy bandgap (*E*_g_) due to the incorporation of the fluorinated short-chained segment of −CF_2_CF_2_H moiety into its organic spacer, has been successfully prepared. The observed energy bandgap of this fluorinated short-chained (4FH-PPH)_2_PbI_4_ HPs is found to be 2.22 eV, which is the lowest value among all the fluorinated perovskites reported in Table 2. To name a few, (F-PEA)_2_PbI_4_, (F-PMA)_2_PbI_4,_ (CF_3_-PMA)_2_PbI_4_ and (5FBzA)_2_PbI_4_ are some of their examples. After analyzing the different structural properties such as bond angle, bond length, and other rare noncovalent interactions (NCIs), we have confirmed that this decrease in *E*_g_ is a result of a slight distortion of the Pb–I–Pb bond angle (169.49°) of the fluorinated (4FH-PPH)_2_PbI_4_ HP from the ideal of a 180° angle. This slight angle distortion is believed to be eased by both the fluorophobic property of the fluorous side-chain present in the crystal structure and the help of a hook-type (with a L-shape) orientation from the unusual blue-shifting HB interaction. To our knowledge, this is the smallest distortion angle (10.51°) observed among all the fluorinated 2D RP lead iodide-based perovskites reported so far (see Table S4). In addition, the presence of the rare hydrophobic NCIs such as C–H···Y (where Y = I, O, F) improper HBs and C–F···F–C halogen bond interactions in the fluorinated perovskite structure effectively covers the voids present in the crystal packing. As a result, a good water-tolerant fluorous layer with a water contact angle of 86.5° has been formed to block the infiltration of water molecules into the perovskite system. Thus, the demonstrated 2D fluorinated perovskite photodetector (PD) exhibits a good photoresponsivity of 502 mA/W under 450 nm light illumination at a 20 voltage (V) where its photoresponsivity is largest among all the presented HP materials in Table 2 and at least 4 times higher than those other short alkyl-chained 2D HP PDs—e.g., items 2 and 3 in Table 2. Furthermore, this fluorinated 2D PD shows the impressive response/recovery times of 42/43 ms and a remarkably high photodetectivity of 5.73 × 10^10^ Jones under 450 nm. Additionally, the responsivity of the poly-fluorinated perovskite-based 2D PD device still retains 43% (without encapsulation) of its initial value after 49 days under ambient conditions.

The combined experimental studies of the fluorinated short-chained (4FH-PPH)_2_PbI_4_ perovskite films and devices confirmed that the presence of both fluorophobicity and other rare NCIs can not only show the good stability and excellent performance but also lower the *E*_g_ of the fluorinated perovskite material. In addition, the fluorinated (4FH-PPH)_2_PbI_4_ HP material can serve as a valuable reference for the development of a highly detective, long-term stable, and fast photoresponse PD as well as for the advancement of future integrated electronic and optoelectronic devices beyond conventional materials and techniques. Further research to explore the use of a suitable poly fluorinated organic spacer in the 2D perovskites, with the aim of protecting the fluorinated Sn-based perovskites from the moisture and air, is underway in Lu’s lab.

## Supporting Information Available

Detailed synthesis of materials, computational methods, crystallization method, device scheme, other structural tables, HR-TEM data, TRPL data, and NMR characterization spectra (PDF)
(CIF)

## Supplementary Material

am4c18202_si_001.pdf

am4c18202_si_002.cif
