# Supplementary material for: Fast, Highly Stable, and Low-Bandgap 2D Halide Perovskite Photodetectors Based on Short-Chained Fluorinated Piperidinium as a Spacer
Source: ACS Appl Mater Interfaces. 2024 Dec 16;17(1):1743–59. doi: 10.1021/acsami.4c18202 (PMC11783540; doi:10.1021/acsami.4c18202)
Supplement: Supplementary file 1 — am4c18202_si_001.pdf [file am4c18202_si_001.pdf]

Supporting Information

Fast, highly stable and low-bandgap 2D halide perovskite photodetectors based on a short-chained fluorinated piperidinium as a spacer

Norman Lu,[1,2] Gurumallappa Gurumallappa,[1,2] Jitendra Singh,[3] Ka Long Chan,[1] Eskedar Tessema,[1] Pin-Yu Liu,[1] Hema Mylnahalli Krishnegowda,[1] Karthik Chimatahalli Shanthakumar,[4] Jinn-Hsuan Ho,[5] Yu-Chiang Chao,[6] Lam-Gia-Hao Dao,[7] Sumedh Shirsat,[7] Meng-Lin Tsai[7]*

1. *Institute of Organic and Polymeric Materials, National Taipei University of Technology, Taipei 106, Taiwan (ROC). E-mail: normanlu@mail.ntut.edu.tw*
2. *Graduate Institute of Energy and Optoelectronic Materials National Taipei University of Technology, Taipei 106, Taiwan (ROC).*
3. *Department of Physics, Udit Narayan Post Graduate College Padrauna Kushinagar, affiliated by Deen Dayal Upadhyaya Gorakhpur University, Uttar Pradesh-274304-India.*
4. *Department of Chemistry, SJCE, JSS Science and Technology University, Mysuru-570 006, Karnataka, India.*
5. *Department of Chemical Engineering, National Taiwan University of Science and Technology, Taipei 106335, Taiwan.*
6. *Department of Physics, National Taiwan Normal University, Taipei 106, Taiwan.*
7. *Department of Materials Science and Engineering, National Taiwan University of Science and Technology, Taipei 106335, Taiwan.*

TABLE OF CONTENTS

1. Synthesis of materials

1.1. Synthesis of tert-butyl 3-hydroxymethylpiperidine-1-carboxylate (**1**) SI-4
1.2. Synthesis of tert-butyl 3-((tosyloxy)methyl)piperidine-1-carboxylate (**2**) SI-5
1.3. Synthesis of tert-butyl 3-((2,2,3,3-tetrafluoropropoxy)methyl)piperidine-1-carboxylate (**3**) SI-6
1.4. Synthesis of 3-((2,2,3,3-tetrafluoropropoxy)methyl)piperidinium iodide (4FH-PPHI) (**4**) SI-7
1.5. Synthesis of tert-butyl 3-(propoxymethyl)piperidine-1-carboxylate (**5**) SI-8
1.6. Synthesis of 3-(propoxymethyl)piperidinium iodide (5H-PPHI) (**6**) SI-9

2. Experimental and computational study

Electrochemistry method SI-10
Computational method SI-10
Figure S1. Calculated molecular dipole moments of the 5H-PPH$^+$ and 4FH-PPH$^+$ cations SI-11

3. Crystallization, structural and morphology data

Figure S2. XPS spectrum of $(4FH\text{-}PPH)_2PbI_4$ HP SI-12
Figure S3. Crystallization method and microscope image of $(4FH\text{-}PPH)_2PbI_4$ HP crystal SI-13
Figure S4. The C−H···Y improper HB and C−F···F halogen bonding interactions SI-14
Figure S5. PXRD and FT-IR spectra of $(4FH\text{-}PPH)_2PbI_4$ HP film SI-15
Figure S6. Comparison PXRD spectra of $(4FH\text{-}PPH)_2PbI_4$ and $(5H\text{-}PPH)_2PbI_4$ HPs films SI-16
Figure S7. Water contact angle measurement of $4FH\text{-}PPH)_2PbI_4$ HP film SI-17
Figure S8. Grain size distribution analyses of $(4FH\text{-}PPH)_2PbI_4$ HP powder SI-18
Figure S9. HR-TEM image for the EDS mapping of $(4FH\text{-}PPH)_2PbI_4$ HP SI-18
Figure S10. EDS spectrum of $(4FH\text{-}PPH)_2PbI_4$ HP SI-19
Figure S11. HR-TEM and EDS-mapping of non-fluorinated $(5H\text{-}PPH)_2PbI_4$ HP SI-20

4. Device scheme and photodetector performance

Figure S12. Schematic energy band diagram of $(4FH\text{-}PPH)_2PbI_4$ HP SI-21
Figure S13. Schematic illustration of the fabricated $(4FH\text{-}PPH)_2PbI_4$ HP PD SI-21
Figure S14. Signal-to-noise measurements of the $(4FH\text{-}PPH)_2PbI_4$ HP PD SI-22
Figure S15. The photodetection performance of $(4FH\text{-}PPH)_2PbI_4$ HP PD under 520 nm SI-23
Figure S16. Semi-log *I-V* characteristics of $(4FH\text{-}PPH)_2PbI_4$ HP PD SI-24

5. Structural table section

Table S1. Selected bond lengths in $(4FH\text{-}PPH)_2PbI_4$ HP SI-25
Table S2. Selected bond angles in $(4FH\text{-}PPH)_2PbI_4$ HP SI-26
Table S3. N−H···I and C−H···Y type of hydrogen bonds in $(4FH\text{-}PPH)_2PbI_4$ HP SI-27
Table S4. Comparison of structural and optical properties of $(4FH\text{-}PPH)_2PbI_4$ HP SI-28
Table S5. Comparison of TRPL parameters of $(4FH\text{-}PPH)_2PbI_4$ HP SI-29

6. The NMR Spectra of prepared compounds

Figure S17. ^{1}H NMR spectrum of compound (**1**) SI-31
Figure S18. ^{1}H NMR spectrum of compound (**2**) SI-31

Figure S19. ^{1}H NMR spectrum of compound (**3**) SI-32
Figure S20. ^{13}C NMR spectrum of compound (**3**) SI-32
Figure S21. ^{19}F NMR spectrum of compound (**3**) SI-33
Figure S22. ^{1}H NMR spectrum of compound (**4**) SI-33
Figure S23. ^{13}C NMR spectrum of compound (**4**) SI-34
Figure S24. ^{19}F NMR spectrum of compound (**4**) SI-34

7. References SI-35

1. Synthesis of materials

1.1. Synthesis of tert-butyl 3-hydroxymethylpiperidine-1-carboxylate (1)

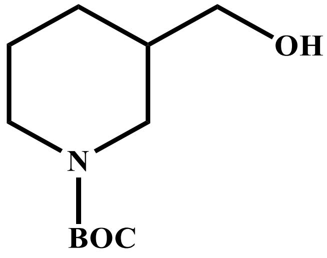

(1)

In 1L single-necked round-bottomed flask, 3-hydroxymethylpiperidine (5g, 75 mmol) was dissolved in a mixture of CH_3CN (64 mL) and saturated aqueous $NAHCO_3$ solution (21 mL). To this was added di-tert-butyl dicarbonate (21g, 94 mmol) under ice cooling.[1] The flask was then loosely capped with a rubber septum that contained two needles to release CO_2 produced in the reaction. The reaction was allowed to stir at room temperature overnight under ambient atmosphere for 16 hours. The resulting solution was extracted three times with ethyl acetate and washed one time with 150 mL of saturated brine solution. The organic phase was then dried over Na_2SO_4 and all the solvent is removed under a rotavapor to give a white solid of **1**.

Analytical data of (**1**): Yield: 95%; ^{1}H NMR (300 MHz, $CDCl_3$, room temperature): δ(ppm)= 4.0-3.55 (2H, br, H2, H6), 3.6-3.4 (2H, d, $^3J_{HH}$ = 6.0 Hz, PP-C$\underline{H}_2$OH), 3.3-2.6 (2H, br, H2, H6), 2.4-2.1 (1H, br, OH), 1.8-1.5 (3H, m, H3, H4, H5), 1.5-1.3 (10H, s, *t*-butyl, H4), 1.3-1.1(2H, m, H5); FT-IR υ(cm^{-1}): 3483 ($\upsilon_{CH2\underline{OH}}$), 3004, 2972, 2936, 2890, 2863, 2817 ($\upsilon_{pp\text{-}CH2}$), 1668 ($\upsilon_{C=O}$); GC/MS (m/z): 16.0 min, 215 (M^+), 159 (M^+ - *t*-butyl), 142 (M^+ - *t*-butoxide), 129 (M^+ - *t* -butyl – CH_2OH), 114 (M^+ - BOC), 84 (PP^+), 57 (*t* -butyl$^+$).

1.2. Synthesis of tert-butyl 3-((tosyloxy)methyl)piperidine-1-carboxylate (2)

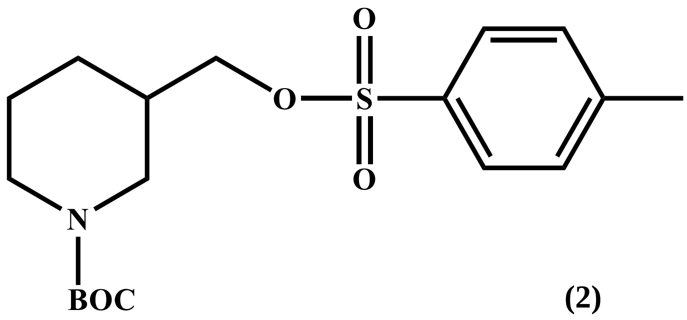

(2)

In 250 mL single-necked round-bottomed flask, *tert*-butyl 4-hydroxypiperidine-1-carboxylate (1.0g, 4.65 mmol) was dissolved in anhydrous CH_2Cl_2 (50 mL) and then the mixture was cooled to 0 °C. To this were added triethylamine (4.7 mL, 33.5 mmol), 4-dimethylaminopyridine (0.127g, 1.10 mmol) and Tosyl chloride (5.10g, 26.8 mmol). Under an atmosphere of N_2, the resulting solution was stirred for 16 hours as it warmed up to room temperature.[2] The resultant mixture was extracted three times with ethyl acetate after being diluted with saturated aqueous NaOH. The combined organic layer was further purified with brine and deionized H_2O, dried over Na_2SO_4, filtered, and concentrated under reduced pressure to give **2** as a colorless liquid.

Analytical data of (**2**): Yield: 90%; ^{1}H NMR (300 MHz, $CDCl_3$, room temperature δ(ppm)= 7.77 (2H, d, $^3J_{HH}$ = 8.4Hz, Ph-H2, H6), 7.33 (2H, d, $^3J_{HH}$ = 8.0Hz, Ph-H3, H5), 3.86 (2H, d, $^3J_{HH}$ = 6.0Hz, PP-C$\underline{H}_2$O), 3.55-3.80 (2H, m, H2, H6), 2.55-2.90 (2H, m, H2, H6), 2.43 (3H, s, Ph-C$\underline{H}_3$), 1.46-1.78 (3H, m, H3, H4, H5), 1.41 (9H, s, *t*-butyl), 1.1-1.3 (2H, m, H4, H5); FT-IR υ(cm^{-1}): 2974, 2934, 2859 ($\upsilon_{pp\text{-}CH2}$), 1692 ($\upsilon_{BOC\text{-}C=O}$), 1599 ($\upsilon_{py\text{-}C=C}$), 1355, 1173 ($\upsilon_{Ts\text{-}S=O}$, stretch); GC/MS (m/z): 26.1min, 369 (M^+), 310 (M^+ - *t*-butyl), 268 (M^+ - BOC), 114 (M^+ - BOC - tosylmethylsulfonyl), 98 (methylpiperidine$^+$), 57 (*t*-butyl$^+$).

1.3. Synthesis of tert-butyl 3-((2,2,3,3-tetrafluoropropoxy)methyl)piperidine-1-carboxylate (3)

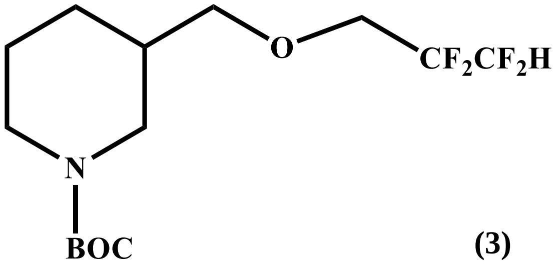

(3)

A magnetic stirrer and vacuum system were equipped in a 250 mL double-necked round-bottom flask. The air and moisture were pumped out of the flask using the vacuum system. To confirm the reaction system was in a nitrogen environment, this process was performed multiple times. $HCF_2CF_2CH_2OH$ (0.57g, 4.33 mmol) and CH_3ONa (0.73g, 4.06 mmol) were added into the two necked round bottom flask.[3-9] Under the N_2 atmosphere, this mixture was then stirred for overnight. The following day, the methanol was removed under vacuum, and the reaction was driven to the product side leaving a white powder. Then 50 mL of freshly distilled anhydrous THF was added into the reaction flask under ice cooling in an external ice bath. Then, **2 (**1.0g, 2.71 mmol) was added into the reaction mixture and the mixture was stirred for overnight while gradually warming to room temperature. After confirming that the ligand was formed by using GC/MS analysis, the reaction was quenched by adding water. The entire content of the flask was then transferred to a 1 L separatory funnel and extracted three times with CH_2Cl_2 and deionized H_2O. The organic phase was then dried over Na_2SO_4 and concentrated under reduced pressure to give a colorless liquid of **3**.

Analytical data of (**3**): Yield: 70%; ^{1}H NMR (600 MHz, DMSO-d_6, room temperature δ(ppm)= 6.48 (1H, tt, $^2J_{HF}$ = 51.6Hz, $^3J_{HF}$ = 6.0Hz, $CF_2\underline{H}$), 3.91 (2H, t, $^3J_{HF}$ = 16.2Hz, $OC\underline{H}_2CF_2$), 3.87-3.69 (2H, m, H2, H6), 3.46-3.37 (2H, m, PP-$C\underline{H}_2$), 2.87-2.54 (2H, m, H2, H6), 1.75-1.63 (2H, m, H3, H4), 1.62-1.22 (1H, m, H5), 1.39 (9H, s, *t*-butyl), 1.36-1.12 (2H, m, H4, H5); ^{13}C NMR (150 MHz, DMSO-d_6, room temperature): δ(ppm)= 154.47 (1C, s, N-$\underline{C}OOC(CH_3)_3$),118.01 – 114.10 (1C, $CH_2\underline{C}F_2CF_2H$), 111.81 – 107.90 (1C, $CH_2\underline{C}F_2CF_2H$),78.87 (1C, s, N-$COO\underline{C}(CH_3)_3$), 74.42 (1C, s, PP-$\underline{C}H_2O$), 67.56 (1C, t, $^2J_{CF}$ = 26.3Hz, $O\underline{C}H_2CF_2CF_2H$), 46.85 (1C, s, C6), 44.76 (1C, s, C2), 36.00 (1C, s, C3), 28.51(3C, s, N-$COOC(\underline{C}H_3)_3$), 26.73 (1C, s, C4), 24.22 (1C, s, C5); ^{19}F NMR (564 MHz, DMSO-d_6, room temperature): δ (ppm)= -124.84 (2F, $CH_2C\underline{F}_2CF_2H$), -139.56 (2F, $CH_2CF_2C\underline{F}_2H$); FTIR υ(cm^{-1}): 2977, 2933, 2862 ($\upsilon_{pp\text{-}CH2}$), 1687 ($\upsilon_{BOC\text{-}C=O}$), 1149, 1102 ($\upsilon_{CF2}$); GC/MS (m/z): 16.2min, 329 (M^+), 270 (M^+ - *t*-butyl), 256 (M^+ - *t*-butoxide), 228 (M^+ - BOC), 158 (M^+ - *t*-butyl - $CH_2CF_2CF_2H$), 114 (M^+ - BOC - $CH_2CF_2CF_2H$), 57 (*t*-butyl$^+$).

1.4. Synthesis of 3-((2,2,3,3-tetrafluoropropoxy)methyl)piperidinium iodide (4FH-PPHI) (4)

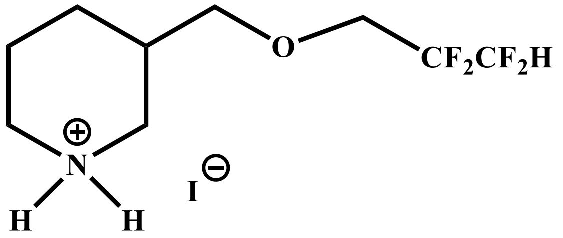

(4)

tert-butyl 3-((2,2,3,3-tetrafluoropropoxy)methyl)piperidine-1-carboxylate (Compound ***3***) (0.5g, 1.52 mmol) was added to a 250 mL round-bottomed flask and 10 mL of chloroform was added to dissolve **3**. To this was added 57 wt% hydroiodic acid (0.38 mL, 2.88 mmol) containing no stabilizer in an ice bath. [7 10] The reaction mixture was stirred under N_2 (g) at 60 °C for 2 hr. Then the solvent was removed under vacuum, and the product was washed with a copious amount of diethyl ether and dried under vacuum for several days to give the white powder of **4** (also abbreviated as 4FH-PPHI). Furthermore, theoretical calculations are performed for the 4FH-PPH^+ cation.

Analytical data of (**4**): Yield: 70%; m.p. 119-121 °C; ^{1}H NMR (600 MHz, DMSO-d_6, room temperature): δ(ppm)= 8.70-8.00 (2H, PP-N$\underline{H}_2^+$), 6.53 (1H, tt, $^2J_{HF}$ = 51.6Hz, $^3J_{HF}$ = 5.4Hz, $CF_2\underline{H}$), 3.93 (2H, t, $^3J_{HF}$ = 12.6Hz, $O C\underline{H}_2 CF_2$), 3.54-3.41 (2H, m, H2, H6), 3.29-3.22 (2H, m, PP-C$\underline{H}_2$), 2.82-2.63 (2H, m, H2, H6), 2.04-1.94 (1H, m, H3), 1.83-1.54 (3H, m, H4, H5, H4), 1.29-1.19 (1H, m, H5); ^{13}C NMR (150 MHz, DMSO-d_6, room temperature): δ (ppm)= 117.69 – 107.97 (2C, $CH_2\underline{C}F_2\underline{C}F_2H$), 74.07 (1C, s, PP-$\underline{C}H_2O$), 67.69 (1C, t, $^2J_{CF}$ = 26.3Hz, $O\underline{C}H_2CF_2CF_2H$), 46.01 (1C, s, C6), 43.98 (1C, s, C2), 33.99 (1C, s, C3), 24.79 (1C, s, C4), 21.84 (1C, s, C5); ^{19}F NMR (564 MHz, DMSO-d_6, room temperature): δ (ppm)= -124.81 (2F, s, $CH_2C\underline{F}_2CF_2H$), -139.61 (2F, s, $CH_2CF_2C\underline{F}_2H$); FTIR υ(cm^{-1}): 2940, 2915, ($\upsilon_{pp\text{-}N\text{-}H}$), 2886, 2826 ($\upsilon_{pp\text{-}CH2}$), 1573 ($\upsilon_{pp\text{-}N\text{-}H\text{-}bend}$), 1132, 1096 ($\upsilon_{CF2}$).

1.5. Synthesis of tert-butyl 3-(propoxymethyl)piperidine-1-carboxylate (5)

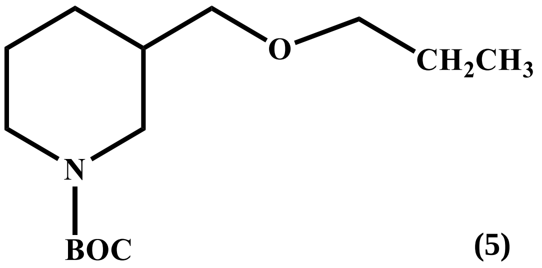

(5)

A magnetic stirrer and vacuum system were equipped in a 250 mL double-necked round-bottom flask. The air and moisture were pumped out of the flask using the vacuum system. To confirm the reaction system was in a nitrogen environment, this process was performed multiple times. $HCH_2CH_2CH_2OH$ (0.49g, 8.13 mmol) and CH_3ONa (2.43g, 13.54 mmol) were added into the two necked round bottom flask. Under the N_2 atmosphere, this mixture was then stirred for overnight. The following day, the methanol was removed under vacuum, and the reaction was driven to the product side leaving a white powder. Then 50 mL of freshly distilled anhydrous THF was added into the reaction flask under ice cooling in an external ice bath. Then, **2 (**1.0g, 2.71 mmol) was added into the reaction mixture and the mixture was stirred for overnight while gradually warming to room temperature. After confirming that the ligand was formed by using GC/MS analysis, the reaction was quenched by adding water. The entire content of the flask was then transferred to a 1 L separatory funnel and extracted three times with CH_2Cl_2 and deionized H_2O. The organic phase was then dried over Na_2SO_4 and concentrated under reduced pressure to give a colorless liquid of **5**.

Analytical data of (**5**): Yield: 70%; ^1H NMR (400 MHz, $CDCl_3$, room temperature δ(ppm)= 3.86-4.00 (2H m, H2, H6), 3.24-3.35 (4H, m, Ha, Hb), 2.53-2.82 (2 H, m, H2, H6), 1.72-1.80 (1H, m, H3), 1.51-1.66 (2H, m, H4), 1.43 (9H, s, *t*-butyl), 1.14-1.25 (3H, m, Hc, H5), 0.87-0.91 (4H, m, Hd, Hc); FTIR υ(cm^{-1}): 2963, 2929, 2854 ($\upsilon_{pp\text{-}CH2}$), 1693 ($\upsilon_{BOC\text{-}C=O}$); GC/MS (m/z): 16.2 min, 257 (M^+), 200 (M^+ - *t*-butyl), 184 (M^+ - *t*-butoxide), 156 (M^+ - BOC), 113 (M^+ - BOC - $CH_2CH_2CF_3$), 57 (*t*-butyl$^+$).

1.6. Synthesis of 3-(propoxymethyl)piperidinium iodide (5H-PPHI) (6)

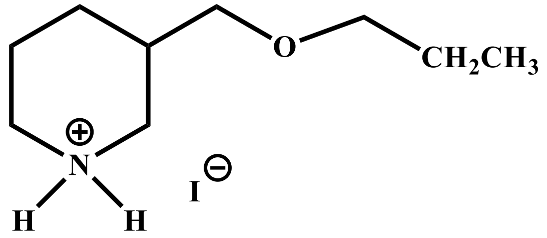

(6)

tert-butyl 3-(propoxymethyl)piperidine-1-carboxylate (Compound **5**) (0.25g, 1.0 mmol) was added to a 100 mL round-bottomed flask and 5 mL of isopropyl alcohol was added to dissolve **5**. To this was added 57 wt% hydroiodic acid (0.65g, 2.91 mmol) containing no stabilizer in an ice bath. The reaction mixture was stirred under N_2 (g) at 60 °C for 5 hr. Then the solvent was removed under vacuum, and the product was washed with a copious amount of diethyl ether and dried under vacuum for several days to give the white powder of **6** (also abbreviated as 5H-PPHI).

Analytical data of (**6**): Yield: 57.8%; m.p. 95-99 °C; ^{1}H NMR (400 MHz, DMSO-d_6, room temperature): δ(ppm)= 8.06-8.65 (2H PP-N$\underline{H_2}^+$), 3.29-3.22 (6 H, m, H2, H6, Ha), 2.82-2.53 (2 H, m, Hb), 2.08 (1 H, m, H3), 2.03-1.65 (2 H, m, Hc), 1.62-1.41 (3 H, m, H4, H5), 1.29-1.13 (1 H, m, H5), 0.90-0.78 (3 H, m, Hd); FTIR υ(cm^{-1}): 2942, 2935 ($\upsilon_{pp\text{-}N\text{-}H}$), 2868, 2834, 2801 ($\upsilon_{pp\text{-}CH2}$), 1573 ($\upsilon_{pp\text{-}N\text{-}H\text{-}bend}$).

2. Experimental and computational study

Electrochemistry: Electrochemical data were obtained by cyclic voltammetry (CV) using a using an electrochemical work station CH instrument (CHI-6041E) consisting of three electrodes where glassy carbon was used as the working electrode, Ag/AgCl (NaCl) was the reference electrode, and platinum wire was used as the counter electrode. The $(4FH\text{-}PPH)_2PbI_4$ HP material (0.01 M) was dissolved in acetone containing 0.1 M phosphate-buffered saline (PBS) and used as the electrolyte. These measurements were carried out at room temperature with a cyclic scan rate of 0.5 V s^{-1}. After the measurements, ferrocene was added as the internal reference for calibration.

The highest occupied molecular orbitals (HOMO) and the lowest unoccupied molecular orbitals (LUMO) of this fluorinated $(4FH\text{-}PPH)_2PbI_4$ HP were calculated by the CV curve and E_g with the equations[11] shown below:

$$E_{HOMO} = -[E_{ox} + 4.8] \quad \text{S1}$$

$$E_{LUMO} = E_{HOMO} + E_g \quad \text{S2}$$

where E_{ox}, which is the obvious oxidation potential from the ground state, is used to calculate HOMO [= -(4.8+0.6) eV] using Eq. S1; and for LUMO calculation: energy bandgap, E_g, is obtained from the Tauc plot (i.e. E_g= 2.22 eV). Then LUMO= -5.40 + 2.22= -3.18 (eV).

Computational methods: The geometry of 4FH-PPHI is optimized by the density functional theory (DFT)[12] at the B3LYP/6-311G(d)[13,14] level. All calculations are performed in the Gaussian 16 package.[15] and output files are visualized by its GaussView 6.0.16 program.[16] Electrostatic potential (ESP) calculations were completed to validate the formation of σ-hole region. The highest occupied molecular orbitals (HOMO) and the lowest unoccupied molecular orbitals (LUMO) were also computed for the 4FH-PPHI spacer.

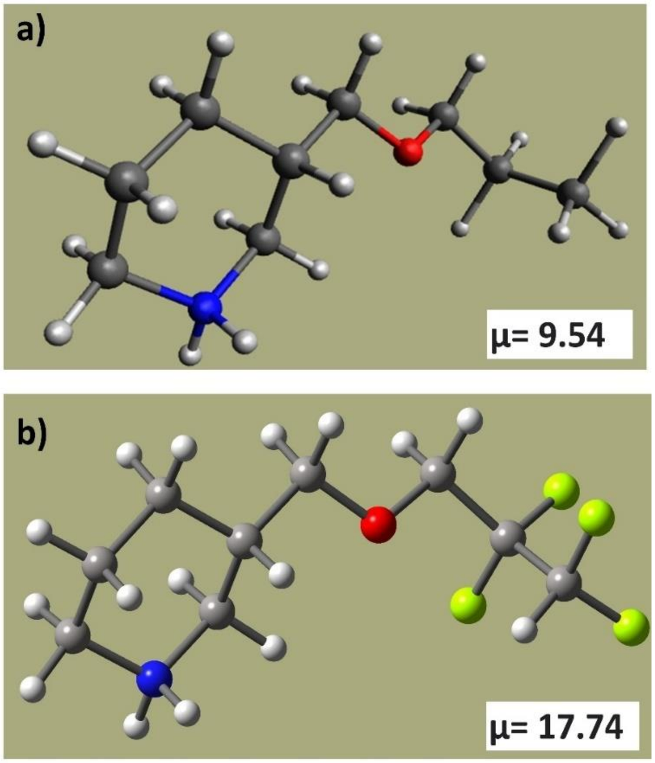

Figure S1. a) calculated molecular dipole moment of the non-fluorinated 4H-PPH$^+$ cation and b) the fluorinated 4FH-PPH$^+$ cation. Note 1: C, H, O, N, and F atoms are shown in grey, white, red, blue, and yellow colors, respectively. Note 2: the dipole moment of both fluorinated and non-fluorinated cations have been calculated using Gaussian16 with the B3LYP method and 6-311G(d,p) level of theory.

To further verify the element distribution and chemical states of the elements on the surface of film, we have conducted X-ray photoelectron spectroscopy (XPS) measurements on the fluorinated short-chained $(4FH\text{-}PPH)_2PbI_4$ 2D HP film shown below. Figure S2a shows the high-resolution C1s spectra of the prepared 2D HP film, while the most prominent peak, which is centered at 284.2 eV, represents the C–C bonds. The C1s spectra can be deconvoluted into the multiple distinct peaks, being assigned to C–O–C (285.2 eV)[17] and CF_2 (289.8 eV)[17], which confirm the successful preparation of fluorinated 4H-PPH$^+$ cation. As shown in Figure S2b, the XPS spectrum reveals a clear F 1s peak (687.1 eV)[18] for a fluorinated HP film as a result of the incorporation of the fluorinated side-chain (HCF_2CF_2) into the piperidinium (PP) cation. As shown

in Figure S2c, the fluorinated HP shows two characteristic peaks appearing at 142.5 and 137.6 eV for Pb 4f (of the Pb 4f core levels). As shown in Figure S2c, these two peaks are then assigned to Pb $4f_{5/2}$ and Pb $4f_{7/2}$, respectively. Similarly, as shown in Figure S2d, two prominent peaks for the I 3d core levels are located at 629.7 and 618.3 eV, which correspond to I $3d_{3/2}$ and I $3d_{5/2}$, respectively.[19-23] Therefore, these results have confirmed that the chemical states of all the elements, which are present in the fluorinated 2D HP, are consistent with its ideal chemical formula.

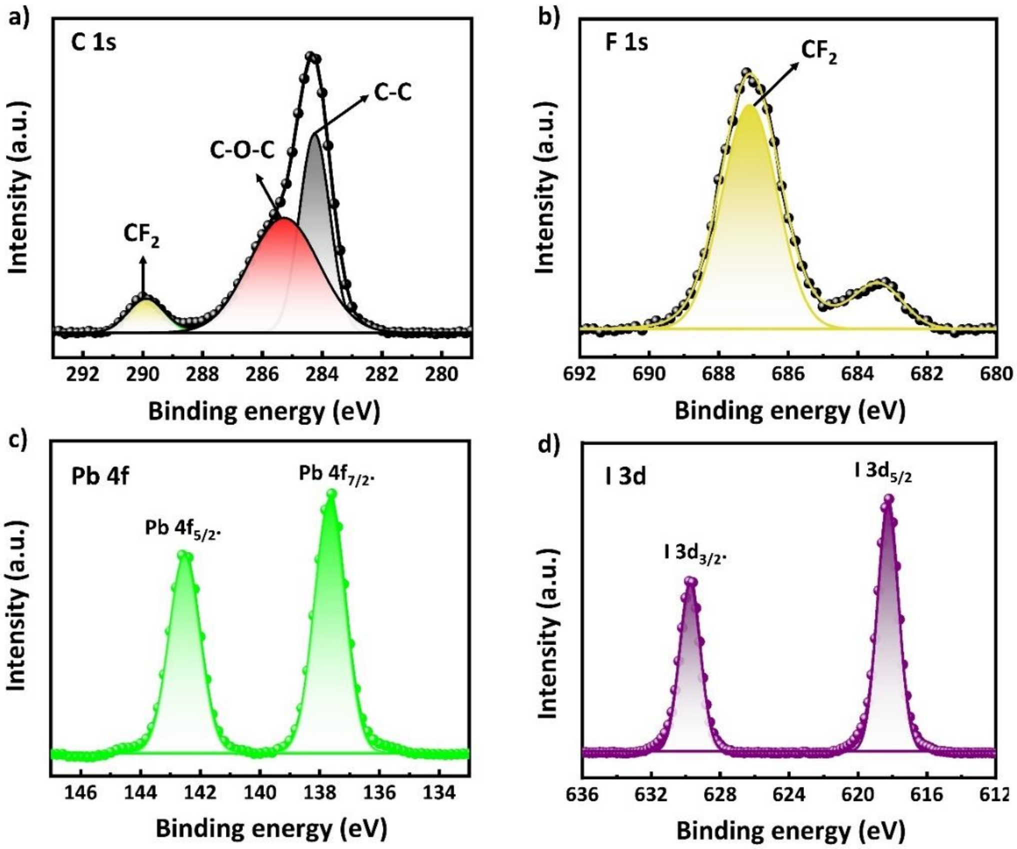

Figure S2. a) XPS spectra of C 1s, b) F 1s, c) Pb 4F, and d) I 3d fluorinated short-chained (4FH-PPH)$_2$PbI_4 HP film

3. Crystallization and structural data

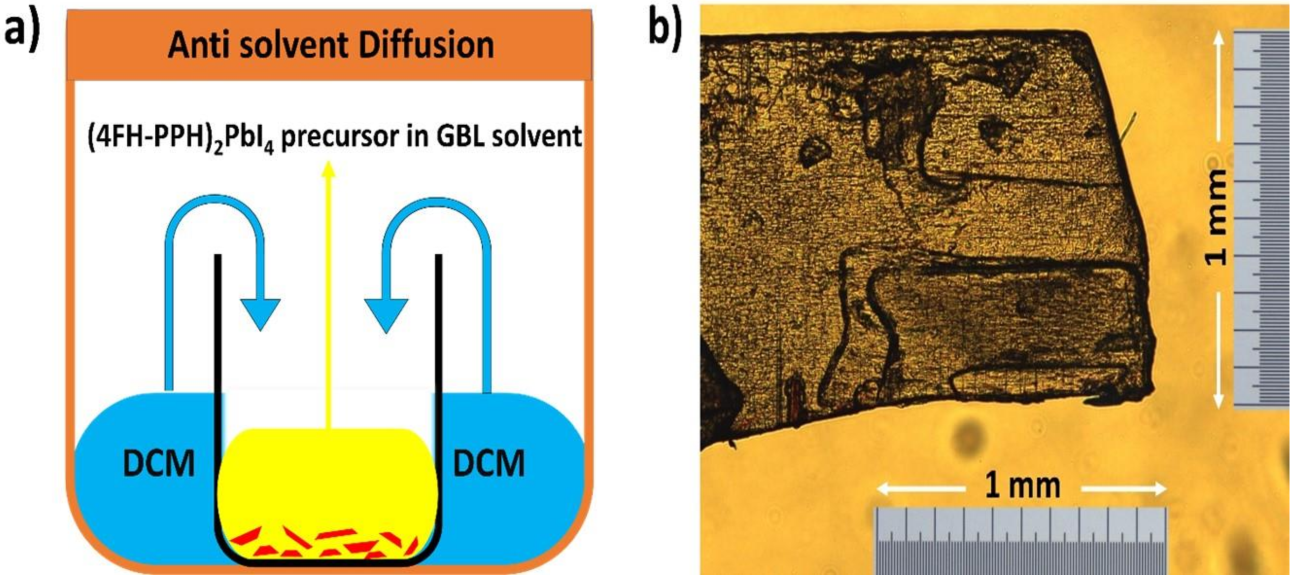

Figure S3. **a**) The schematic representation of crystallization by diffusion method; and b) optical microscope image of $(4FH\text{-}PPH)_2PbI_4$ HP crystal.

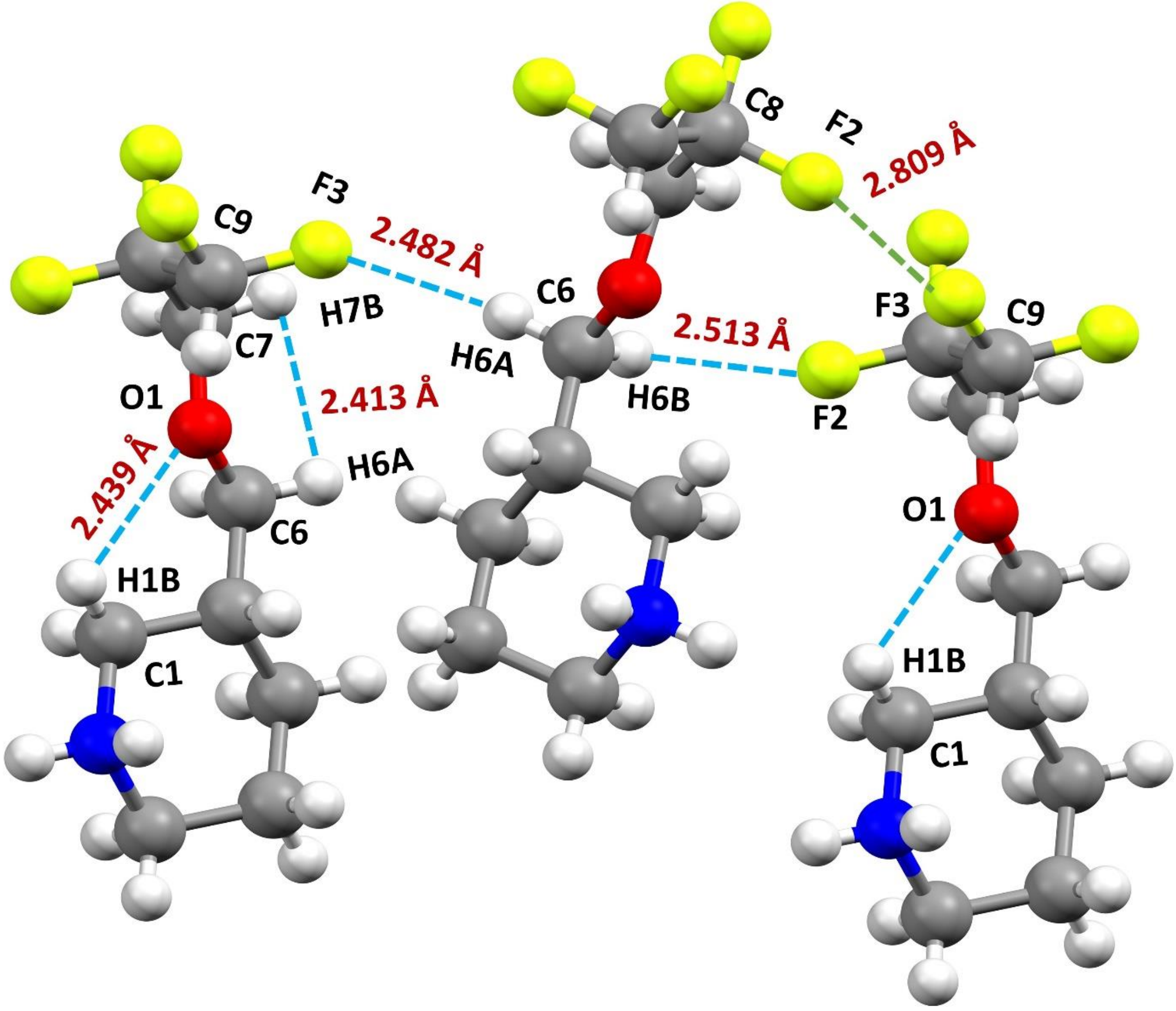

Figure S4. The C−H···Y(where Y= O and F), C−H···H−C improper (blue-dashed lines) hydrogen bonding, and C−F···F−C type-II (light green-dashed lines) halogen bonding (∠C8F2F3=165.7° & ∠C9F3F2=127.5°) interaction between 4FH-PPH cations.[24,25]

Stability monitoring

Powder X-ray diffraction (PXRD) and Fourier transform infrared spectra (FT-IR) studies.

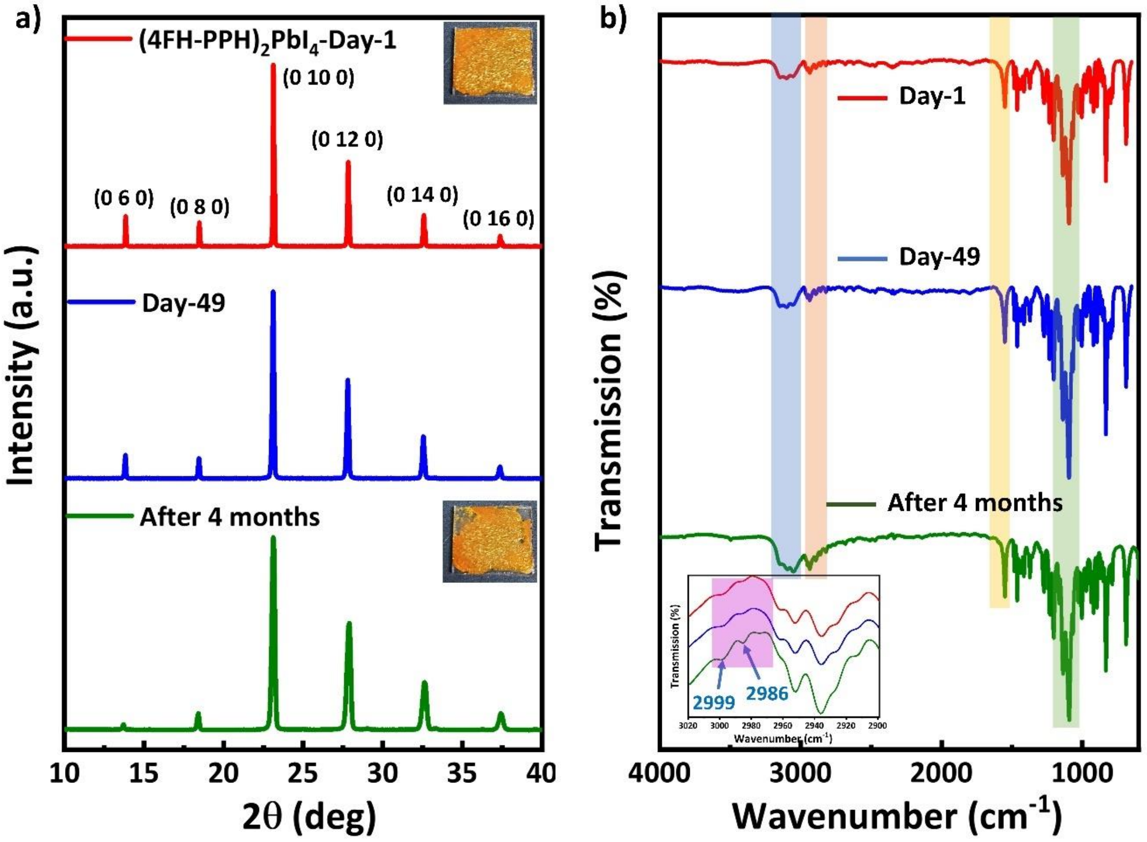

Figure S5. Powder X-ray diffraction (PXRD) spectra of fluorinated short-chained (4FH-PPH)$_2$PbI$_4$ HP film kept in air (without encapsulation) at room temperature (0V); then its stability has been monitored by being measured a) on day-1 (red color) , day-49 (blue color) and after 4 months(green color); b) FT-IR overlay spectra of fluorinated short-chained (4FH-PPH)$_2$PbI$_4$ HP film kept in air at room temperature (0 V); then its stability has been monitored by being measured on day-1(top; red trace), day-49 (middle; blue trace) and after 4 months (bottom; green trace). The enlarged view of the spectra between 3020-2900 cm^{-1} is shown in detail in the Figure S5b inset.

The blue-shifting frequencies for sp^3 C–H stretching due to improper H-bonding have been observed at 2986 and 2999 cm^{-1}. Note: In the FT-IR spectra the highlighted peaks from left to right represent N–H stretches (light blue), sp^3 C–H stretches (light orange), N–H bend (light yellow) and C–F stretches (light green), respectively. Note: the presence of blue-shifting frequencies for sp^3 C–H stretching after 4 months is also a good affirmation of the air stability of this fluorinated short-chained (4FH-PPH)$_2$PbI$_4$ HP film.

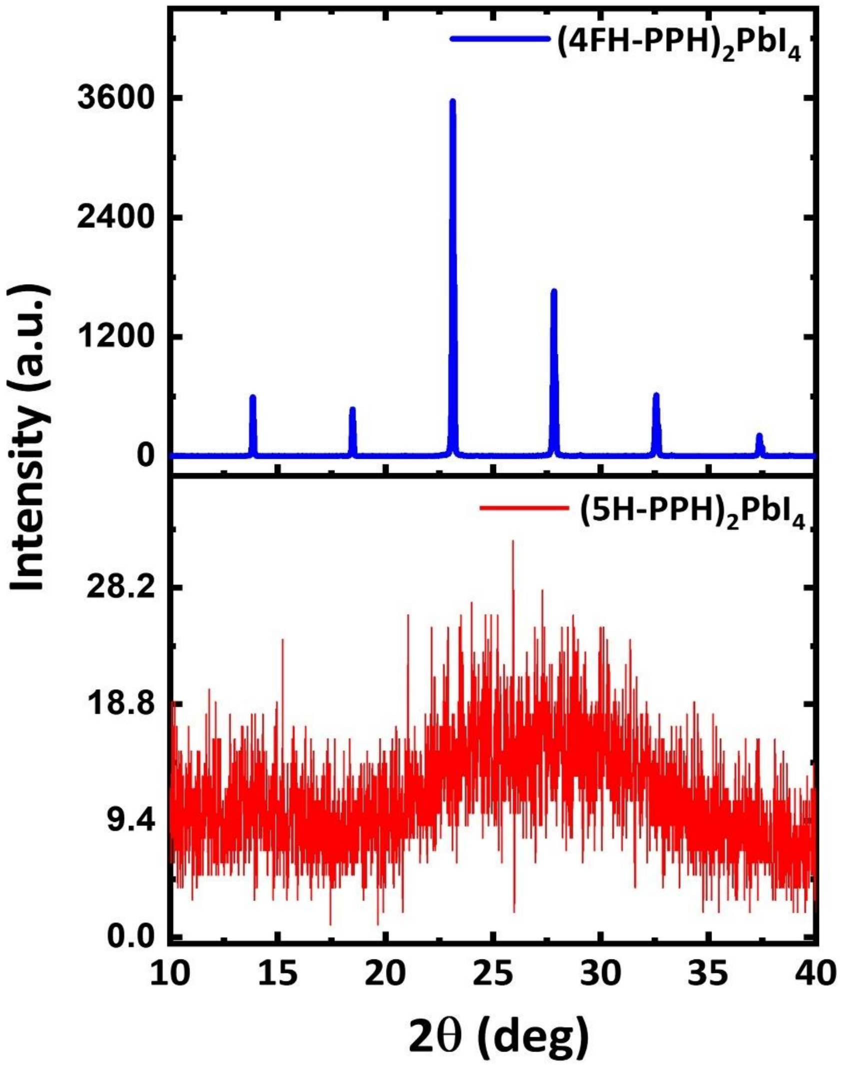

Figure S6. Comparison of PXRD spectra showing the fluorinated $(4FH\text{-}PPH)_2PbI_4$ (top; blue color) and non-fluorinated $(5H\text{-}PPH)_2PbI_4$ (bottom; red color) HP films.

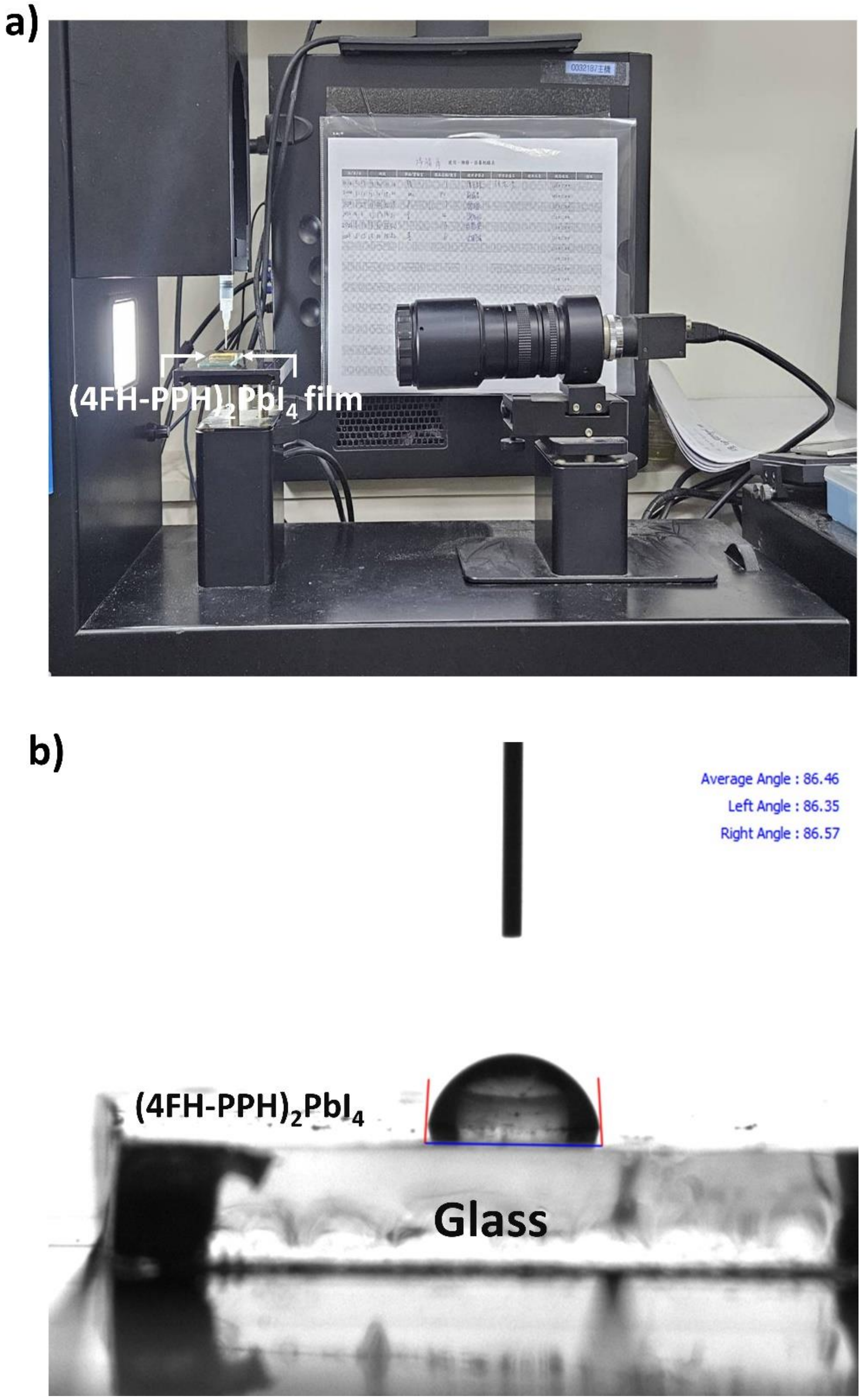

Figure S7. a) Photograph of the theta optical tensiometer and b) the contact angle measurement of a water droplet on fluorinated short-chained (4FH-PPH)$_2$PbI$_4$ HP film.[26]

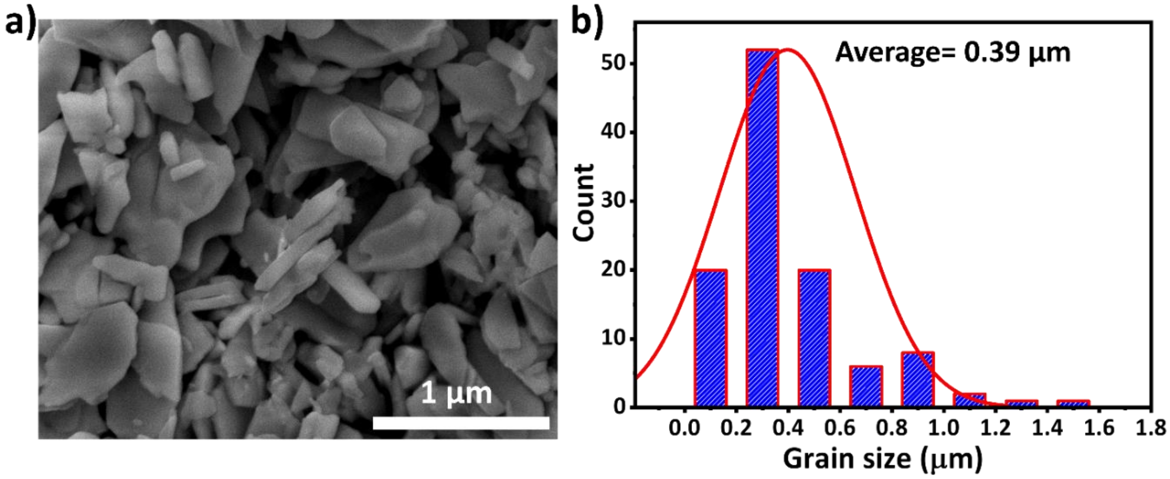

Figure S8. Grain size distribution analyses of fluorinated short-chained $(4FH\text{-}PPH)_2PbI_4$ HP powder (not a film). a) top view FESEM image and b) distributions of grain sizes for the perovskites with fitting lines.[27-29]

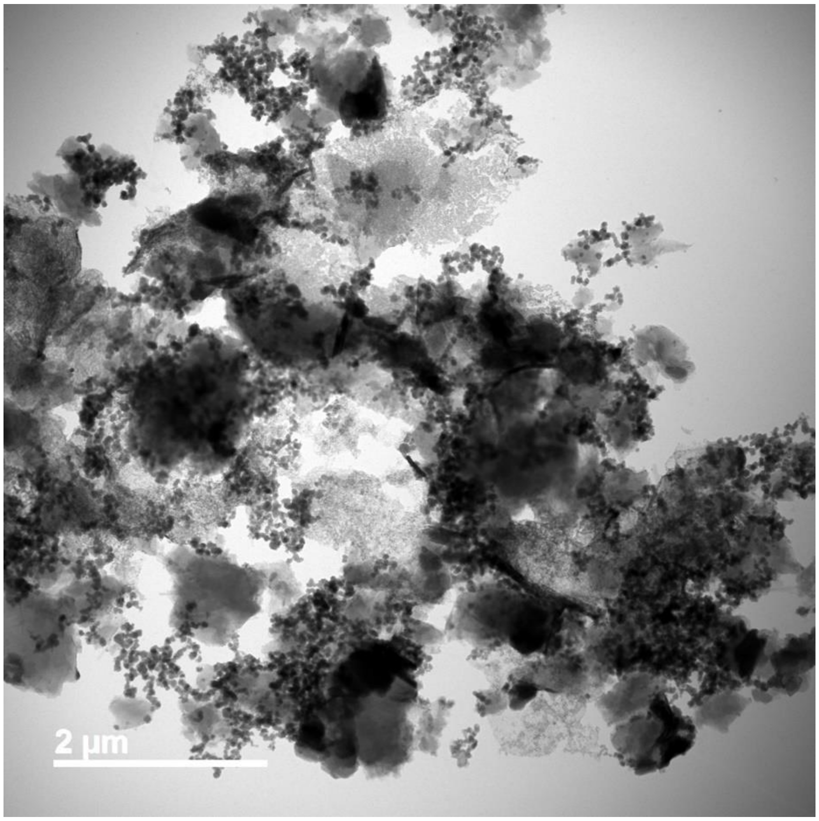

Figure S9. HR-TEM image (with a 2 µm scale bar) showing the selected area for EDS mapping of the fluorinated short-chained $(4FH\text{-}PPH)_2PbI_4$ HP. Note: The enlarged HR-TEM image from Figure S9 is shown in Figure 5a of the main article.

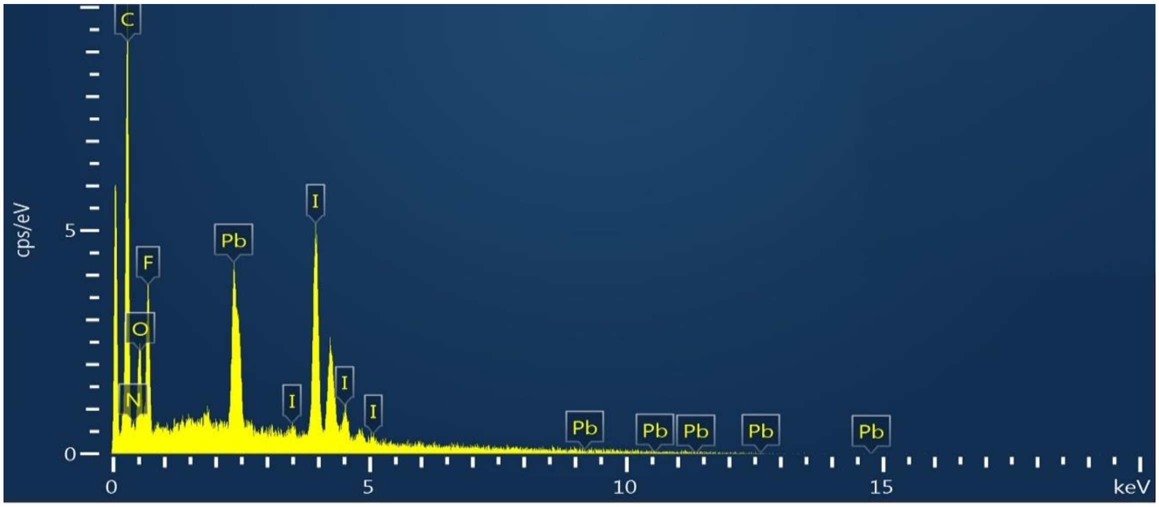

Figure S10. EDS spectrum of the fluorinated short-chained $(4FH\text{-}PPH)_2PbI_4$ HP film showing a uniform distribution of all elements (C, N, O, F, Pb and I).

In contrast, the $(5H\text{-}PPH)_2PbI_4$, (3-**HCH₂**CH₂CH₂OCH₂-PPH)₂PbI₄, perovskite film, prepared by using non-fluorinated piperidinium, does not show the crystalline form in the PXRD pattern, but has decomposed readily in air shown in Figure S11. As a result, the non-fluorinated HP, $(5H\text{-}PPH)_2PbI_4$, has decomposed during the HR-TEM inverstigation shown in Figure S11a. However, However, we have noticed the several crystalline areas in the HR-TEM image shown in Figure S11b, which are presumably due to the presence of decomposed side-product (PbI_2). The presence of decomposed PbI_2 has been further confirmed by the high-resolution TEM image shown in inset A of Figure S11b, which has allowed us to calculate the d-spacing value(s). Additionally, the FFT pattern has shown a double-hexagon pattern, which well matches with the PbI_2 FFT data reported in literature.[30] In addition, two corresponding d-spacings of 2.18 and 3.82 Å for the inner hexagon and the outer hexagon are consistent with two reported values (2.15, 3.81 Å), respectively.[30]

Furthermore, EDS-mapping clearly showed the presence of PbI_2 by highlighting both Pb and I elements when compared with other elements (e.g. C, N O, and F) in the mapping shown in Figure S11c. These results are in a good agreement with the fact that the PbI_2 is then considered as the stable inorganic species at room temperature. These results certainly help us to conclude that the PbI_2 formation is likely due to the decomposition during HR-TEM data collection which requires the high voltage. Thus, these results are able to affirm that the fluorinated side-chained HP can contribute to enhance the crystallinity of the fluorinated 2D perovskites when compared with that of its non-fluorinated short-chained analogue.

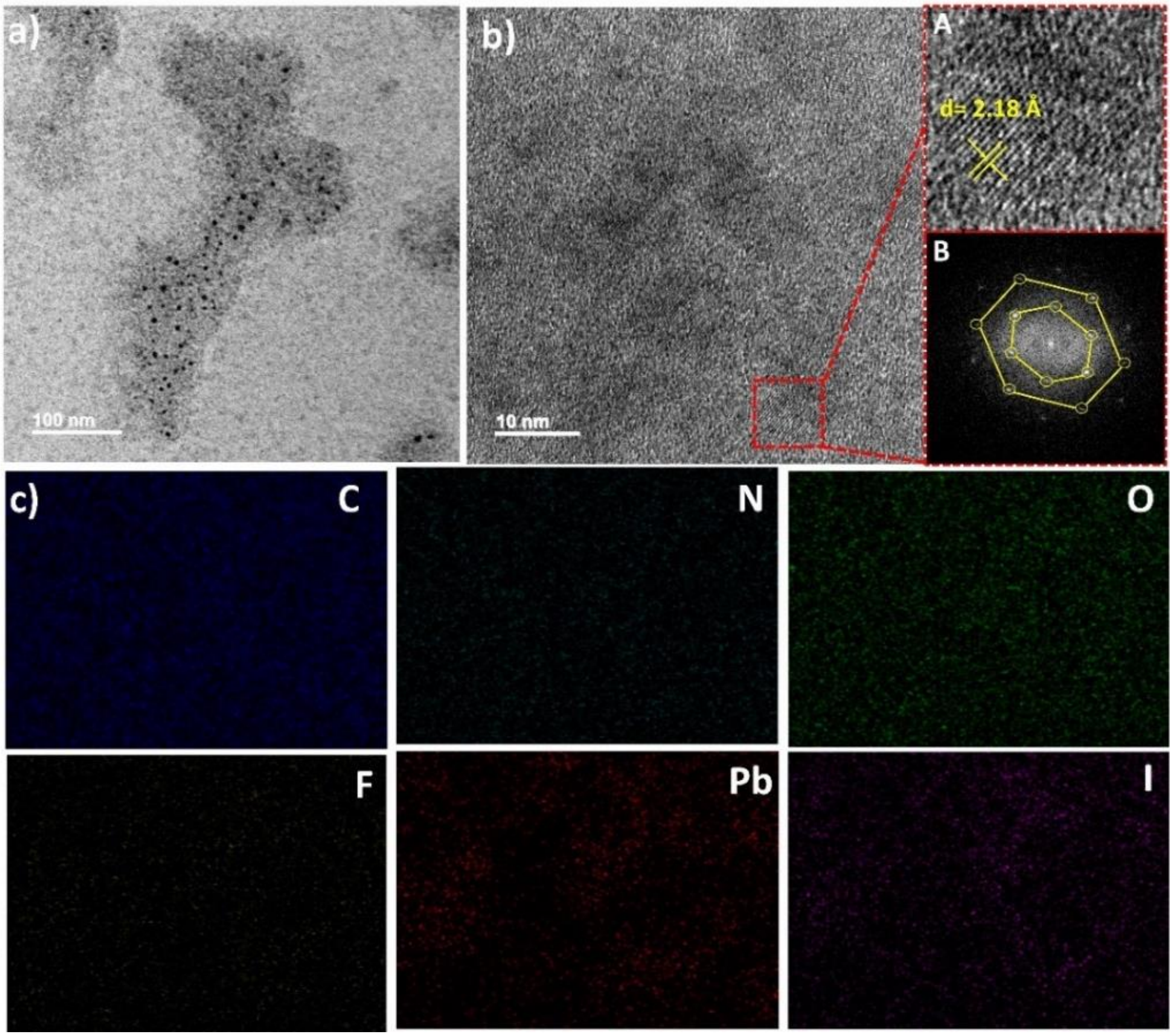

Figure S11. a,b) HR-TEM images and fast Fourier transform patterns of non-fluorinated short-chained $(5H\text{-}PPH)_2PbI_4$ HP and c) EDS-mapping of the non-fluorinated short-chained $(5H\text{-}PPH)_2PbI_4$ HP. Note 1: insets A and B of Figure 11b show an enlarged HR-TEM image and a corresponding FFT pattern of the non-fluorinated $(5H\text{-}PPH)_2PbI_4$ HP, respectively. Note 2: its EDS spectrum has not been recorded because of its readily decomposition in air during the HR-TEM measurement.

4. Energy band diagram and device scheme

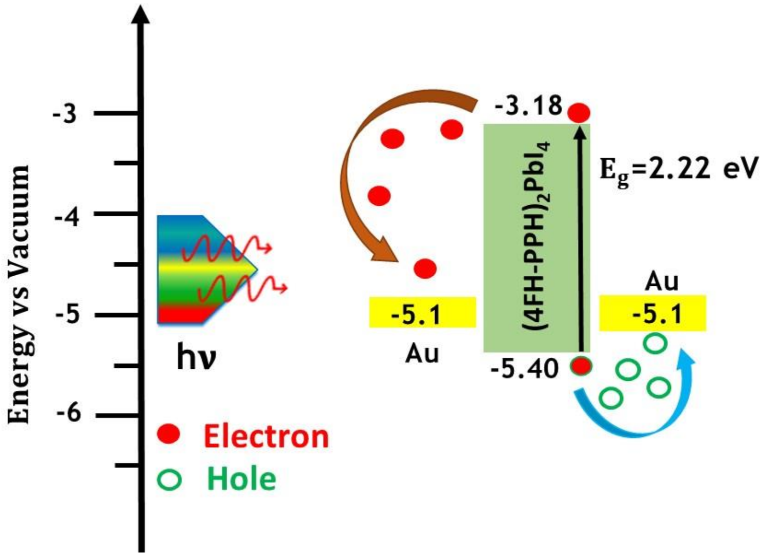

Figure S12. Schematic energy band diagram of the fluorinated short-chained $(4FH\text{-}PPH)_2PbI_4$ HP. Note: the calculations HOMO and LUMO values are shown in pp SI10.

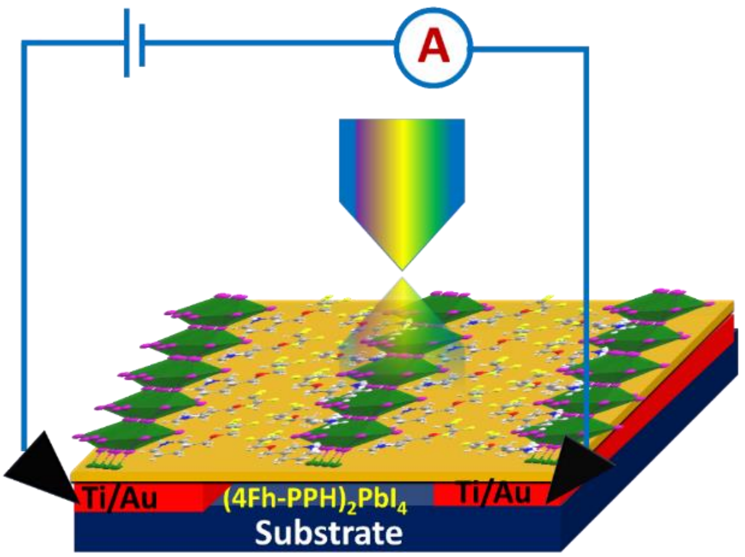

Figure S13. Schematic drawing of the fabricated device illustrating the fluorinated short-chained $(4FH\text{-}PPH)_2PbI_4$ HP photodetector (PD) on the SiO_2/Si substrate.[31-38]

Noise level of the fabricated photodetector device.

By sampling the dark current of the device and the source meter, we are able to perform power spectral density analysis[39] using the Welch method.[40] As shown in Figure S18, the noise current of the fluorinated short-chained $(4FH\text{-}PPH)_2PbI_4$ HP PD approaches 3 × 10^{-9} A/$Hz^{1/2}$ and 3 × 10^{-11} A/$Hz^{1/2}$ at low frequency and high frequency regions, respectively. Furthermore, we have statistically calculated the standard deviations of the noise level from photo-dark current of the fabricated photodetector device to be 1.14 × 10^{-10} and 0.80 × 10^{-10} A under 450 and 520 nm excitation, respectively. Meanwhile, the photocurrents of the fabricated photodetector device show values of 1.50 × 10^{-8} A and 1.21 × 10^{-8} A under 450 and 520 nm excitation, respectively. Thus, the signal-to-noise ratios of the photocurrent are 1.3 × 10^{2} and 1.5 × 10^{2}, respectively, showing two orders of magnitude difference between the signal and noise. Therefore, we believe that the presented fluorinated photodetector exhibits the significantly improved photocurrent when compared with the noise level.

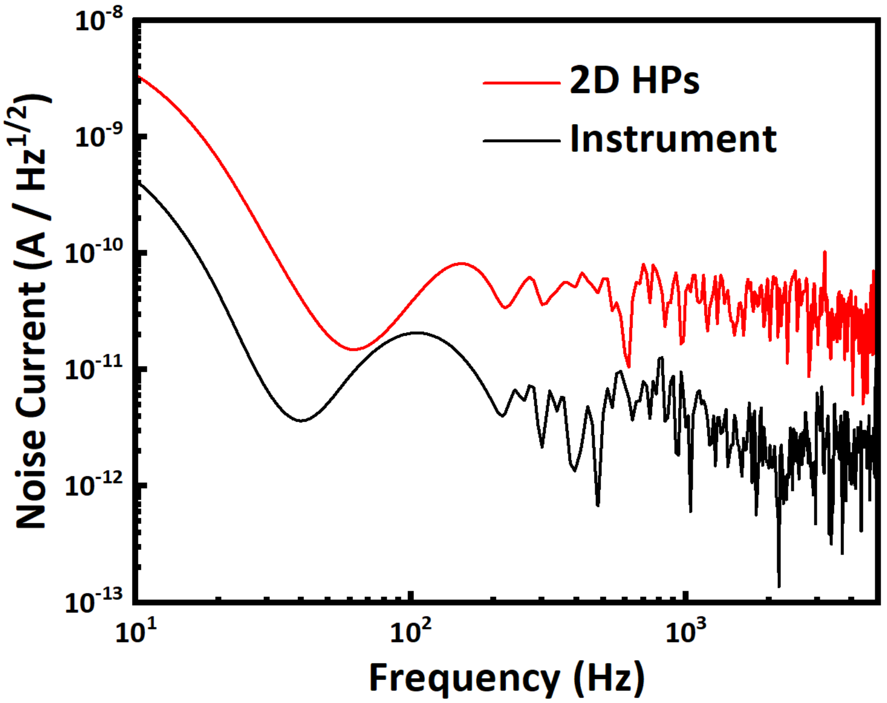

Figure S14. Noise current spectra of the fluorinated short-chained $(4FH\text{-}PPH)_2PbI_4$ HP PD.

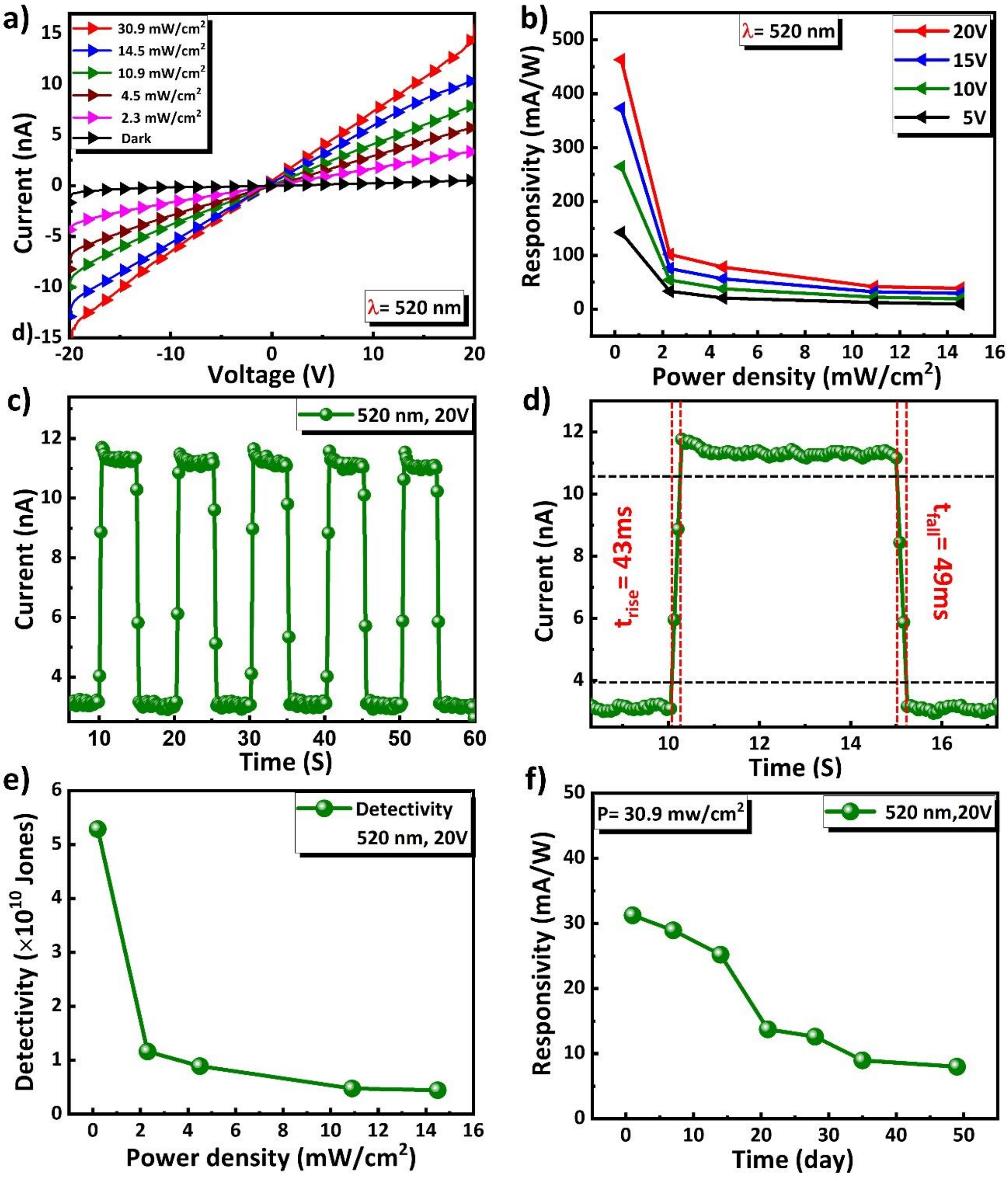

Figure S15. The photodetection performance of the fluorinated short-chained $(4FH\text{-}PPH)_2PbI_4$ HP PD device under 520 nm showing a) *I-V* characteristics under dark state and illumination as a function of power density, b) power density-dependent responsivity measured at the various biases from 5 to 20 V, c) time-dependent photocurrent response under 30.9 mW/cm^2, d) *I–t* curve in a cycle at 20 V, e) detectivity (*D*)* as a function of the power density, and f) responsivity of the PD device stored in air without any encapsulation for up to 49 days.

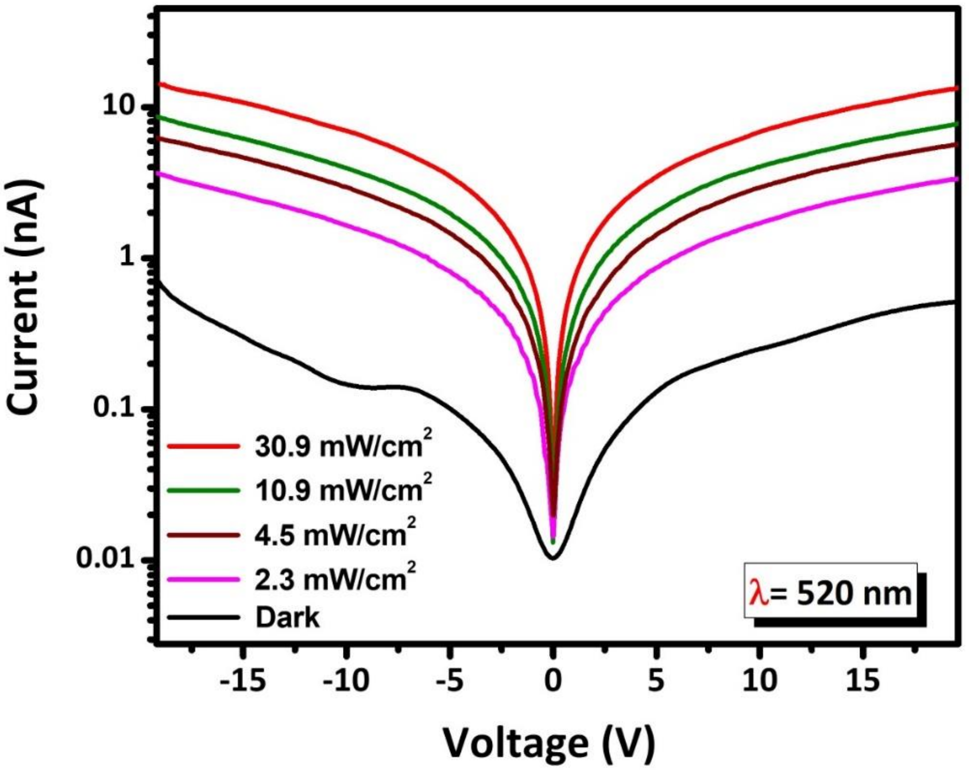

Figure S16. Semi-logarithmic *I-V* characteristics of the fluorinated short-chained $(4FH\text{-}PPH)_2PbI_4$ HP PD device under and under dark and upon light illumination of 520 nm at different power density at an applied ± 20 voltage.

5. Structural table section

Table S1. Selected bond lengths of fluorinated short-chained $(4FH\text{-}PPH)_2PbI_4$ HP (at 100 K).

**Bond**	**Bond length (Å)**	**Bond**	**Bond length (Å)**
Pb1—I1	3.195(1)	N1—H1C	0.890
Pb1—I1^{i}	3.195(1)	N1—H1D	0.890
Pb1—I2^{i}	3.198(1)	C1—H1A	0.970
Pb1—I2	3.198(1)	C1—H1B	0.970
Pb1—I2^{ii}	3.251(1)	C5—H5A	0.970
Pb1—I2^{iii}	3.251(1)	C5—H5B	0.970
C8—F1	1.354(13)	C6—H6B	0.970
C8— F2	1.353(14)	C6—H6A	0.970
C9— F3	1.349(16)	C9—H9	0.980
C9—F4	1.364(16)		

Symmetry codes: (i) -*x*+1, -*y*+1, *z*; (ii) *x*+1/2, -*y*+1, *z*-1/2; (iii) -*x*+1/2, *y*, *z*-1/2.

Table S2. Selected bond angles of fluorinated short-chained $(4FH\text{-}PPH)_2PbI_4$ HP (100 K).

**Bond**	**Bond angle (°)**
I1—Pb1—I1^{i}	177.96(3)
I1^{i}—Pb1—I2^{iii}	91.88(2)
I1^{i}—Pb1—I2^{ii}	86.85(1)
I1^{i}—Pb1—I2^{i}	88.98(2)
I1^{i}—Pb1—I2	92.54(2)
I1—Pb1—I2^{ii}	91.88(2)
I1—Pb1—I2^{i}	92.54(2)
I1—Pb1—I2	88.98(2)
I1—Pb1—I2^{iii}	86.85(1)
I2^{iii}—Pb1—I2^{ii}	102.67(2)
I2^{i}—Pb1—I2^{iii}	170.62(2)
I2—Pb1—I2^{i}	83.92(2)
I2—Pb1—I2^{iii}	86.71(1)
I2^{i}—Pb1—I2^{ii}	86.71(1)
I2—Pb1—I2^{ii}	170.62(2)
Pb1—I2—Pb1^{iv}	169.49(2)

Symmetry codes: (i) -*x*+1, -*y*+1, *z*; (ii) *x*+1/2, -*y*+1, *z*-1/2; (iii) -*x*+1/2, *y*, *z*-1/2; (iv) *x*-1/2, -*y*+1, *z*+1/2.

Table S3. N−H···I and C−H···X type of hydrogen bonds in fluorinated short-chained (4FH-PPH)$_2$PbI$_4$ HP (100 K).

Item	Hydrogen bond	D–H (Å)	H···A (Å)	D···A (Å)	<DHA (°)
1	N1–H1C···I1^{i}	0.89	2.83(1)	3.586(10)	144(1)
2	N1–H1C···I2^{ii}	0.89	3.12(2)	3.642(8)	119(2)
3	N1–H1D···I1	0.89	3.02(2)	3.692(9)	133(1)
4	C1–H1B···I1	0.97	3.16(2)	3.749(10)	120(2)
	C1–H1B···01	0.97	2.44(1)	2.798	101.4
5	C5–H5B···I1	0.97	3.17(2)	3.929(13)	136(1)
6	C6–H6A···F2^{iii}	0.97	2.51(1)	3.371(16)	147(1)
7	C6–H6B···F3^{iii}	0.97	2.48(1)	3.448(16)	174(1)

Symmetry codes: (i) 1/2-x, y, 1/2+z; (ii) x, y, z and (iii) 3/2-x, y, 1/2+z.

Table S4. Comparison of selected structural and optical properties of fluorinated short-chained $(4FH\text{-}PPH)_2PbI_4$ RP HP with other 2D RP HPs (different organic cations).

Item	Material	UV-Vis Bandgap (eV)	Pb−I−Pb Angle (°)	Ref.
1	$(PEA)_2PbI_4$	2.37	151.44<br>152.90	41
2	$(p\text{F PEA})_2PbI_4$	2.37	151.41	
3	$(o\text{F-PEA})_2PbI_4$	2.36	150.50	42
		2.62	148.7	
4	$(m\text{F-PEA})_2PbI_4$	2.35	151.00	
5	$(p\text{F-PEA})_2PbI_4$	2.32	152.98	
6	$(PMA)_2PbI_4$	2.32	157.52	43
7	$(p\text{F-PMA})_2PbI_4$	2.36	158.00	
8	$(CF_3\text{-PMA})_2PbI_4$	2.52	145.89	44
9	$(5FBzA)_2PbI_4$	2.52	149.73<br>150.46	45
10	$(EA)_2PbBr_4$	2.93	150.53	46
11	$(2FEA)_2PbBr_4$	3.04	144.03	
12	$(L_f)_2PbI_4$	2.6	144.73	47
13	**$(3F\text{-}PPH)_2PbI_2Br_2$**	**2.7**	**--**	48
**14**	**$(4FH\text{-}PPH)_2PbI_4$**	**2.22**	**169.49**	**This work**

Note 1. PEA- phenylethylammonium, xPEA- phenylethylammonium (x = ortho, meta and para), PMA-phenylmethylammonium, *x*PMA- phenylmethylammonium (*x* = CF_3), 5FBzA-pentafluorobenzyl ammonium, EA-ethylammonium, 2FEA-2,2-difluoroethylammonium and L_f- $C_9H_6F_{13}NH_3^+$.

From the results shown in this table, (except for data of this work) it has been found that the more the number of F atom added to the 2D perovskite materials, the smaller the Pb−I−Pb angle is. One F atom incorporation usually results in the Pb−I−Pb angle about 27 degrees smaller. And the incorporation of five F atoms (entry no. 9) results in the Pb−I−Pb angle being 30 degrees smaller to about 150°. It is also known that the distortion of the metal octahedra, which causes the Pb−I−Pb angle smaller due to the F atom incorporation, usually makes the energy bandgap (E_g) of the perovskite material to be increased. However, the results of current study show the opposite results in the Pb−I−Pb angle and the E_g. - i.e. by adding four F atoms into the organic spacer, its Pb−I−Pb angle is only ~10 degree smaller while Eg, of the fluorinated side-chained $(4FH\text{-}PPH)_2PbI_4$ HP is only 2.22 eV which is significantly smaller than those of other (n=1) fluorinated HP.

To investigates the fast charge transfer and charge carrier dynamics in a newly fabricated $(4FH-PPH)_2PbI_4$ fluorinated short-chained HP film, we have conducted time-resolved photoluminescence (TRPL) under a 405 nm laser to study the carrier dynamic information shown in Figure 6c and Table S5 below. We fit the TRPL curves using a biexponential reconvolution model (see equation S3 and S4 in SI). As shown in Figure 6c, the average carrier lifetime (τ_{ave}) of the fluorinated short-chained $(4FH-PPH)_2PbI_4$ HP film, is calculated to be ~0.323 ns (~0.761 ns using triexponential reconvolution model), with the carrier lifetime of ~15 ns. In contrast, the non-fluorinated HP film, $(5H-PPH)_2PbI_4$, which has almost no crystalline structure, does not show the photoluminescence (PL) and also lead to the rapid decomposition in air (see Figure S6 and S11). The detailed fitting parameters are summarized in Table S5. Additionally, the average carrier lifetime (~0.323 ns) of the fluorinated short-chained $(4FH-PPH)_2PbI_4$ HP film, is notably faster than those of other reported halide perovskites, such as those using butylammonium (BA) and fluorinated butylammonium (FBA).[49] The detailed comparisons of fluorous short-chained 2D $(4FH-PPH)_2PbI_4$ HP with other structurally similar HPs (BA, FBA, PEA and FPEA) from literature are described in Table S5. [Note: BA=butylammonium, FBA=4,4,4-trifluorobutylammonium, PEA=phenylethylammonium, and FPEA=4-fluorophenylethylammonium].[45] For example, as shown in items 1 and 2 (in Table S5), the average carrier lifetime (τ_{ave}) of BA is 236.9 ns, while the average carrier lifetime (τ_{ave}) of a fluorinated BA (FBA) shows the faster lifetime of 137.3 ns. Similarly, FPEA shows a faster lifetime of 129.4 ns than that (=313.8 ns) of its non-fluorinated analogue, PEA. These results affirm that incorporating the fluorine atom(s) into the organic spacer can significantly eliminates the non-radiative recombination ratio induced by trap centers, resulting in a fast charge transfer.[49-51] In fact, the reported fluorous short-chained $(4FH-PPH)_2PbI_4$ HP device, shows about two orders faster in the carrier lifetime (of ~0.323 ns) than those of FBA and FPEA-based HPs (see Table S5).

Table S5. Comparison of fitting parameters for time-resolved photoluminescence (TRPL) for the fluorinated short-chained $(4FH-PPH)_2PbI_4$ HP film with other reported HPs structures reported previously.

Item	Sample	τ_1 (ns)	A_1 (counts)	τ_2 (ns)	A_2 (counts)	$\tau_{(ave)}$ (ns)	Ref.
1	$(BA)_2PbI_4$	301	3429.6	57.3	6496.5	236.9	49
2	$(FBA)_2PbI_4$	166.8	2793.4	23.3	5155.2	137.3	
3	$(PEA)_2PbI_4$	372.1	3551.4	58.9	5137.1	313.8	
4	$(FPEA)_2PbI_4$	129.4	6020.6	27.5	4997.4	129.4	
**5**	**$(4FH-PPH)_2PbI_4$**	**0.078**	**7960**	**0.632**	**777**	**0.323**	**This work**

Note 1: BA-butylammonium, FBA-4,4,4-trifluorobutylammonium, PEA- phenylethylammonium, and FPEA-4-fluorophenylethylammonium. Note 2: the items 1-4 were used in the preparation of 2D/3D perovskites, which are applied in solar cell fabrication. Note 3: the τ_3 (3.28 ns), A_3 (59 counts) and $\tau_{(ave)}$ (0.761 ns) have been also calculated for the fluorous short-chained $(4FH-PPH)_2PbI_4$ HP using triexponential reconvolution model.

We fit the TRPL curves using a triexponential re-convolution model shown below:

$$I(t) = \int_{-\infty}^{t} IRF\,(t') \sum_{i=1}^{n} A_i \exp\left(-\frac{t-t'}{\tau_i}\right) dt' \qquad \text{(S3)}$$

where τ_{1}, τ_2 and τ_3 are the slow, intermediate and fast decay time, respectively; and A_1, A_2 and A_3 are their corresponding decay amplitudes. The average decay time has been calculated using the following equation (in S4):

$$\sigma_\tau = \frac{\sum_{i=1}^{n} A_i \tau_i^2}{\sum_{i=1}^{n} A_i \tau_i} \qquad \text{(S4)}$$

6. The NMR spectra (H-, F-, C-NMR) of prepared compounds

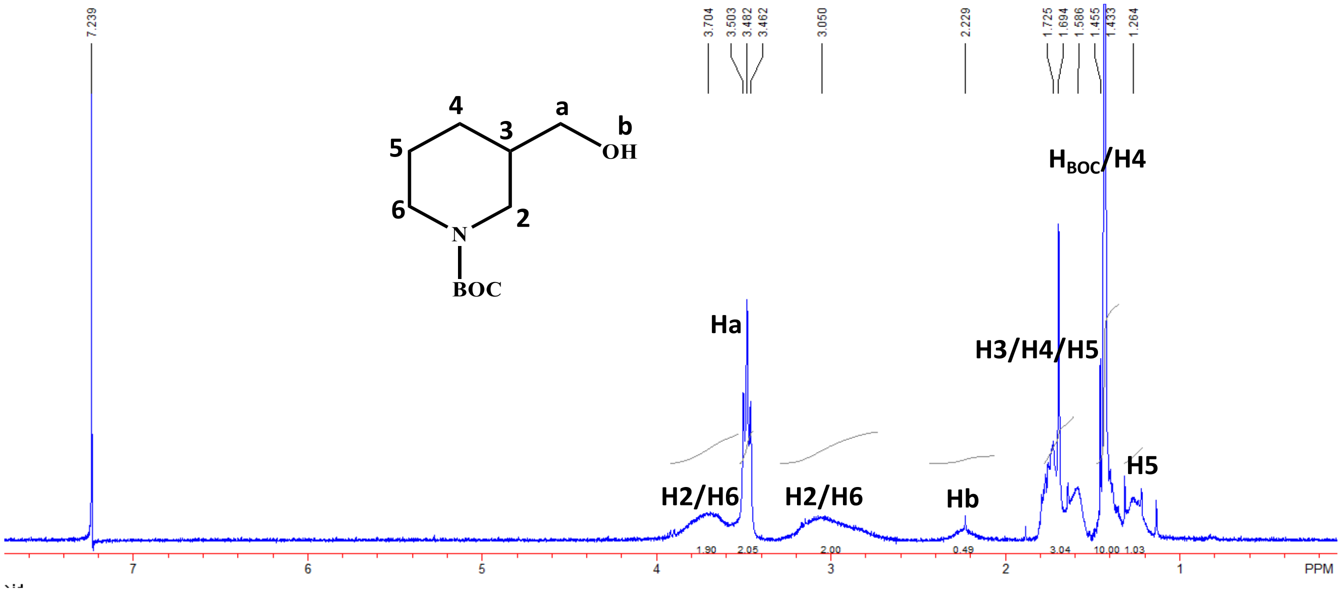

Figure S17. ^{1}H NMR spectrum of the compound (**1**) (in $CDCl_3$).

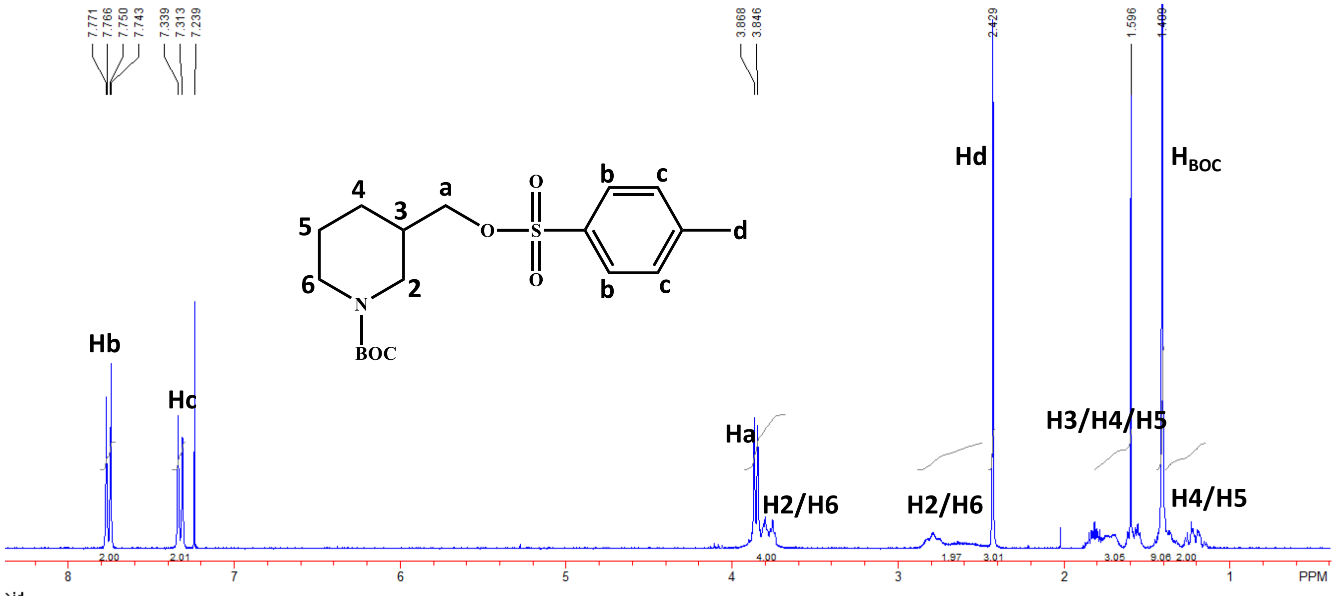

Figure S18. ^{1}H NMR spectrum of the compound (**2**) (in $CDCl_3$).

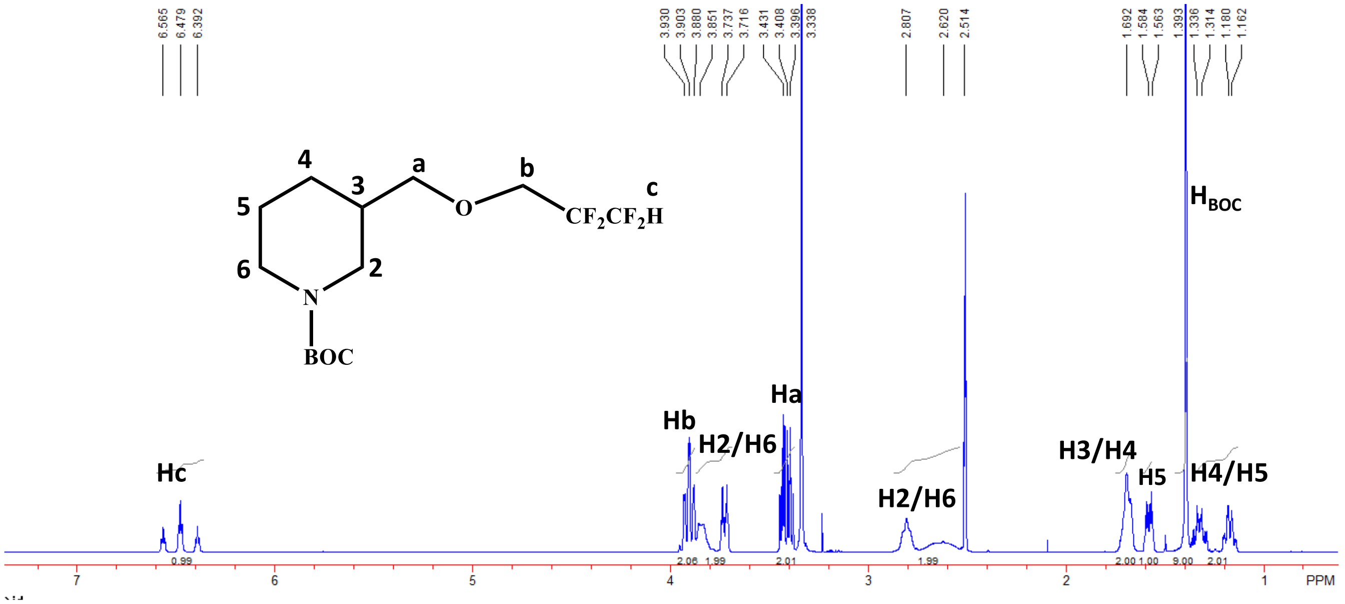

Figure S19. ^{1}H NMR spectrum of the compound (**3**) (in DMSO-d$_6$).

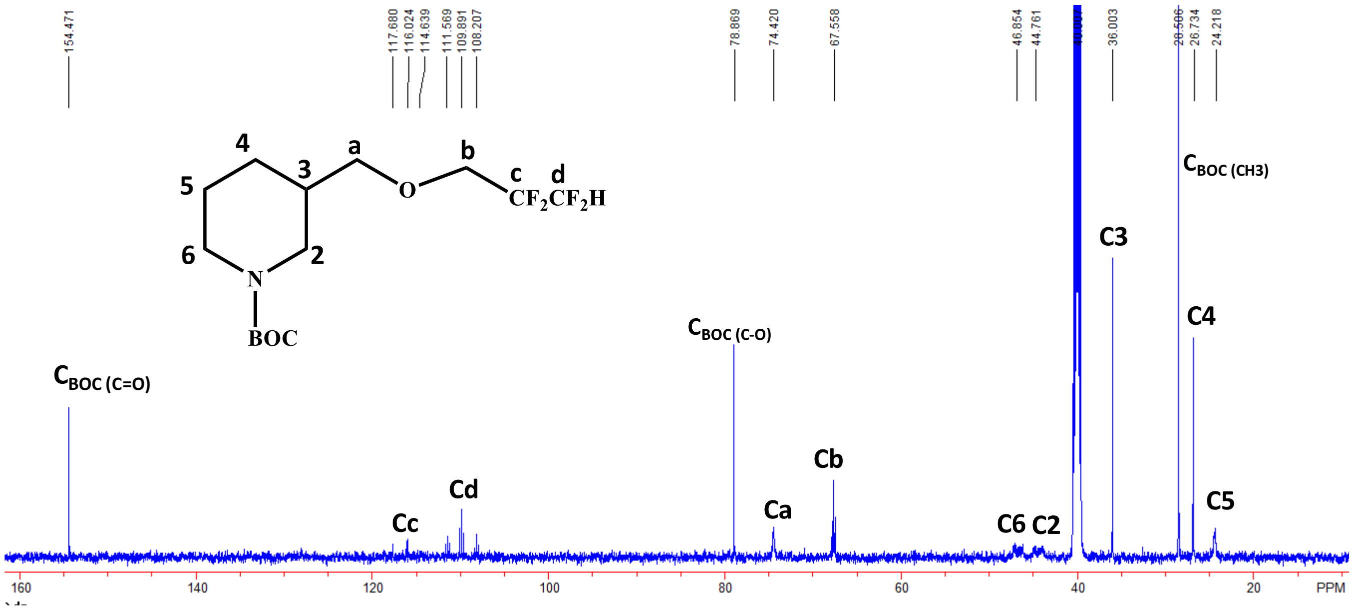

Figure S20. ^{13}C NMR spectrum of the compound (**3**) (in DMSO-d$_6$).

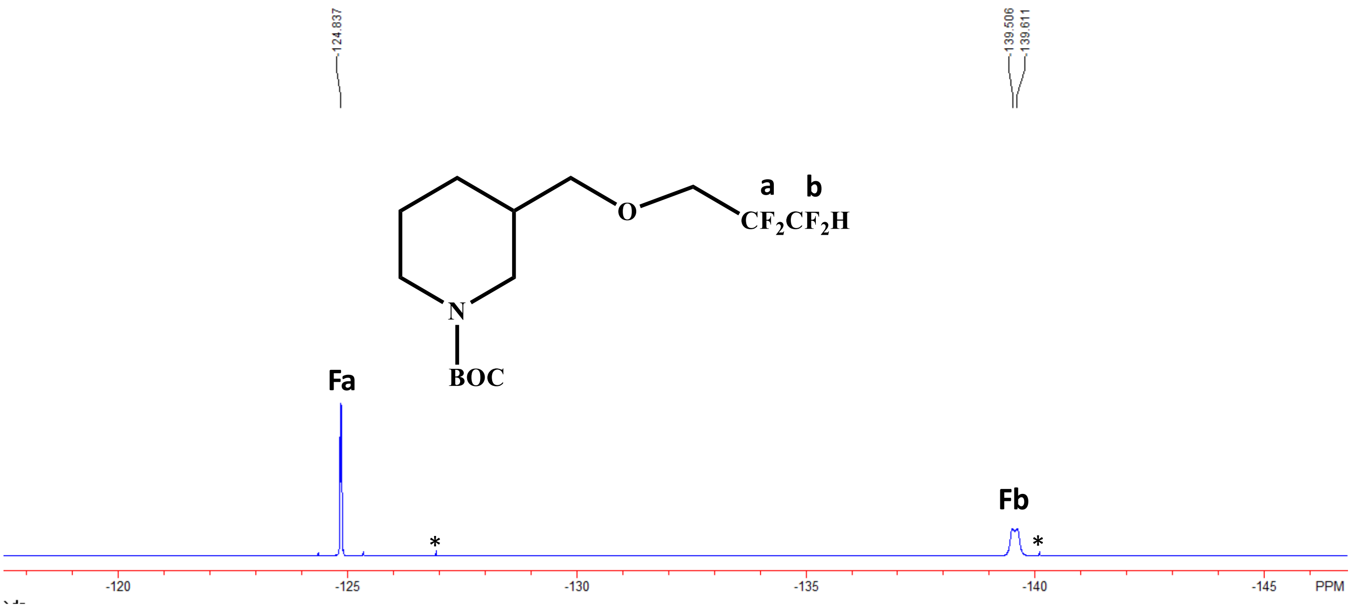

Figure S21. ^{19}F NMR spectrum of the compound (**3**) (in DMSO-d6).

(Note: * indicating the presence of residual 4FH alcohol, being used as a co-solvent.)

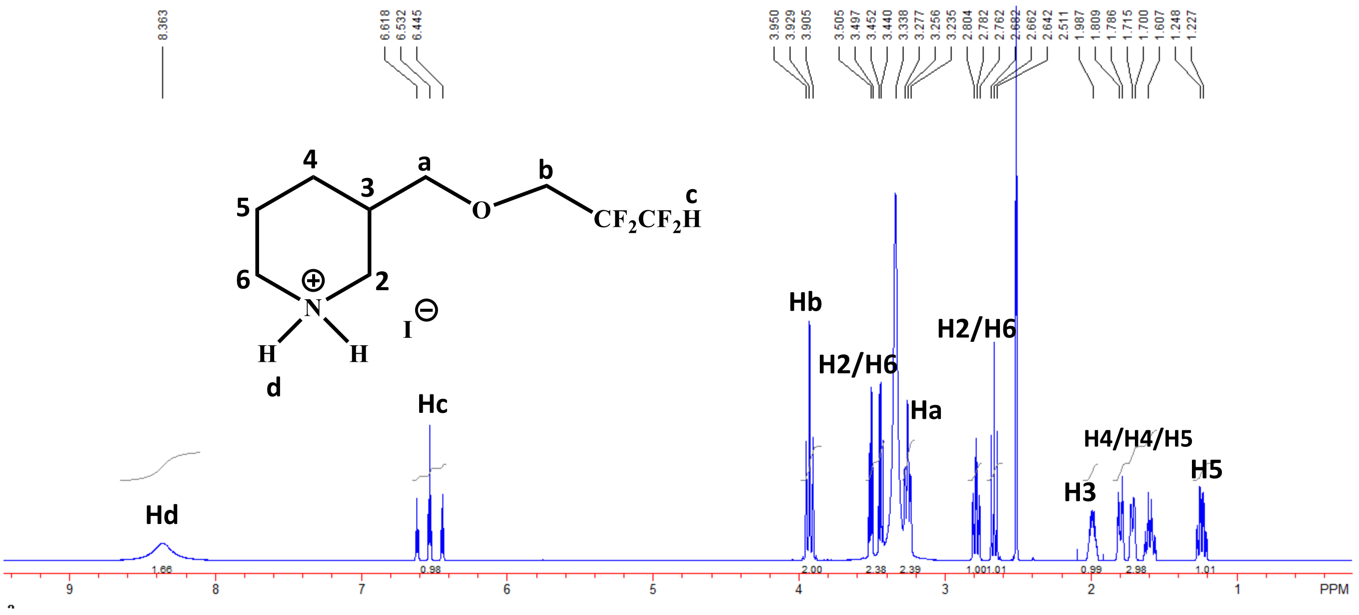

Figure S22. ^{1}H NMR Spectrum of the compound (**4**) (4FH-PPHI) (in DMSO-d6).

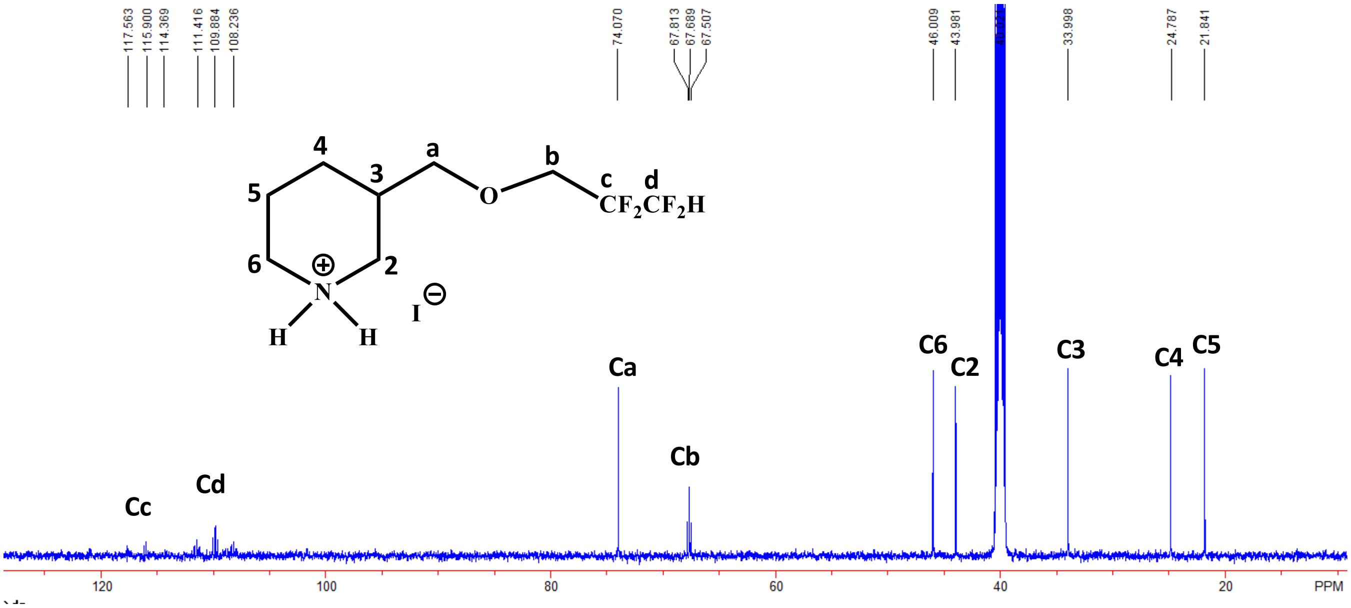

Figure S23. ^{13}C NMR spectrum of the compound (**4**) (4FH-PPHI) (in DMSO-d_6).

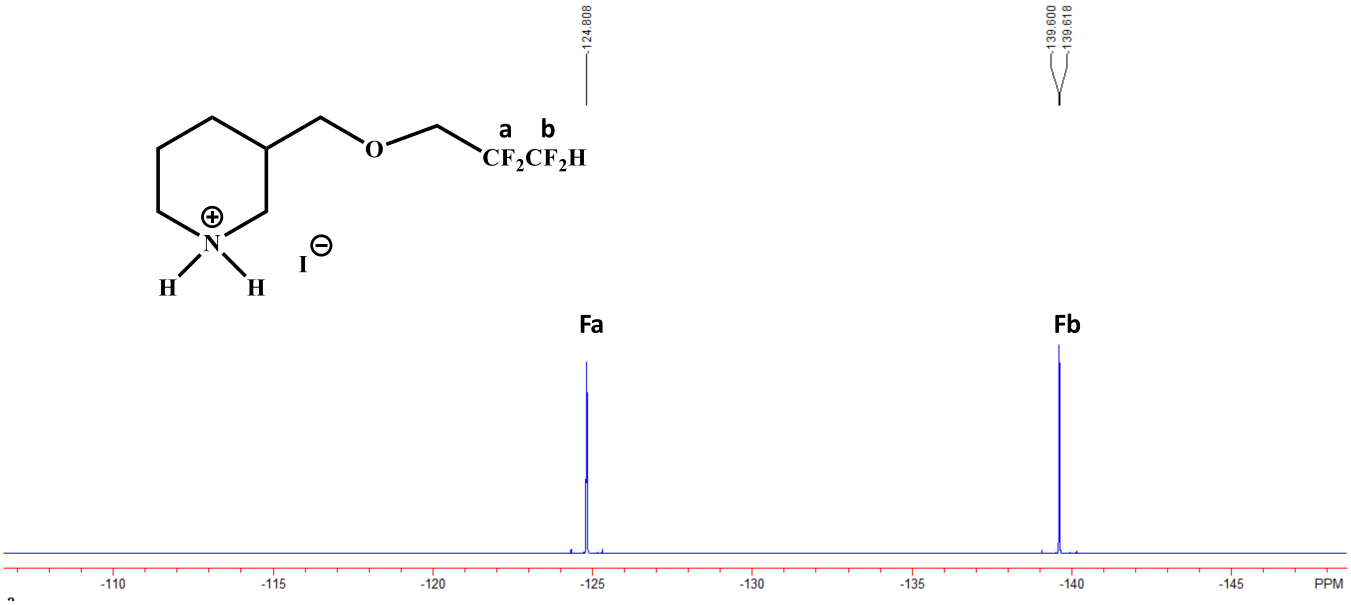

Figure S24. ^{19}F NMR spectrum of compound (**4**) (4FH-PPHI) (in DMSO-d_6).

References

(1) Pisani, L.; Farina, R.; Catto, M.; Iacobazzi, R. M.; Nicolotti, O.; Cellamare, S.; Mangiatordi, G. F.; Denora, N.; Soto-Otero, R.; Siragusa, L.; Altomare, C. D.; Carotti, A. Exploring Basic Tail Modifications of Coumarin-Based Dual Acetylcholinesterase-Monoamine Oxidase B Inhibitors: Identification of Water-Soluble, Brain-Permeant Neuroprotective Multitarget Agents. *J. Med. Chem.* **2016**, *59* (14), 6791–6806. https://doi.org/10.1021/acs.jmedchem.6b00562.

(2) Cioffi, C. L.; Muthuraman, P.; Raja, A.; Varadi, A.; Racz, B.; Petrukhin, K. Discovery of Bispecific Antagonists of Retinol Binding Protein 4 That Stabilize Transthyretin Tetramers: Scaffolding Hopping, Optimization, and Preclinical Pharmacological Evaluation as a Potential Therapy for Two Common Age-Related Comorbidities. *J. Med. Chem.* **2020**, *63* (19), 11054–11084. https://doi.org/10.1021/acs.jmedchem.0c00996.

(3) Lu, N.; Lin, Y.-C.; Chen, J.-Y.; Fan, C.-W.; Liu, L.-K. New Bis(Fluoro-Ponytailed) Bipyridine Ligands for Pd-Catalyzed Heck Reactions under Fluorous Biphasic Catalysis Condition. *Tetrahedron* **2007**, *63* (9), 2019–2023. https://doi.org/10.1016/j.tet.2006.12.053.

(4) Howell, J. L.; Lu, N.; Friesen, C. M. New Derivatives of Poly-Hexafluoropropylene Oxide from the Corresponding Alcohol. *J. Fluor. Chem.* **2005**, *126* (3), 281–288. https://doi.org/10.1016/j.jfluchem.2004.09.020.

(5) Lu, N.; Chen, J.; Fan, C.; Lin, Y.; Wen, Y.; Liu, L. (2,2′-Bipyridine)Palladiumdichloride Derivatives as Recyclable Catalysts in Heck Reactions. *J. Chinese Chem. Soc.* **2006**, *53* (6), 1517–1521. https://doi.org/10.1002/jccs.200600198.

(6) Lu, N.; Ou, Y.-M.; Feng, T.-Y.; Cheng, W.-J.; Tu, W.-H.; Su, H.-C.; Wang, X.; Liu, L.; Hennek, M. D.; Sayler, T. S.; Thrasher, J. S. Synthesis and Characterization of Polyfluorinated 2,2′-Bipyridines and Their Palladium and Platinum Complexes, [$MX_2(Bis(R_fCH_2OCH_2)$-2,2′-Bpy)] (X=Cl, Br). *J. Fluor. Chem.* **2012**, *137*, 54–63. https://doi.org/10.1016/j.jfluchem.2012.02.009.

(7) Lu, N.; Lin, K.-Y.; Li, C.-K.; Kung, C.-C.; Yeh, Y.-P.; Cheng, Y.-Y.; Liu, L.-K. Recyclable Palladium Catalysts for the Heck/Sonogashira Reaction under Microwave-assisted Thermomorphic Conditions. *J. Chinese Chem. Soc.* **2015**, *62* (1), 64–72. https://doi.org/10.1002/jccs.201400252.

(8) Lu, N.; Tu, W.-H.; Hou, H.-C.; Lin, C.-T.; Li, C.-K.; Liu, L.-K. Synthesis, Structure and Spectroelectrochemical Property of (2,2′-Bipyridine)–Metal (M=Pt, Pd) Dichloride with 4,4′-Bis(Fluorous-Ponytail) on Bipyridine. *Polyhedron* **2010**, *29* (3), 1123–1129. https://doi.org/10.1016/j.poly.2009.12.015.

(9) Elakkat, V.; Chang, C.-C.; Chen, J.-Y.; Fang, Y.-C.; Shen, C.-R.; Liu, L.-K.; Lu, N. The First Two Examples of Halogen Bonding with a Sigma Hole-Donating Fluorine in the C Sp^3–F⋯O Sp^3 Interaction from Polyfluorinated Trans -Dihalo-Palladium(II) Di-Substituted Pyridine Complexes. *Chem. Commun.* **2019**, *55* (95), 14259–14262. https://doi.org/10.1039/C9CC06731K.

(10) Mao, L.; Ke, W.; Pedesseau, L.; Wu, Y.; Katan, C.; Even, J.; Wasielewski, M. R.; Stoumpos,

C. C.; Kanatzidis, M. G. Hybrid Dion–Jacobson 2D Lead Iodide Perovskites. *J. Am. Chem. Soc.* **2018**, *140* (10), 3775–3783. https://doi.org/10.1021/jacs.8b00542.
(11) Pathak, A.; Chhoker, S.; Singh, P.; Sharma, H.; Kumar, R. Electrochemical studies of halide perovskite and its correlation for photocatalytic applications. *Solid State Sci.* **2023**, *139* (1) 107159. https://doi.org/10.1016/j.solidstatesciences.2023.107159.
(12) Runge, E.; Gross, E. K. U. Density-Functional Theory for Time-Dependent Systems. *Phys. Rev. Lett.* **1984**, *52* (12), 997–1000. https://doi.org/10.1103/PhysRevLett.52.997.
(13) Becke, A. D. Density-functional Thermochemistry. I. The Effect of the Exchange-only Gradient Correction. *J. Chem. Phys.* **1992**, *96* (3), 2155–2160. https://doi.org/10.1063/1.462066.
(14) Hariharan, P. C.; Pople, J. A. Accuracy of AH n Equilibrium Geometries by Single Determinant Molecular Orbital Theory. *Mol. Phys.* **1974**, *27* (1), 209–214. https://doi.org/10.1080/00268977400100171.
(15) Frisch, M.E., Trucks, G.W., Schlegel, H.B., Scuseria, G.E., Robb, M.A., Cheeseman, J.R., Scalmani, G., Barone, V.P.G.A., Petersson, G.A., Nakatsuji, H.J.R.A. and Li, X. **2016**, Gaussian 16, revision C. 01.
(16) Dennington, R.; Keith, T.; Millam, J. **2009**, GaussView, version 5.
(17) Tingting, M.E.I.; Ming, G.A.O.; Danni, L.I.U.; Yu, W.A.N.G; Huang, Y. Enhanced electrocatalytic activity of carbon cloth by synergetic effect of plasma and acid treatment. *Plasma* Sci. Technol. **2021**, *23* (2), 025504. https://doi.org/10.1088/2058-6272/abd8b4.
(18) Xu, D.; Wang, J.; Duan, Y.; Yang, S.; Zou, H.; Yang, L.; Zhang, N.; Zhou, H.; Lei, X.; Wu, M.; Liu, S. Highly-Stable $CsPbI_3$ Perovskite Solar Cells with an Efficiency of 21.11% via Fluorinated 4-Amino-Benzoate Cesium Bifacial Passivation. *Adv. Funct. Mater.* **2023**, *33* (44), 2304237. https://doi.org/10.1002/adfm.202304237.
(19) Zhang, Y.; Chen, M.; He, T.; Chen, H.; Zhang, Z.; Wang, H.; Lu, H.; Ling, Q.; Hu, Z.; Liu, Y.; Chen, Y.; Long, G. Highly Efficient and Stable FA-Based Quasi-2D Ruddlesden–Popper Perovskite Solar Cells by the Incorporation of Β-Fluorophenylethanamine Cations. *Adv. Mater.* **2023**, *35* (17), 2210836. https://doi.org/10.1002/adma.202210836.
(20) Gong, X.; Li, H.; Zhou, R.; Peng, X.; Ouyang, Y.; Luo, H.; Liu, X.; Zhuang, J.; Wang, H.; Ni, Y.; Lei, Y. Strong electron acceptor of a fluorine-containing group leads to high performance of perovskite solar cells. *ACS Appl. Mater. Interfaces*, **2021**, *13* (34), 41149-41158. https://doi.org/10.1021/acsami.1c07610.
(21) Bati, A.S.; Jiang, W.; Chu, R.; Mallo, N.; Burn, P.L.; Gentle, I.R; Shaw, P.E. Fluorinated Cation-Based 2D Perovskites for Efficient and Stable 3D/2D Heterojunction Perovskite Solar Cells. *ACS Appl. Mater. Interfaces*, **2023**, *15* (49), 57835-57845. https://doi.org/10.1021/acsami.3c13609.
(22) Lai, H.; Lu, D.; Xu, Z.; Zheng, N.; Xie, Z.; Liu, Y. Organic-salt-assisted crystal growth and orientation of quasi-2D Ruddlesden–Popper perovskites for solar cells with efficiency over 19%. *Adv. Mater.* **2020**, *32* (33), 2001470. https://doi.org/10.1002/adma.202001470.
(23) Wang, R.; Dong, X.; Ling, Q.; Fu, Q.; Hu, Z.; Xu, Z.; Zhang, H.; Li, Q.; Liu, Y. Spacer

engineering for 2D ruddlesden–popper perovskites with an ultralong carrier lifetime of over 18 μs enable efficient solar cells. **2022**, *ACS Energy Lett.* *7* (10), 3656-3665. https://doi.org/10.1021/acsenergylett.2c01800.
(24) Cavallo, G.; Metrangolo, P.; Milani, R.; Pilati, T.; Priimagi, A.; Resnati, G.; Terraneo, G. The Halogen Bond. *Chem. Rev.* **2016**, *116* (4), 2478–2601. https://doi.org/10.1021/acs.chemrev.5b00484.
(25) Elakkat, V.; Tessema, E.; Lin, C.; Wang, X.; Chang, H.; Zheng, Y.; Huang, Y.; Gurumallappa; Zhang, Z.; Long Chan, K.; Rahayu, H. A.; Francisco, J. S.; Lu, N. Unusual Changes of C−H Bond Lengths in Chiral Zinc Complexes Induced by Noncovalent Interactions. *Angew. Chemie Int. Ed.* **2023**, *62* (6), e202215438. https://doi.org/10.1002/anie.202215438.
(26) Bati, A. S. R.; Jiang, W.; Chu, R.; Mallo, N.; Burn, P. L.; Gentle, I. R.; Shaw, P. E. Fluorinated Cation-Based 2D Perovskites for Efficient and Stable 3D/2D Heterojunction Perovskite Solar Cells. *ACS Appl. Mater. Interfaces* **2023**, *15*, 57845. https://doi.org/10.1021/acsami.3c13609.
(27) Zhang, F.; Kim, D. H.; Lu, H.; Park, J.-S.; Larson, B. W.; Hu, J.; Gao, L.; Xiao, C.; Reid, O. G.; Chen, X.; Zhao, Q.; Ndione, P. F.; Berry, J. J.; You, W.; Walsh, A.; Beard, M. C.; Zhu, K. Enhanced Charge Transport in 2D Perovskites via Fluorination of Organic Cation. *J. Am. Chem. Soc.* **2019**, *141* (14), 5972–5979. https://doi.org/10.1021/jacs.9b00972.
(28) Lai, X.; Li, W.; Gu, X.; Chen, H.; Zhang, Y.; Li, G.; Zhang, R.; Fan, D.; He, F.; Zheng, N.; Yu, J.; Chen, R.; Kyaw, A. K. K.; Sun, X. W. High-Performance Quasi-2D Perovskite Solar Cells with Power Conversion Efficiency over 20% Fabricated in Humidity-Controlled Ambient Air. *Chem. Eng. J.* **2022**, *427*, 130949. https://doi.org/10.1016/j.cej.2021.130949.
(29) Yan, G.; Sui, G.; Chen, W.; Su, K.; Feng, Y.; Zhang, B. Selectively Fluorinated Benzylammonium-Based Spacer Cation Enables Graded Quasi-2D Perovskites for Efficient and Stable Solar Cells. *Chem. Mater.* **2022**, *34* (7), 3346–3356. https://doi.org/10.1021/acs.chemmater.2c00146.
(30) Correa-Baena, J.P.; Vagott, J.; Bairley, K.; Perini, C.A.R.; Mendez, A.F.C.; Hidalgo, J.; Lombardo, S.; Kacher, J; Lai, B. PbI_2 nanocrystal growth by atomic layer deposition of Pb $(tmhd)_2$ and HI, **2022**. https://doi.org/10.26434/chemrxiv-2022-0nq0v.
(31) Zhou, J.; Chu, Y.; Huang, J. Photodetectors Based on Two-Dimensional Layer-Structured Hybrid Lead Iodide Perovskite Semiconductors. *ACS Appl. Mater. Interfaces* **2016**, *8* (39), 25660–25666. https://doi.org/10.1021/acsami.6b09489.
(32) Dong, R.; Lan, C.; Xu, X.; Liang, X.; Hu, X.; Li, D.; Zhou, Z.; Shu, L.; Yip, S.; Li, C.; Tsang, S.-W.; Ho, J. C. Novel Series of Quasi-2D Ruddlesden–Popper Perovskites Based on Short-Chained Spacer Cation for Enhanced Photodetection. *ACS Appl. Mater. Interfaces* **2018**, *10* (22), 19019–19026. https://doi.org/10.1021/acsami.8b03517.
(33) Chen, S.; Teng, C.; Zhang, M.; Li, Y.; Xie, D.; Shi, G. A Flexible UV–Vis–NIR Photodetector Based on a Perovskite/Conjugated-Polymer Composite. *Adv. Mater.* **2016**, *28* (28), 5969–5974. https://doi.org/10.1002/adma.201600468.

(34) Tang, Y.; Liang, M.; Chang, B.; Sun, H.; Zheng, K.; Pullerits, T.; Chi, Q. Lead-Free Double Halide Perovskite Cs_3BiBr_6 with Well-Defined Crystal Structure and High Thermal Stability for Optoelectronics. *J. Mater. Chem. C* **2019**, *7* (11), 3369–3374. https://doi.org/10.1039/C8TC05480K.

(35) Wang, J.; Li, J.; Lan, S.; Fang, C.; Shen, H.; Xiong, Q.; Li, D. Controllable Growth of Centimeter-Sized 2D Perovskite Heterostructures for Highly Narrow Dual-Band Photodetectors. *ACS Nano* **2019**, *13* (5), 5473–5484. https://doi.org/10.1021/acsnano.9b00259.

(36) Li, X.; Yu, D.; Cao, F.; Gu, Y.; Wei, Y.; Wu, Y.; Song, J.; Zeng, H. Healing All-Inorganic Perovskite Films via Recyclable Dissolution–Recyrstallization for Compact and Smooth Carrier Channels of Optoelectronic Devices with High Stability. *Adv. Funct. Mater.* **2016**, *26* (32), 5903–5912. https://doi.org/10.1002/adfm.201601571.

(37) Lu, J.; Carvalho, A.; Liu, H.; Lim, S. X.; Castro Neto, A. H.; Sow, C. H. Hybrid Bilayer WSe_2–$CH_3NH_3PbI_3$ Organolead Halide Perovskite as a High-Performance Photodetector. *Angew. Chemie* **2016**, *128* (39), 12124–12128. https://doi.org/10.1002/ange.201603557.

(38) Wang, F.; Mei, J.; Wang, Y.; Zhang, L.; Zhao, H.; Zhao, D. Fast Photoconductive Responses in Organometal Halide Perovskite Photodetectors. *ACS Appl. Mater. Interfaces* **2016**, *8* (4), 2840–2846. https://doi.org/10.1021/acsami.5b11621.

(39) Ma, C.; Shi, Y.; Hu, W.; Chiu, M.; Liu, Z.; Bera, A.; Li, F.; Wang, H.; Li, L.; Wu, T. Heterostructured WS_2/$CH_3NH_3PbI_3$ Photoconductors with Suppressed Dark Current and Enhanced Photodetectivity. *Adv. Mater.* **2016**, *28* (19), 3683–3689. https://doi.org/10.1002/adma.201600069.

(40) Welch, P. The Use of Fast Fourier Transform for the Estimation of Power Spectra: A Method Based on Time Averaging over Short, Modified Periodograms. *IEEE Trans. Audio Electroacoust.* **1967**, *15* (2), 70–73. https://doi.org/10.1109/TAU.1967.1161901.

(41) Straus, D. B.; Iotov, N.; Gau, M. R.; Zhao, Q.; Carroll, P. J.; Kagan, C. R. Longer Cations Increase Energetic Disorder in Excitonic 2D Hybrid Perovskites. *J. Phys. Chem. Lett.* **2019**, *10* (6), 1198–1205. https://doi.org/10.1021/acs.jpclett.9b00247.

(42) Song, J.; Feng, X.; Wei, H.; Yang, B. Supramolecular Interactions of Flexible 2D Perovskite in Microstrain Releasing and Optoelectronic Properties Recovery. *Adv. Funct. Mater.* **2022**, *32* (30), 2203329. https://doi.org/10.1002/adfm.202203329.

(43) Tremblay, M.-H.; Bacsa, J.; Zhao, B.; Pulvirenti, F.; Barlow, S.; Marder, S. R. Structures of $(4\text{-Y-}C_6H_4CH_2NH_3)_2PbI_4$ {Y = H, F, Cl, Br, I}: Tuning of Hybrid Organic Inorganic Perovskite Structures from Ruddlesden–Popper to Dion–Jacobson Limits. *Chem. Mater.* **2019**, *31* (16), 6145–6153. https://doi.org/10.1021/acs.chemmater.9b01564.

(44) Wang, P.-X.; Najarian, A. M.; Hao, Z.; Johnston, A.; Voznyy, O.; Hoogland, S.; Sargent, E. H. Structural Distortion and Bandgap Increase of Two-Dimensional Perovskites Induced by Trifluoromethyl Substitution on Spacer Cations. *J. Phys. Chem. Lett.* **2020**, *11* (23), 10144–10149. https://doi.org/10.1021/acs.jpclett.0c02909.

(45) Paek, S.; Roldán-Carmona, C.; Cho, K. T.; Franckevičius, M.; Kim, H.; Kanda, H.; Drigo,

N.; Lin, K.; Pei, M.; Gegevičius, R.; Yun, H. J.; Yang, H.; Schouwink, P. A.; Corminboeuf, C.; Asiri, A. M.; Nazeeruddin, M. K. Molecular Design and Operational Stability: Toward Stable 3D/2D Perovskite Interlayers. *Adv. Sci.* **2020**, *7* (19), 2001014. https://doi.org/10.1002/advs.202001014.

(46) Luo, B.; Guo, Y.; Xiao, Y.; Lian, X.; Tan, T.; Liang, D.; Li, X.; Huang, X. Fluorinated Spacers Regulate the Emission and Bandgap of Two-Dimensional Single-Layered Lead Bromide Perovskites by Hydrogen Bonding. *J. Phys. Chem. Lett.* **2019**, *10* (17), 5271–5276. https://doi.org/10.1021/acs.jpclett.9b02172.

(47) García-Benito, I.; Quarti, C.; Queloz, V. I. E.; Hofstetter, Y. J.; Becker-Koch, D.; Caprioglio, P.; Neher, D.; Orlandi, S.; Cavazzini, M.; Pozzi, G.; Even, J.; Nazeeruddin, M. K.; Vaynzof, Y.; Grancini, G. Fluorination of Organic Spacer Impacts on the Structural and Optical Response of 2D Perovskites. *Front. Chem.* **2020**, *7*, 946. https://doi.org/10.3389/fchem.2019.00946.

(48) Chan, K. L., Master's thesis, National Taipei University of Technology, Taipei, 2022. (Note: the crystals of $(3F\text{-}PPH)_2PbI_2Br_2$ have been recently grown.).

(49) Niu, T.; Lu, J.; Jia, X.; Xu, Z.; Tang, M.C.; Barrit, D.; Yuan, N.; Ding, J.; Zhang, X.; Fan, Y.; Luo, T. Interfacial engineering at the 2D/3D heterojunction for high-performance perovskite solar cells. *Nano lett.* **2019**, *19* (10), 7181-7190. https://doi.org/10.1021/acs.nanolett.9b02781.

(50) Min, L.; Tian, W.; Cao, F.; Guo, J.; Li, L. 2D Ruddlesden–Popper Perovskite with Ordered Phase Distribution for High-Performance Self-Powered Photodetectors. *Adv. Mater.* **2021**, *33* (35), 2170274. https://doi.org/10.1002/adma.202101714.

(51) Wang, L.; Zhou, Q.; Zhang, Z.; Li, W.; Wang, X.; Tian, Q.; Yu, X.; Sun, T.; Wu, J.; Zhang, B; Gao, P., A guide to use fluorinated aromatic bulky cations for stable and high-performance 2D/3D perovskite solar cells: The more fluorination the better? *J. Energy Chem.* **2022**, *64 (2)*, 179-189. https://doi.org/10.1016/j.jechem.2021.04.063.
